# Systematic identification of factors mediating accelerated mRNA degradation in response to changes in environmental nitrogen

**DOI:** 10.1371/journal.pgen.1007406

**Published:** 2018-05-21

**Authors:** Darach Miller, Nathan Brandt, David Gresham

**Affiliations:** Center for Genomics and Systems Biology, Department of Biology, New York University, New York, New York, United States of America; Pacific Northwest Research Institute, UNITED STATES

## Abstract

Cellular responses to changing environments frequently involve rapid reprogramming of the transcriptome. Regulated changes in mRNA degradation rates can accelerate reprogramming by clearing or stabilizing extant transcripts. Here, we measured mRNA stability using 4-thiouracil labeling in the budding yeast *Saccharomyces cerevisiae* during a nitrogen upshift and found that 78 mRNAs are subject to destabilization. These transcripts include Nitrogen Catabolite Repression (NCR) and carbon metabolism mRNAs, suggesting that mRNA destabilization is a mechanism for targeted reprogramming of the transcriptome. To explore the molecular basis of destabilization we implemented a SortSeq approach to screen the pooled deletion collection library for *trans* factors that mediate rapid *GAP1* mRNA repression. We combined low-input multiplexed Barcode sequencing with branched-DNA single-molecule mRNA FISH and Fluorescence-activated cell sorting (BFF) to identify the Lsm1-7p/Pat1p complex and general mRNA decay machinery as important for *GAP1* mRNA clearance. We also find that the decapping modulators *EDC3* and *SCD6*, translation factor eIF4G2, and the 5’ UTR of *GAP1* are factors that mediate rapid repression of *GAP1* mRNA, suggesting that translational control may impact the post-transcriptional fate of mRNAs in response to environmental changes.

## Author summary

Budding yeast rapidly increase their rate of growth upon re-feeding of glutamine to a nitrogen-limited culture. Within the first several minutes, the transcriptome begins to reprogram. We investigated changes in mRNA stability using a 4-thiouracil label-chase experiment to track the extant transcriptome during the nitrogen upshift. We found that a functionally enriched set of mRNA are destabilized, including some members of the Nitrogen Catabolite Repression regulon. To find genetic factors that may be involved, we used *GAP1* mRNA as a marker of this post-transcriptional regulation and developed a method for estimating mRNA abundance using a combination of mRNA FISH, FACS, and Barcode sequencing. We identified the Lsm1-7p/Pat1p complex as having large differences in *GAP1* mRNA regulation, and further work identified that the decapping modulators *EDC3* and *SCD6*, the translation initiation factor *eIF4G2*, and the 5’ UTR of *GAP1* have defects in rapidly clearing *GAP1* mRNA during this transition. Our study suggests that changes in the translational status of specific mRNAs may impact their stability in response to environmental signals.

## Introduction

Regulated changes in mRNA abundance are a primary cellular response to external stimuli. Both the rate of synthesis and the rate of degradation determine the steady-state abundance of a particular mRNA and the kinetics with which abundance changes occur [1, 2]. Changes in mRNA degradation rates fulfill an important mechanistic role in diverse systems, including development [3, 4] and disease [5]. In budding yeast, the rate of mRNA degradation is affected by environmental stresses [6], cellular growth rate [7], as well as by improvements in nutrient conditions [8].

Environmental shifts trigger rapid reprogramming of the budding yeast transcriptome in response to stresses and nutritional changes [9, 10]. Changes in mRNA degradation rates have been shown to play a role in responses to heat-shock, osmotic stress, pH increases, and oxidative stress [6, 11–13]. In response to these diverse stresses destabilization of mRNAs encoding ribosomal-biogenesis gene products, and stress-induced mRNA occurs [6]. Simultaneous increases in both synthesis and degradation rates of some mRNAs may serve to speed the return to a steady-state following a transient pulse of regulation [14]. Addition of glucose to carbon-limited cells results in both stabilization of ribosomal protein mRNAs [15] and destabilization of gluconeogenic transcripts [16, 17]. Destabilization of transcripts can have a delayed effect on reducing protein levels compared to up-regulated genes [18]. This suggests that accelerated mRNA degradation may serve additional purposes. For example, clearance of specific mRNAs could increase nucleotide pools [19] or facilitate reallocation of translational capacity [20–22].

Yeast cells metabolize a wide variety of nitrogen sources, but preferentially assimilate and metabolize specific nitrogen compounds. Transcriptional regulation, known as “nitrogen catabolite repression” (NCR) [23], controls the expression of mRNAs encoding transporters, metabolic enzymes, and regulatory factors required for utilization of alternative nitrogen sources. NCR-regulated transcripts are expressed in the absence of a readily metabolized (preferred) nitrogen sources or in the presence of growth-limiting concentrations (in the low μM range) of any nitrogen source [24, 25]. Regulation of NCR targets is mediated by two activating GATA transcription factors, Gln3p and Gat1p, and two repressing GATA factors, Dal80p and Gzf3p. *GAT1*, *GZF3*, and *DAL80* promoters contain GATAA motifs, and thus transcriptional regulation of NCR targets entails self-regulatory and cross-regulatory loops. When supplied with a preferred nitrogen source such as glutamine, the NCR-activating transcription factors Gat1p and Gln3p are excluded from the nucleus by TORC1-dependent and -independent mechanisms [26–28] and NCR transcripts are strongly repressed. The activity of some NCR gene products is also controlled by post-translational mechanisms [29] such as the General Amino-acid Permease (Gap1p) which is rapidly inactivated upon a nitrogen upshift via ubiquitination [30–32]. Recently, we have identified an additional level of regulation of NCR transcripts: cells growing in NCR de-repressing conditions accelerate the degradation of *GAP1* mRNA upon addition of glutamine [25]. Thus, mRNA degradation rate regulation may be an additional mechanism for clearing NCR-regulated transcripts upon improvements in environmental nitrogen availability.

Multiple pathways mediate the degradation of mRNAs. The main pathway of mRNA degradation occurs by deadenylation and decapping prior to 5’ to 3’ exonucleolytic degradation by Xrn1p; however, transcripts are also degraded 3’ to 5’ via the exosome, or via activation of co-translational quality control mechanisms [33]. Deadenylation of mRNAs by the Ccr4-Not complex allows the mRNA to be bound at the 3’ end by the Lsm1-7p/Pat1p complex, a heptameric ring comprising the SM-like proteins Lsm2-7p and the cytoplasmic-specific Lsm1p [34, 35], which then recruits factors for decapping by Dcp2p. Recruitment of the decapping enzyme [36] is the rate-limiting step for canonical 5’-3’ degradation. Therefore Lsm1-7p, Pat1p, and associated factors play a key role in determining the kinetics of mRNA degradation [37].

Regulation of mRNA degradation pathways can alter the stability of specific mRNAs. For example, the RNA-binding protein (RBP) Puf3p recognizes a *cis*-element in 3’ UTRs [38] and affects mRNA degradation rates depending on Puf3p phosphorylation status [39]. In addition to *cis*-elements within the transcript, promoters have been shown to mark certain RNA-protein (RNP) complexes to specify their post-transcriptional regulation [17, 40–42]. These mechanisms may be controlled by a variety of different signalling pathways including Snf1 [43, 44], PKA [45], Phk1/2 [46], and TORC1 [47]. Thus, regulated changes in mRNA degradation rates entails numerous mechanisms that collectively tune stability of mRNAs in response to the activity of signalling pathways.

Here, we studied the global regulation of mRNA degradation rates upon an increase in nitrogen availability using 4-thiouracil (4tU) label-chase and RNAseq. We found that a set of 78 mRNAs are subject to accelerated mRNA degradation, including many NCR transcripts as well as mRNAs encoding components of carbon metabolism. To identify the mechanism underlying accelerated mRNA degradation we designed a high-throughput genetic screen using Barcode-sequencing of a pooled library which was fractionated using Fluorescence-activated cell sorting on the basis of single molecule mRNA FISH signal (BFF). We screened the barcoded yeast deletion collection to test the effect of each gene deletion on the abundance of *GAP1* mRNA in NCR de-repressing conditions and its clearance following the addition of glutamine. We find that the Lsm1-7p/Pat1p complex and decapping modifiers affect both *GAP1* mRNA steady-state expression and its accelerated degradation. This work expands our understanding of mRNA stability regulation in remodeling the transcriptome during a relief from growth-limitation and demonstrates a generalizable approach to the study of genetic determinants of mRNA dynamics.

## Results

### Transcriptional reprogramming precedes physiological remodeling

**Fig 1:**
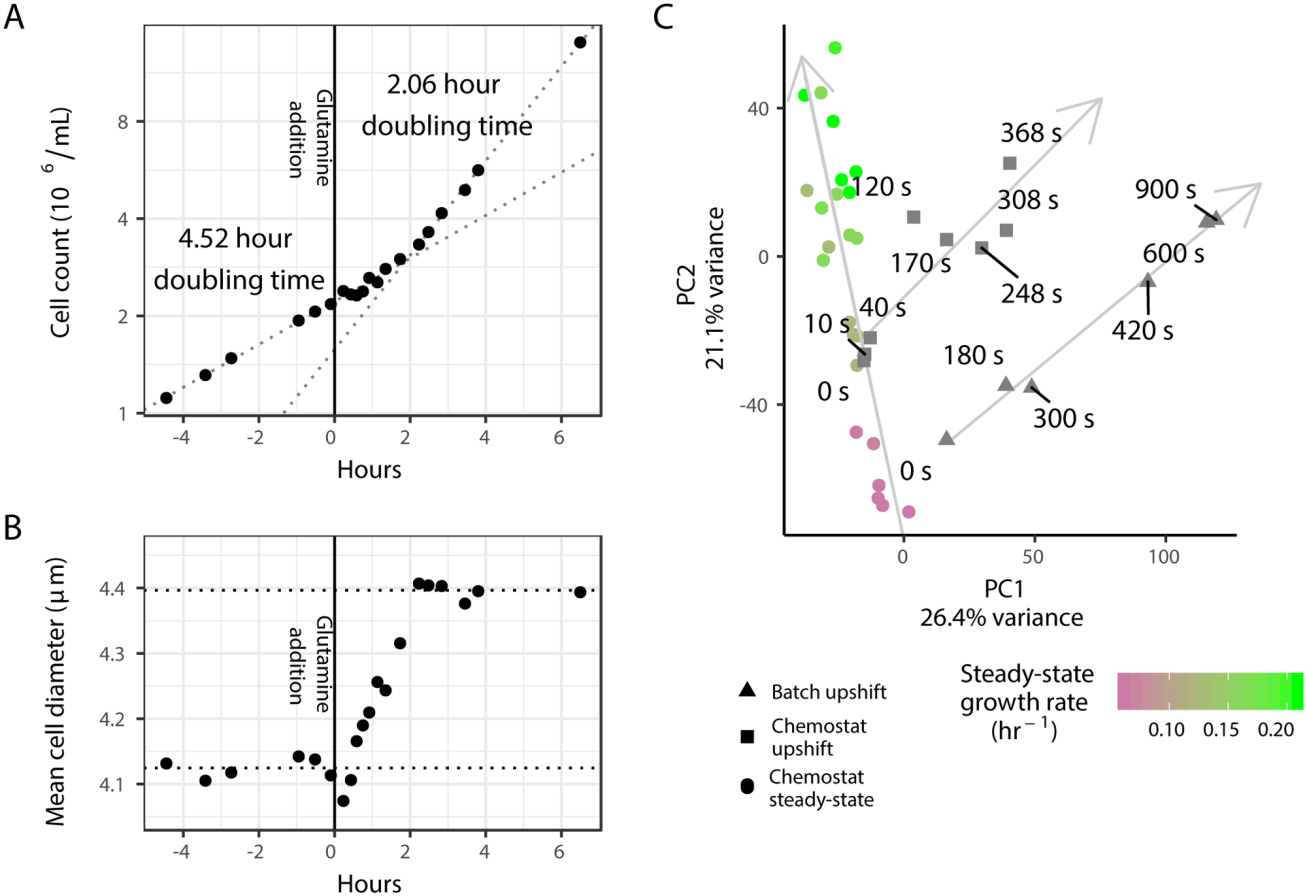
Dynamics of physiology and transcriptome remodeling during a nitrogen upshift. **A)** 400μM glutamine was added to a culture of yeast cells growing in minimal media containing 800μM proline as a sole nitrogen source. Measurements of culture density across the upshift are plotted. Dotted lines denote linear regression of the natural log of cell density against time before the upshift and after the 2 hour lag. **B)** Average cell size during the same experiment. Dotted lines denote the mean cell diameter before the upshift and after the 2 hour lag. **C)** PCA analysis of global mRNA expression in steady-state chemostats and following an upshift [25]. Steady-state nitrogen-limited chemostat cultures maintained at different growth rates (colored circles) primarily vary along principal component 2. Expression following a nitrogen-upshift in either a chemostat (squares) or batch culture (triangles) show similar trajectories and vary along both components. Grey lines depict the major trajectory of variation for steady-state and upshift experiments.

Cellular responses to environmental signals entail coordinated changes in both gene expression and cellular physiology. Previously, we studied the steady-state and dynamic responses of *Saccharomyces cerevisiae* (budding yeast) to environmental nitrogen [25], and found that the transcriptome is rapidly reprogrammed following a single pulsed addition of glutamine to nitrogen-limited cells in either a chemostat or batch culture. To study physiological changes in response to a nitrogen upshift, we measured growth rates of a population of cells. A prototrophic haploid lab strain (FY4, isogenic to S288c) grows with a 4.5 hour doubling time in batch culture in minimal media containing proline as a sole nitrogen source (Fig 1A). Upon addition of 400μM glutamine the cells undergo a 2-hour lag period during which no change in population growth rate is detected, but the average cell size continuously increases (∼21% increase in mean volume Fig 1B). Following the 2-hour lag, the population adopts a 2.1 hour doubling time. By contrast, global gene expression changes are detected within three minutes of the upshift [25]. Thus, transcriptome remodeling precedes physiological remodeling in response to a nitrogen upshift.

To evaluate concordance in transcriptome remodeling between chemostat and batch nitrogen upshifts, and the extent to which they reflect changes in gene expression observed during systematic steady-state changes in growth rates using chemostats, we performed principal component analysis of global gene expression (Fig 1C). The first two principal components, which account for almost half of the total variation, clearly separate steady-state and nitrogen upshift cultures. Systematic changes in growth rate primarily results in separation of gene expression states along the second principal component, whereas upshift experiments vary along the first principal component. This suggests that although a nitrogen upshift results in a gene expression state reflecting increased cell growth rates [25], the transcriptome is remodeled through a distinct state. In upshift experiments in chemostats, the gene expression trajectory ultimately returns to the initial steady-state condition as excess nitrogen is depleted by consumption and dilution (S1 and S3 Figs).

To investigate the functional basis of gene expression programs in the upshift and steady-state conditions, we computed the correlation of each transcript with the loadings on these first two principal components and performed gene-set enrichment analysis (S2 Table). Component 1 is positively correlated with functions including mRNA processing, transcription from RNA polymerases (I,II,and III), and chromatin organization, and negatively correlated with cytoskeleton organization, vesicle organization, membrane fusion, and cellular respiration. Both steady-state and upshift gene expression trajectories increase with principal component 2, but they diverge along principal component 1. Components 1 and 2 are strongly enriched for terms including ribosome biogenesis, nucleolus, and tRNA processing, and negatively correlated with vacuole, cell cortex, and carbohydrate metabolism terms. Together, this analysis suggests that both upshift and increased steady-state growth rates share upregulation of ribosome-associated components, but the reprogramming preceeding the upshift in growth reflects an immediate focus on quantitative changes in gene expression machinery instead of structural cellular components. Importantly, dynamic reprogramming is similar in both the chemostat and batch upshift (Fig 1C). As batch cultures are a technically simpler experimental system, we performed all subsequent experiments using batch culture nitrogen upshifts.

### Global analysis of mRNA stability changes during the nitrogen upshift

Previously, we found that *GAP1* and *DIP5* mRNAs are destabilized in response to a nitrogen upshift [25]. We sought to determine if mRNA destabilization is specific to NCR transporter mRNAs by measuring global mRNA stability across the upshift using 4-thiouracil (4tU) labeling and RNA-seq [48, 49]. As a 4tU labeling experiment requires uracil transport, which may be altered upon stresses or a change in nitrogen-availability [50, 51], we designed experiments such that the chase was initiated prior to addition of glutamine or water (mock). We normalized data using *in vitro* synthesized thiolated spike-ins by fitting a log-linear model to spike-in counts across time (S1 Appendix), which reduced noise and increased our power to detect stability changes (S3, S4 and S5 Tables). Data and models for each transcript can be visualized in browser using a Shiny appplication (http://shiny.bio.nyu.edu/users/dhm267/).

We modeled the log-transformed normalized signal for each mRNA using linear regression (S6 Table). Of 4,859 mRNAs measured we identified 94 that increased in degradation rate and 38 that decreased (FDR < 0.01, using [52]). We generated a model of nucleotide labeling kinetics to assess the effect of an incomplete label chase on our experimental results (S1 Appendix), and found that complete transcriptional inhibition alone could theoretically result in a 13.3% increase in the apparent degradation rate. In order to eliminate the possibility that rapid synthesis changes could affect our estimates, we considered transcript destabilization to be at least a doubling (100% increase) in the apparent degradation rates between pre-upshift and post-upshift. This conservative cutoff left 78 mRNA that are significantly destabilized upon a nitrogen upshift.

**Fig 2:**
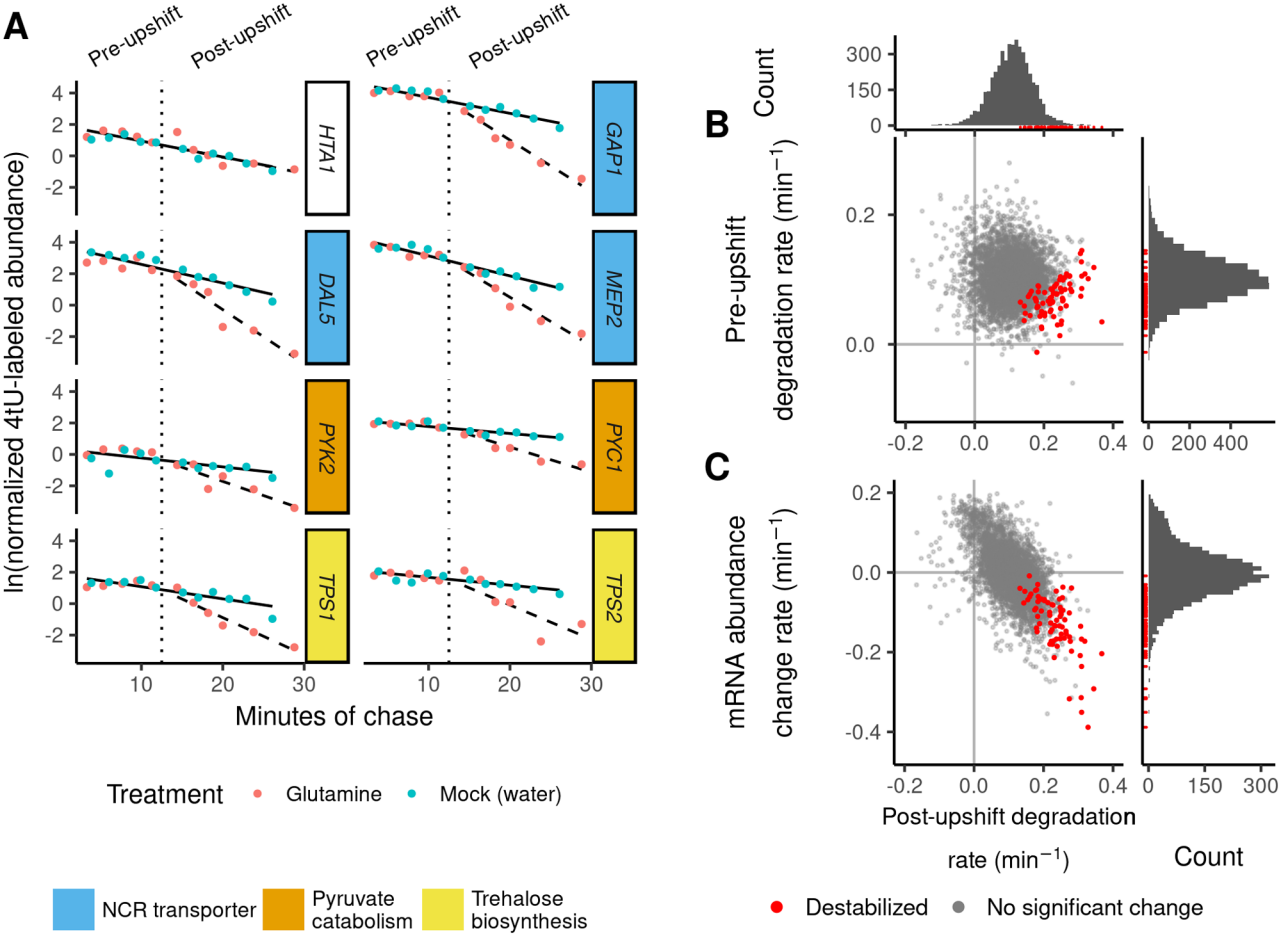
Global mRNA stability changes following a nitrogen upshift. **A)** 4tU-labeled mRNA from each gene was measured over time, before and after the addition (vertical dotted line) of glutamine (nitrogen-upshift) or water (mock). Linear regression models were fit to the data with a rate before the upshift (solid line) and a change in rate after glutamine addition (dashed line). *HTA1* is not significantly destabilized, whereas mRNAs encoding NCR-regulated transporters or pyruvate and trehalose metabolism components are significantly destabilized. Plots for all genes are available in the associated Shiny application (Methods). **B)** Comparison between the pre-upshift mRNA degradation rate (y-axis) and the post-upshift mRNA degradation rate (x-axis). Details of modeling are in S1 Appendix. **C)** Comparison between changes in mRNA expression following upshift [25] (y-axis) and the post-upshift degradation rate (x-axis). Transcripts that are significantly destabilized are colored red, and shown with red rug-marks in the marginal histograms.

The vast majority of transcripts (4,781 of 4,859) do not show evidence for stability changes upon addition of glutamine (e.g. *HTA1*, Fig 2A). The median pre-upshift half-life is 6.92 minutes and the median half-life following the upshift is 6.32 minutes (Table 1) suggesting that there is not a global change in mRNA stability. Global stability estimates are considerably lower than previous estimates in rich medium [48, 49, 53], which may reflect the different nutrient conditions used in our study. The 78 transcripts significantly destabilized upon the glutamine-upshift include mRNAs encoding NCR transporters *GAP1*, *DAL5*, and *MEP2* (blue label, Fig 2A), the pyruvate metabolism enzymes *PYK2* and *PYC1* (orange label), and trehalose synthase subunits *TPS1* and *TPS2* (yellow label). Destabilized mRNA tend to be more stable before the upshift (Fig 2B), (median half-life of 9.46 minutes) and exhibit a median 3.06-fold increase in degradation rates (median half-life of 3.02 minutes following the upshift).

**Table 1:** Summary of mRNA stability and changes upon the upshift. Shown here are the median rates or changes in rates for the specified sets. Destabilized transcripts were identified using the criteria of a significant (FDR < 0.01) change in estimated degradation rates and at least a doubling of the rate of clearance.

	Pre-shift		Post-shift		Change in specific rate (min^−1^)	Fold-change specific rate
	specific rate (min^−1^)	half-life (min)	specific rate (min^−1^)	half-life (min)		
All transcripts	0.100	6.92	0.110	6.32	0.00865	1.08
Destabilized (n = 78)	0.0732	9.46	0.229	3.02	0.158	3.06
No difference detected (n = 4781)	0.101	6.89	0.108	6.40	0.00728	1.07

We tested for functional enrichment among the set of 78 destabilized mRNAs and found that they are strongly enriched for NCR transcripts (16 of 78, p < 10^−11^). Almost half of the destabilized transcripts are annotated as “ESR-up” genes (S5 Fig), on the basis of their rapid induction during the environmental stress response [9]. 78 destabilized mRNAs are enriched (FDR < 0.05) for additional GO terms and KEGG pathways (S7 Table) including glycolysis/gluconeogenesis (6/78 genes), carbohydrate metabolic process (24/78 genes), trehalose-phosphatase activity (3/78 genes), pyruvate metabolic process (6/78 genes), and secondary active transmembrane transport (8/78 genes, a subset of the enriched 11 ion transmembrane transport genes). Thus destabilization upon a nitrogen upshift regulates, but is not restricted to, NCR-regulated mRNAs and reflects broader metabolic changes in the cell.

To investigate the extent to which mRNA stability changes contribute to transcriptome reprogramming, we compared degradation rates to abundance changes [25] following the upshift (Fig 2C). Changes in mRNA degradation rates and expression change rates are anti-correlated (Pearson’s *r* = -0.598, p-value < 10^−15^, S4 Fig), consistent with stability changes impacting gene expression dynamics. However, they are not entirely co-incident, as some destabilized transcripts do not exhibit decreases in abundance (red points in Fig 2C, S6 and S7 Figs). This analysis indicates that increases in degradation rates play a significant role in the rapid reprogramming of the transcriptome upon a glutamine upshift, but that in some cases cases they are counteracted by increases in mRNA synthesis rates [6, 14].

Functional coordination of mRNA stability changes suggests a possible role for *cis*-element regulation. We analyzed UTRs and coding sequence for enrichment of new motifs or known RNA binding protein (RBP) motifs. 3’ UTRs of destabilized transcripts are depleted of Puf3p binding sites, but we found no enriched sequence motif in the 3’ UTRs. 5’ UTRs are enriched for a GGGG motif, which may be explained by convergence between mRNA stability changes and transcriptional control by Msn2/4 on the ESR “up” genes (S5 Fig, [6, 9]). 5’ UTRs are also enriched for binding motifs reported for Hrp1p (S9 Fig), a canonical member of the nuclear cleavage factor I complex [54]. However, this protein has been shown to shuttle to the cytoplasm where it may play a regulatory role [55–57]. On average, destabilized mRNAs are longer and contain more optimal codons (S8 Fig, [58]). Together, this analysis suggests that the mechanism of destabilization may act through cis elements in the 5’ UTR and/or biased codon usage.

### A genome-wide screen for trans-factors regulating GAP1 mRNA repression

**Fig 3:**
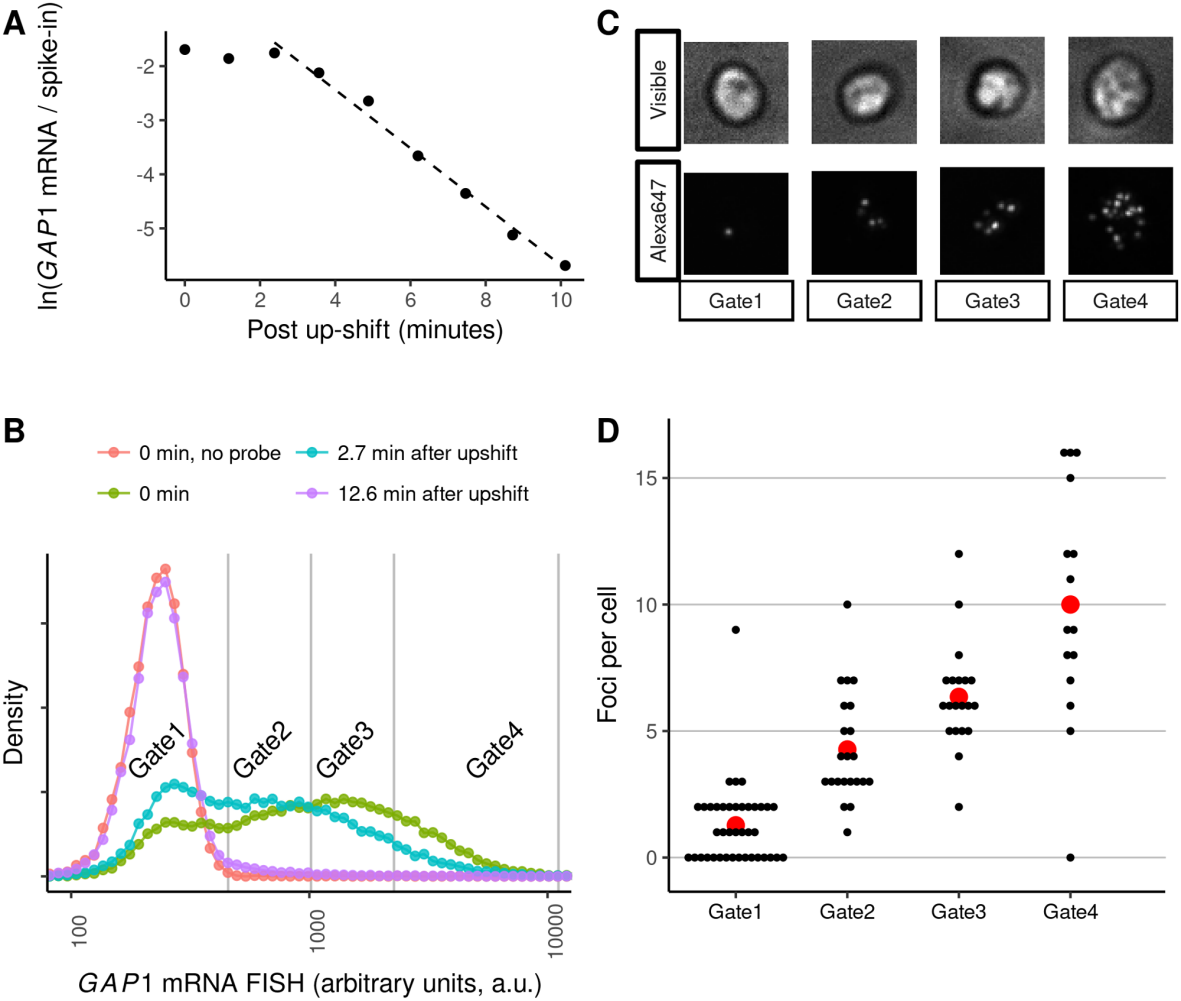
GAP1 mRNA dynamics measured by flow cytometry. **A)** GAP1 mRNA following upshift measured using RT-qPCR, relative to an external spike-in mRNA standard. The dashed line is fit to points 2 minutes after the upshift. **B)** Flow cytometry of wild-type yeast probed for *GAP1* mRNA in nitrogen-limited conditions and following an upshift. The vertical grey lines indicate FACS gate boundaries used for cell sorting. **C)** Representative cells from each bin sorted from the experiment in panel B. **D)** Quantification of microscopy data. Each black dot represents a single cell. The mean number of foci per cell in each bin from panel B is displayed as a red point.

We sought to identify *trans*-factors mediating accelerated mRNA degradation in response to a nitrogen upshift. We selected *GAP1* as representative of transcript destabilization, as it is abundant in nitrogen-limiting conditions and is rapidly cleared upon addition of glutamine (3.24-fold increase in degradation rate, Fig 3A, S6 Table). Previous approaches to high-throughput genetics of transcriptional activity have used protein expression reporters [59, 60] or automation of qPCR [61]. However, for our purposes, we required direct measurement of *GAP1* mRNA changes on a rapid timescale. Therefore, we applied single molecule fluorescent *in situ* hybridization (smFISH) to quantify native *GAP1* transcripts in yeast cells in the pooled prototrophic yeast deletion collection [62]. Using fluorescence activated cell sorting (FACS) and Barseq [63–65], we aimed to quantify and model the distribution of *GAP1* mRNA in each mutant [66, 67].

We found that individually labeled probes tiled across *GAP1* mRNA [68] were insufficiently bright for *GAP1* mRNA quantification using flow cytometry, likely due to the small cell size of nitrogen-limited cells and the low transcript numbers in yeast cells compared to mammalian cells [69]. Therefore, we used branched DNA probes (Quantigene), which serve to amplify the FISH signal [70]. We developed a fixation and permeabilization protocol (S2 Appendix) that enabled detection of the distribution of *GAP1* mRNA in steady-state nitrogen-limited conditions and its repression following the upshift (Fig 3B). In control experiments, we found that the signal is eliminated in a *GAP1* deletion strain or by omitting the targeting probe (Fig 3B and S10 Fig). To validate sorting, we sorted a sample of cells into quartiles and used microscopy to count fluorescent foci per cell (Fig 3C). We found that increased flow cytometry signal is associated with an increase in the number of foci in the cells (Fig 3D, *R*^2^ = 0.607, p < 10^−11^).

Previous SortSeq studies of the yeast deletion collection have used outgrowth to generate sufficient material for Barseq [60]. However, formaldehyde fixation precludes outgrowth. We found that below approximately 10^6^ templates, the Barseq reaction produces primer dimers that outcompete the intended PCR product (S2 Appendix). Therefore, we re-designed the PCR reaction [63, 64] to be robust for low sample inputs (S2 Appendix). Our protocol incorporates a 6-bp unique molecular identifier (UMI) into the first round of extension to identify PCR duplicates, and uses 3’-phosphorylated oligonucleotides and a strand-displacing polymerase (Vent exo-) to block primer dimer formation and off-target amplification. Because strain barcodes are of variable lengths, we developed a bioinformatic pipeline to extract barcodes and UMIs using pairwise alignment to invariant flanking sequences. Based on *in silico* benchmarks, this approach was robust to systematic and simulated random errors that can confound analysis of the yeast deletion barcodes (Availability of data and analysis scripts, S2 Appendix).

**Fig 4:**
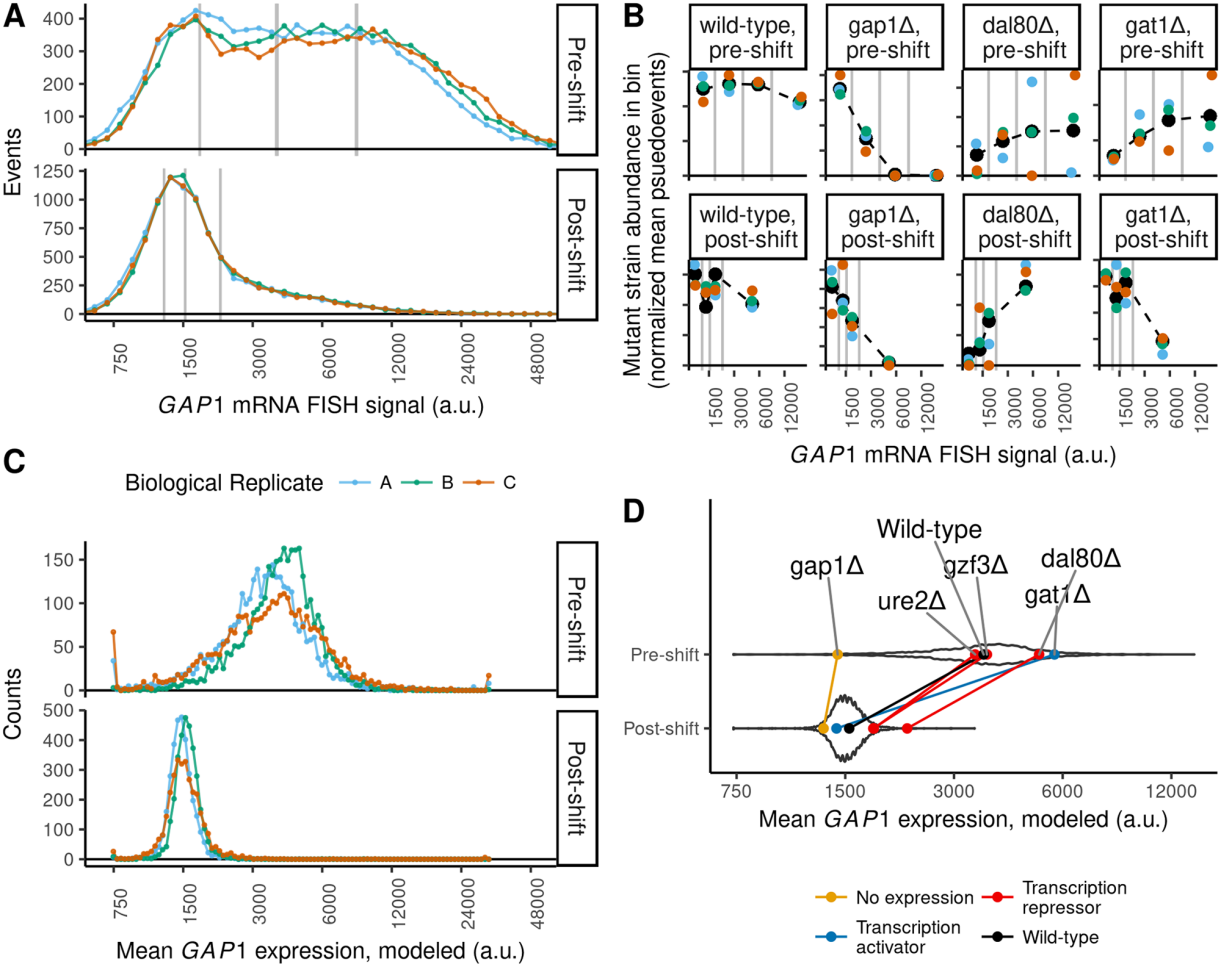
BFF estimates of GAP1 mRNA abundance per mutant. **A)** Flow cytometry analysis of *GAP1* mRNA abundance in the prototrophic deletion collection (n = 3,230 mutants) before and after the upshift. The vertical gray lines denote boundaries of the four FACS gates. Biological replicates are indicated by color. **B)** Measurements for individual genes before and after the upshift. Pseudo-events per strain per bin are on the y-axis. Black dashed lines indicate maximum-likelihood fits of a log-normal to pseudo-events within each bin for each mutant. For plotting purposes, points are positioned on the x-axis at the average signal for the library in that bin. Colors are as in panel A. **C)** Distribution of modeled mean GAP1 mRNA levels for each mutant. **D)** The mean *GAP1* mRNA expression levels fit using all replicate data for individual mutants before and after the upshift are shown as points connected by a line, colored according to the type of gene. The background violin plot shows the distribution of all 3,230 mutants. Plots for all mutants are available in the associated Shiny application (Methods).

We refer to this experimental approach as BFF (Barseq after FACS after FISH). We used BFF to estimate *GAP1* mRNA abundance for every mutant in the haploid prototrophic deletion collection [62] in nitrogen-limiting conditions and 10 minutes following the upshift. This approach facilitates identification of mutants with defects in mRNA regulation at both the transcriptional and post-transcriptional level without altering *GAP1* mRNA *cis*-elements that may affect its regulation. Moreover, this design enables identification of factors that regulate both the steady-state abundance of *GAP1* mRNA and its transcriptional repression following an upshift. We analyzed the deletion pool in biological triplicate (Fig 4A). Following barcode sequencing we found that UMIs approached saturation at a slower rate than expected for random sampling, consistent with PCR amplification bias (S12 Fig), and therefore we used an error correction model [71]. After filtering, we calculated a pseudo-events metric that approximates the number of each mutant sorted into each bin. Principal components analysis shows that the samples are separated primarily by FACS bin within each condition and biological replicates are clustered indicating that our approach reproducibly captures the variation of *GAP1* mRNA flow cytometry signal across the library (S11 Fig).

### Estimating GAP1 mRNA abundance for individual mutants

We estimated the distribution of *GAP1* mRNA for each mutant by modeling pseudo-events in each quartile as a log-normal distribution using likelihood maximization (Fig 4B). From model fits we estimated the mean expression value for each mutant and found that the distribution of means estimated for 3,230 strains (S9 Table, Fig 4C) recapitulates the overall distribution of flow cytometry signal (Fig 4A). To validate our approach we first examined strains for which we expected to have a specific phenotype and compared their mean expression level to the distribution of expression for the entire population (Fig 4D). We found that the wildtype genotype (*his3*Δ complemented by the spHis5 allele during library construction) has an expression level that is centrally located in the distribution both before and following the upshift. The *gap1*Δ genotype is a negative control and we estimate that it is at the extreme low end of the distribution before and following the upshift. Dal80p is a direct transcriptional repressor of NCR transcripts and we found that the *dal80*Δ genotype is defective in repression of *GAP1* before and after the upshift. Counter-intuitively, deletion of *GAT1*, a transcriptional activator of *GAP1*, appears to have higher steady-state expression of *GAP1* mRNA. However, increased expression of *GAP1* mRNA in a *gat1*Δ background has previously been reported [72] and is thought to result from the complex interplay of NCR transcription factors on their own expression levels. Data and models for each mutant strain can be visualized in browser using a Shiny appplication (http://shiny.bio.nyu.edu/users/dhm267/).

**Fig 5:**
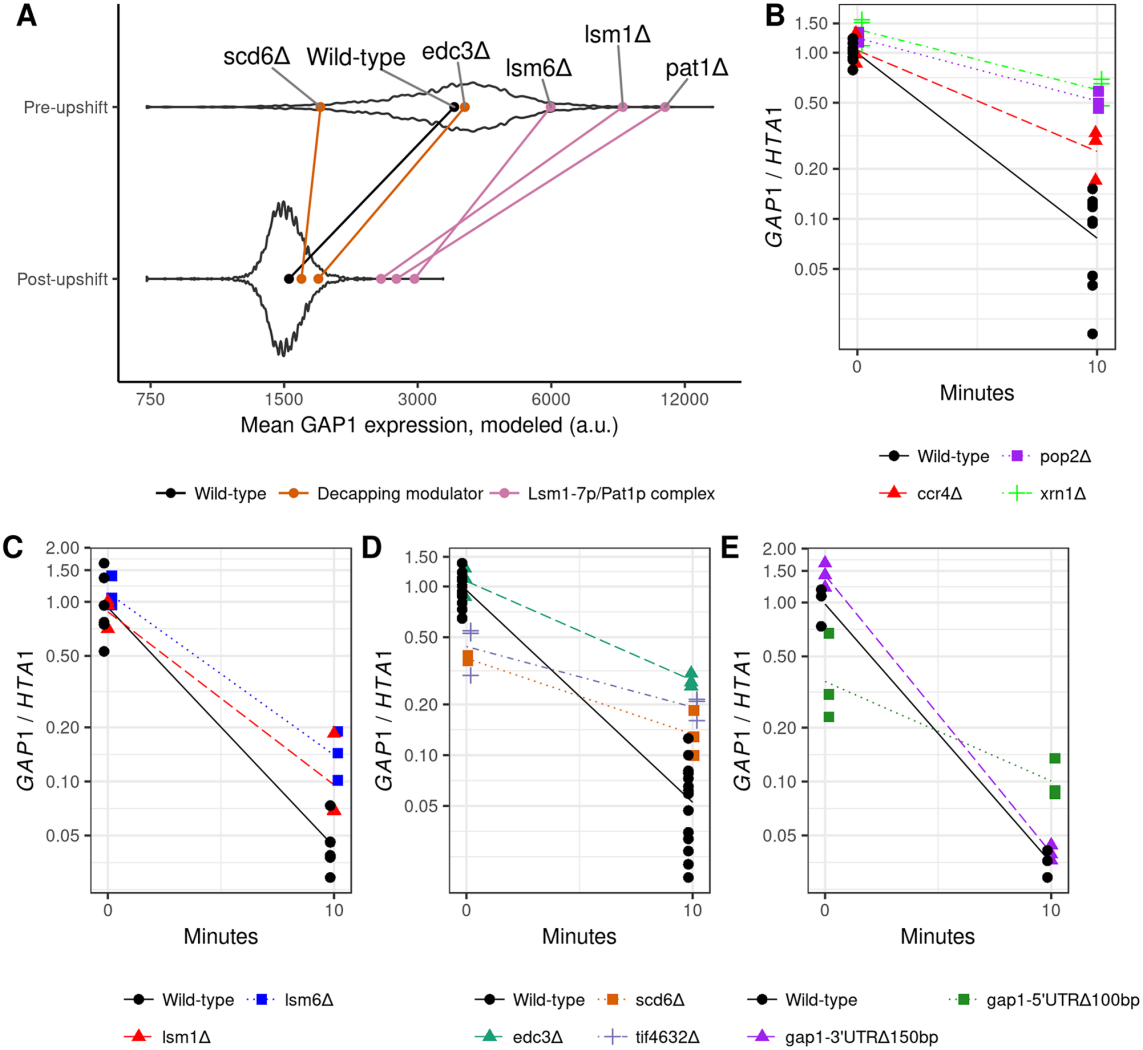
Disrupting the Lsm1-7p/Pat1p complex and translational regulation impairs clearance of GAP1 mRNA. **A)** In the background is the distribution of fit *GAP1* mRNA mean expression levels for all mutants in the pool. Indicated by colored points and lines are the means for individual knockout strains, as labeled. **B-E)**, *GAP1* mRNA relative to *HTA1* mRNA before and 10 minutes after a glutamine upshift, in biological replicates. Lines are a log-linear regression fit. Points are dodged horizontally for clarity, but timepoints for modeling and for drawn lines are 0 and 10 minutes exactly. Wild-type is FY4, and each estimate of the *GAP1*/*HTA1* ratio is normalized to the average ratio measured of FY4 at t = 0 for that qPCR batch. **B)** *xrn1*Δ, *ccr4*Δ, *pop2*Δ are all defective in *GAP1* mRNA clearance (p-values < 0.004). **C)** *lsm1*Δ and *lsm6*Δ are slowed in *GAP1* mRNA clearance (p-values < 0.0132 and 0.0299, respectively). **D)** *edc3*Δ is slowed in *GAP1* mRNA clearance (p-value < 10^−4^). *scd6*Δ and *tif4632*Δ are slowed in *GAP1* mRNA clearance (p-values < 10^−5^) and have lower levels of expression before the upshift (p-values < 0.003). **E)** A deletion of 150bp 3’ of *GAP1* stop codon has no significant effect, but a deletion of 100bp 5’ of the start codon has a defect in *GAP1* mRNA clearance (p-value < 10^−4^) and lower level of expression before the upshift (p-value < 0.0015).

To identify new cellular processes that regulate *GAP1* mRNA abundance, we used gene-set enrichment analysis (S10 Table). Following the upshift we found mutants that maintain high *GAP1* mRNA expression are enriched for negative regulation of gluconeogenesis (S13 Fig) and the Lsm1-7p/Pat1p complex (Fig 5A). Mutants in the TORC1 signalling pathway were not enriched; however, we found that a *tco89*Δ mutant has greatly increased *GAP1* mRNA expression before and after the upshift (S16 Fig), consistent with the repressive role of TORC1 on the NCR regulon. To compare expression before and after the upshift for each mutant, we regressed the post-upshift mean expression against the pre-upshift mean expression for each genotype (S15 Fig). We used the residuals for each strain to identify mutants that clear *GAP1* mRNA with kinetics slower than expected by this model. We found that the Lsm1-7p/Pat1p complex is again strongly enriched for slower than expected *GAP1* mRNA clearance (S9 Table). Specifically, the *lsm1*Δ, *lsm6*Δ, and *pat1*Δ strains have highly elevated *GAP1* mRNA expression before the upshift and are strongly impaired in the repression of *GAP1* mRNA after the upshift (Fig 5A).

As these factors are associated with processing-body dynamics, we tested if microscopically-observable processing-bodies form or disassociate during the upshift, using microscopy of Dcp2-GFP. We did not observe qualitative changes in Dcp2-GFP distribution (S18 Fig), and thus the upshift does not result in a microscopically visible changes in processing-body foci. This is consistent with previous investigations of amino-acid limitation stress [73] and suggests that the defects in *GAP1* mRNA clearance in mutants defective in the Lsm1-7p/Pat1p complex likely result from their roles in decapping or associated processes.

To confirm the role of the Lsm1-7p/Pat1p complex in clearing *GAP1* mRNA during the nitrogen upshift we measured *GAP1* mRNA repression using qPCR measurements normalized to *HTA1*, which is not subject to destabilization upon the upshift (Fig 2A). We also tested mutants that were not detected using BFF, or were only detected in the highest *GAP1* bin and therefore not suitable for modeling (e.g. *xrn1*Δ S16 Fig). Using this assay we found that the main 5’-3’ exonuclease *xrn1*Δ and mRNA deadenylase complex (*ccr4*Δ and *pop2*Δ) are impaired in *GAP1* mRNA repression (Fig 5B). We also found that qPCR confirmed results from the BFF assay. We confirmed that the accelerated degradation of *GAP1* mRNA is impaired in *lsm1*Δ and *lsm6*Δ (Fig 5C). We tested *scd6*Δ and *edc3*Δ, two modifiers of the decapping or processing-body assembly functions associated with this complex, and found two distinct phenotypes (Fig 5D). *edc3*Δ has similar expression as wild-type before the upshift, but clears *GAP1* mRNA much more slowly. *scd6*Δ has greatly reduced *GAP1* mRNA expression before the upshift but is impaired in *GAP1* mRNA clearance. Interestingly, *tif4632*Δ, a deletion of eIF4G2 known to interact with Scd6p [74], exhibits a similar phenotype.

Identification of an initiation factor subunit mutant with defects in *GAP1* mRNA clearance suggests that translation control may impact stability changes. Therefore we deleted the 5’ UTR and 3’ UTR of *GAP1*. Whereas the 3’ UTR deletion does not have an effect, the 5’ UTR deletion exhibits the phenotype of reduced *GAP1* mRNA before the upshift and a reduced rate of transcript clearance following the upshift (Fig 5E). We observed a similar phenotype with an independent deletion of 152bp upstream of the *GAP1* start codon (S17 Fig). These results indicate that *cis*-elements responsible for the rapid clearance of *GAP1* mRNA are unlikely to be located in the 3’ UTR and instead may be present in the 5’ end of the mRNA.

## Discussion

Regulated changes in mRNA stability allows cells to rapidly reprogram gene expression, clearing extant transcripts that are no longer required and potentially reallocating translational capacity. Pioneering work in budding yeast has shown that mRNA stability changes facilitate gene expression remodeling in response to changes in nutrient availability including changes in carbon sources [8] and iron starvation [75]. Here, we characterized genome-wide changes in mRNA stability in response to changes in nitrogen availability and identified factors that mediate the rapid repression of the destabilized mRNA, *GAP1*. Our study extends our previous work characterizing the dynamics of transcriptome changes using chemostat cultures [25] and shows that accelerated mRNA degradation targets a specific subset of the transcriptome in response to changes in nitrogen availability. We developed a novel approach to identify regulators of mRNA abundance using pooled mutant screens and find that modulators of decapping activity, and core degradation factors, are required for accelerated degradation of *GAP1* mRNA.

Measuring the stability of the transcriptome requires the ability to separate pre-existing and newly synthesized transcripts. We modified existing methods to measure post-transcriptional regulation of the yeast transcriptome in a nitrogen upshift using 4-thiouracil labeling [48, 49, 53]. These modifications entailed improved normalization and quantification of extant transcripts and explicit modeling of labelling dynamics to account for some of the inherent limitations of metabolic labeling approaches [2]. Continued development of fractionation biochemistry [76] and incorporation of explicit per-transcript efficiency terms will improve these methods further [77].

Our experiments show that the process of physiological and gene expression remodeling occur on very different timescales in response to a nitrogen upshift. Cellular physiology is remodeled over the course of two hours to achieve a new growth rate. By contrast, transcriptome remodeling occurs rapidly and through states that are distinct from those observed during systematic increases in steady-state growth rates. We have previously shown the rapid dynamics of NCR regulon repression upon a nitrogen upshift [25]. Our results here indicate that accelerated degradation of many NCR transcripts [24] contributes to this repression. Importantly, our results show that accelerated degradation is not limited to NCR transcripts but also targets transcripts enriched in carbon metabolism pathways, particularly pyruvate metabolism. Conversely, we also detect an apparent reduction in the degradation rate for some transcripts including *MAE1*. *MAE1* encodes an enzyme responsible for the conversion of malate to pyruvate, and combined with the accelerated degradation of *PYK2* mRNA may reflect the adoption of an adaptive shunt of carbon skeletons from glutamine to alanine via the TCA cycle [78]. A recent study described destabilization of carbon metabolism mRNAs after repletion of nitrogen following 16 hours of starvation [79]. We do not detect destabilization of *PGK1* mRNA in our study and note that the basal half-life of 6.2 minutes estimated in our study is similar to the accelerated rate reported by [79].

Destabilized transcripts are enriched for a binding motif of Hrp1p in the 5’ UTR. This essential component of mRNA cleavage for poly-adenylation in the nucleus has also been shown to shuttle to the cytoplasm and bind to amino-acid metabolism mRNAs [57]. In addition, *HRP1* has been shown to interact genetically to mediate nonsense-mediated decay (NMD) of a *PGK1* mRNA harboring a premature stop-codon [80] or a *cis*-element spanning the 5’ UTR and first 92 coding bp of *PPR1* mRNA [56]. A potential role for Hrp1p sites in the 5’ UTR of destabilized mRNAs warrants further investigation.

Using BFF we identified mutants in the Lsm1-7p/Pat1p complex as having elevated *GAP1* mRNA levels both before and after the upshift, consistent with their central role in mRNA metabolism. Out experiments using *GAP1* normalized to *HTA1* demonstrate that increased mRNA abundance before the upshift is likely a global effect (Fig 5C). However, these mutants have a defect in clearance of *GAP1* mRNA upon the upshift, pointing to their function as mRNA clearance factors. Additionally, we found that *edc3*Δ, *scd6*Δ, and *tif4632*Δ have impaired *GAP1* mRNA clearance. As these factors are involved in translational regulation they suggest a role for this process in the differential stability of *GAP1* mRNA (Fig 5D). Deletion of the 5’ UTR of *GAP1* also impairs its clearance (Fig 5E). The 5’ UTR deletions do not include the TATA box (ending at -179) or GATAA sites (nearest at -237) responsible for NCR GATA-factor regulation of *GAP1* [81]. Decreased steady-state expression of *GAP1* mRNA in 5’ UTR mutants suggests that interactions of factors with *cis*-elements in the 5’ UTR might be responsible for stabilizing *GAP1* mRNA during nitrogen limitation, although the truncation of the 5’ sequence may be enough to inhibit translation initiation by virtue of the shorter length [82]. Elements in the 5’ UTR have been demonstrated to modulate *GAL1* mRNA stability [83] and destabilize *SDH2* mRNA upon glucose addition, perhaps due to the competition between translation initiation and decapping mechanisms [16]. Interestingly, both *GAP1* and *SDH2* share the feature of a second start codon downstream of the canonical start [84] and we have previously found that mutation of the start codon of *GAP1* results in lower steady-state mRNA abundances [84]. This suggests a mechanism of degradation through dynamic changes in translation initiation that triggers decapping of *GAP1* and other mRNA. Future work interrogating this possible interaction of translational status and mRNA stability during dynamic conditions could also expand our understanding of the relationship between these two processes.

To our knowledge, this is the first time mRNA abundance has been directly estimated using a SortSeq approach, although using mRNA FISH and FACS to enrich subpopulations of cells has been previously reported [60, 69, 70]. This approach could be used with other barcoding mutagenesis technologies, like transposon-based or Cas9 mediated perturbations, to systematically test the genetic basis of transcript dynamics. The use of branched-DNA mRNA FISH, or other methods [85], allows for mRNA abundance estimation without requiring genetic manipulation which makes it suitable for a variety of applications. Furthermore, our methods for library construction from limited material should permit accurate quantification of pooled barcode libraries with small inputs, expanding the possibilities for flow cytometry markers to fixed-cell assays.

Why is *GAP1* subject to multiple layers of repression upon a nitrogen upshift, including at the level of transcript synthesis, transcript degradation, protein maturation, and post-translational inactivation? Given the strong fitness cost of inappropriate activity [31], this overlap could ensure mechanistic redundancy for robust repression in the face of phenotypic or genotypic variation. Alternatively, it could reflect a systematic need to free ribonucleotides or translational capacity, or some as yet uncharacterized process. Future work aimed at determining the adaptive basis of accelerated mRNA degradation will serve to illuminate the functional role of post-transcriptional gene expression regulation.

## Materials and methods

### Availability of data and analysis scripts

Computer scripts used for all analyses are available as a git repository on GitHub (https://github.com/darachm/millerBrandtGresham2018), sequencing fastq files are available in the Short Read Archive (SRP142613), and all data along with intermediate analysis files are available in zip archives archived in the Open Science Framework (https://osf.io/7ybsh/). With these archives, all analyses downstream of alignments can be re-run completely using the provided GNU Makefile. A Shiny application is also available to explore the two main datasets in this paper at http://shiny.bio.nyu.edu/users/dhm267/. It is also included in the OSF repository as a separate zipped archive for local installation and long-term archiving. Consult the README.md file in the git repository for more specific instructions, and the html_reports.zip archive for Rmarkdown-generated reports of all R analyses used.

### Media and upshifts of media

The nitrogen-limited media used is a minimal media supplemented with various salts, metals, minerals, vitamins, and 2% glucose, as previously described [25, 86]. For proline limitation, the media was made with 800μM L-proline as the sole nitrogen source. YPD media was made using standard recipes [87]. All growth was at 30°C, in an air-incubated 200rpm shaker using baffled flasks with foil caps, or roller drums for overnight cultures in test tubes. For glutamine upshift experiments, 400μM L-glutamine was added from a 100mM stock solution dissolved in MilliQ double-deionized water and filter sterilized. All upshift experiments were performed at a cell density of 1-5 million cells per mL, in nitrogen-limited media in which untreated cultures saturate at approximately 30 million cells per mL. For all experiments, a colony was picked from a YPD plate and grown in a 5mL nitrogen-limited (proline) pre-culture overnight at 30°C, then innoculated into the experimental culture from mid-exponential phase.

### Strains

See S11 Table for details. The wild-type strain used is FY4, a S288C derivative. The pooled deletion collection is as published [62]. For all experiments with single strains, colonies were struck from a -80°C frozen stock onto YPD (or YPD+G418 for deletion strains) to isolate single colonies. For pooled experiments we inoculated directly into nitrogen-limited (proline) media from aliquots of frozen glycerol stocks.

Strains with deletions 5’ of the start codon and 3’ of the stop codon were generated by the “delitto-perfetto” method [88], by inserting the pCORE-Kp53 cassette at either the 5’ or 3’ end of the coding sequence, then transforming with a short oligo product spanning the deletion junction and counter-selecting against the cassette using Gal induction of p53. Strains were generated and confirmed by Sanger sequencing, and traces are available in directory data/qPCRfollowup/ within the data zip archive (Availability of data and analysis scripts).

### Measurement of growth during upshift

A single colony of FY4 was inoculated in 5mL nitrogen-limited (proline) media and grown to exponential phase, then back diluted in nitrogen-limited (proline) media in a baffled flask. Samples were collected into an eppendorf, sonicated, diluted in isoton solution, and analyzed with a Coulter Counter Z2 (Beckman Coulter).

### Re-analysis of microarray data

Gene expression data [25] were analyzed using pcaMethods [89] to perform a SVD PCA on scaled data.

### qPCR

Samples were collected before, during the first ten minutes of the nitrogen upshift (Fig 3), or at ten minutes after the upshift (Fig 5). For the experiments described in Fig 5, all work was done in biological replicates. Each 10mL sample was collected by vacuum onto a 25mm nylon filter and frozen in liquid nitrogen. RNA was extracted by adding 400μL of TES buffer (10mM Tris (7.5pH), 10mM EDTA, 0.5% SDS) and 400μL of acid-phenol, vortexing vigorously and incubating at 65°C for an hour with vortexing every 20 minutes. For Fig 3 only, at the beginning of this extraction incubation we added 10μL of a 0.1ng/μL in-vitro synthesized spike-in mRNA BAC1200 (as generated for the label-chase RNAseq (S1 Appendix), but without 4-thiouridine). All samples were separated by centrifugation and extracted again with chloroform on a 2mL phase-lock gel tube (5Prime #2302830). After ethanol precipitation of the aqueous layer, RNA was treated DNAse RQ1 (Promega M610A) according to manufacturer instructions, then the reaction heat-killed at 65°C for 10 minutes after adding a mix of 1:1 0.5M EDTA and RQ1 stop-solution. The resulting RNA was cleaned with a phenol-chloroform extraction and ethanol precipitated. All samples were hybridized with RT primers by incubating the mixture at 80°C for 5 minutes then on ice for 5 minutes. For Fig 3 2μg RNA was primed with 2.08ng/μL random hexamers (Invitrogen 51709) and 2.5mM total dNTPs (Promega U1511), while for Fig 5 1μg RNA was primed with 5.6mM Oligo(dT)18 primers (Fermentas FERSO132) and 0.56mM total dNTPs (Promega U1511). These mixtures were combined with 1/10th 10x M-MulvRT buffer (NEB M0253L), 1/20th volume RNAse-OUT (Invitrogen 51535), and 1/20th volume M-MulvRT (NEB M0253L). A negative control with no reverse-transcriptase enzyme was also prepared and analyzed in the qPCR reaction. The reaction proceeded for 1 hour at 42°C, then was heat-killed at 90°C before diluting 1/8 with hyclone water (GE SH30538). This dilution was used as direct template in 10μL reactions with SybrGreen I Roche qPCR master-mix (Roche 04 707 516 001) for measurement on a Roche Lightcycler 480. For Fig 3, we used primers DGO230 and DGO232 to quantify *GAP1* and primers DGO605 and DGO606 to quantify the synthetic spike-in BAC1200. For Fig 5, we used primers DGO229 and DGO231 to quantify *GAP1* and primers DGO233 and DGO236 to quantify *HTA1*. See S12 Table for sequences. These were run on a Roche480 Lightcycler, with a max-second derivative estimate of the cycles-threshold (the *C*_*p*_ value output by analysis) used for analysis by scripts included in the git repo (Availability of data and analysis scripts). Linear regression of the log-transformed values was used to quantify the dynamics and assess significance of changes in expression levels or rates of change.

### Microscopy

Cells hybridized with *GAP1* mRNA FISH Affymetrix probes (as described in detail in S2 Appendix) were sorted with a BD FACSAria II based on emission area from a 660/20nm filter with a 633nm laser activation into four gates for the 3-minute post-shift timepoint. These were sorted using PBS sheath fluid at room-temperature into poly-propylene FACS tubes, vortexed and applied to poly-L-lysine-treated coverslips. Images were acquired on a DeltaVision scope, with FISH fluorescence detected in the “Cy5” channel (632/22nm excitation, 676/34nm emission) and the “Visible” light collected as bright-field illumination captured with a polarized objective. Raw images available in the “microscopy” zip archive (Availability of data and analysis scripts).

### Microscopy of Dcp2-GFP

To look for processing-body dynamics in response to a nitrogen upshift, we used strain DGY525, which is FY3 containing plasmid pRP1315 (gift from Roy Parker). Samples were collected before and following a nitrogen upshift (4, 10, 12, 19, or 25 minutes), from exponential growth in YPD, or 10 minutes after resuspending YPD-grown cells in DI water. All samples were collected by centrifugation at 10,000g for 30 seconds, aspirating most supernatant, then centrifugation for 20 seconds and aspirating all media. Each pellet was immediately resuspended in 4% PFA (diluted from EMS 16% PFA ampule RT15710) with 1x PBS (NaCl 8g/L, KCl 0.2g/L, Na_2_HPO_4_ 1.42g/L, KH_2_PO_4_ 0.24g/L) 15 minutes on bench, then spun at 10,000g for 1 minute, aspirated, then washed once and resuspended with 1x PBS. Samples were kept on ice, then put onto a coverslip for imaging on a DeltaVision scope. Raw images available in the microscopy zip archive (Availability of data and analysis scripts).

### 4tU label-chase and RNA sequencing

The methods and analysis are detailed in S1 Appendix, including protocols and manufacturer information, and all data and code are available as described (Availability of data and analysis scripts).

FY4 was grown in nitrogen-limitation conditions overnight with a 50μM:50μM mixture of 4-thiouracil:uracil. This culture was split, then 4mM uracil was added to chase the 4-thiouracil label (a 41-fold excess of uracil). 30mL samples of the culture were taken by filtration onto 25mm nylon filters and flash-frozen in eppendorfs. After letting the chase proceed for 12.5 minutes, we added glutamine from a 100mM stock (dissolved in water) to a final concentration of 400μM to one flask, or an equal amount of water to the control flask. Samples were extracted using a hot acid-phenol method, with equal volume of synthetic spike-ins added to each RNA extraction reaction. 4tU-containing spike-ins (polyadenylated coding sequences from *B. subtilus* and *C. elegans*) were synthesized *in-vitro* as previously described [48]. RNA was reacted with MTSEA-biotin to conjugate biotin to the 4-thiouracil-containing RNA, then purified using streptavidin beads. Fractionated RNA was depleted of rRNA using a RiboZero kit. RNA samples were converted into Illumina sequencing libraries using a strand-specific (UNG) protocol, ligating adapters that contain UMI’s [90]. Libraries were pooled and sequenced by the NYU Genomics Core sequencing facility on an Illumina NextSeq. Following base-calling and sample demultiplexing by NYU GenCore, the sequencing reads were trimmed using cutadapt [91] aligned using tophat2 [92] to a reference genome that included the yeast reference genome (assembly R64-2-1) and spike-ins, filtered for mapping-quality and length using samtools [93], deduplicated with umi_tools [94] and feature counting was performed using htseq-count [95]. Feature counts for yeast mRNAs were normalized to synthetic spike-ins, using the fitted values from a log-linear model of spike-in abundance increase (see Results, and S1 Appendix). The rate of mRNA degradation and changes in this rate was quantified assuming an exponential model (S1 Appendix) and fit as a linear model to log transformed data. Significant changes in mRNA degradation rates were defined using a FDR [96] less than 0.01 and a doubling in degradation rate (based on modeling detailed in S1 Appendix).

### Label-chase RNA sequencing cis element analysis

To detect if *de novo* or known *cis* elements were associated with destabilization upon a nitrogen upshift, we used DECOD [97], FIRE [98], TEISER [99], and the #ATS pipeline [100]. We also scanned for association with RBP binding sites from the CISBP-RNA database [101] using AME from the MEME suite [102]. Final plots in the supplement were made using motif scans with GRanges [103]. Analysis was done using coding sequence and four different definitions of untranslated regions (200bp upstream of the start codon or downstream of the stop codon, the largest detected isoform in TIF-seq data [104], or the most distal detected gPAR-CliP sites in exponential-phase or nitrogen-limited growth [105]).

### Barseq after FACS after mRNA FISH (BFF)

The methods and analysis are detailed in S2 Appendix, including motivation, protocols, and manufacturer information.

An aliquot of the prototrophic deletion collection [62] was thawed and diluted, with approximately 78 million cells added to 500mL of nitrogen-limited (proline) media in a 1L baffled flask. The culture was shaken at 30°C overnight, then split into three flasks (A, B, and C). After three hours (at mid-exponential) we collected samples of 30mL culture filtered onto a 25mm filter and flash-frozen in an eppendorf in liquid nitrogen. We sampled in steady-state growth (pre-upshift) and 10.5 minutes after adding 400μM glutamine (post-upshift). Samples of the pool were fixed with formaldehyde (4% PFA diluted in PBS from 10mL aliquot, buffered, 2 hours room-temperature) and digested with lyticase (in BufferB with VRC 37° 1 hour), [106], and permeabilized with ethanol at 4° overnight. Samples were processed with a Affymetrix Quantigene Flow RNA kit (product code 15710) designed to target *GAP1* mRNA and labelled with Alexa 647. The hybridization was performed using a modified version of the manufacturer’s protocol (Appendix S2 Appendix, including a DAPI staining step. Samples were sonicated, then run through a BD FACSAria II. Cells were gated for singlets and DAPI content (estimated 1N or more), then sorted based on signal detected with a 660/20nm filter with a 633nm laser activation into four gates within each timepoint. Cells were sorted using PBS sheath fluid at room-temperature, into poly-propylene FACS tubes, then stored at -20°C. For each gate, cells were collected via centrifugation and genomic DNA extracted by NaCl reverse-crosslinking at 65°C, inspired by [69], with subsequent proteinase K and RNase A digestions. Genomic DNA was split into three reactions and assayed using a modified barseq protocol (S2 Appendix). See the supplementary write-up S2 Appendix for detailed protocols and rationale. Barseq libraries were submitted to the NYU Genomics Core for sequencing using a 1x75bp run on a Illumina NextSeq.

### Analysis of BFF sequencing results

We devised a pipeline to quantify barcodes using the UMI sequence incorporated in the first round of amplicon priming, and benchmarked on *in silico* simulated datasets (S2 Appendix). Briefly, raw FASTQ files are processed with SLAPCHOP (https://github.com/darachm/slapchop) which uses pair-wise alignment [107] to filter, extract UMIs from variable positions, and extract barcodes. We demultiplexed using a perl script, and aligned strain barcodes to a reference barcode index [63] using bwa mem [108]. Barcodes were counted and then we used UMIs with the label-collision correction of [71] to quantify the proportion of each mutant in the sample. These relative counts and the FACS data (the sorted events per bin) were used to estimate the distribution of each mutant across the four gates in each timepoint. We filtered for strains detected in at least three bins, and fit a log-normal distribution using mle in R [109]. The mean of this distribution was used as the expression value of *GAP1* mRNA in plots and GSEA analysis using clusterProfiler [110].

## Supporting information

S1 Appendix Supplementary file with experimental rationale, details, and protocol for the label-chase experiment. (PDF)

S2 Appendix Supplementary file with experimental rationale, details, and protocol for the BFF experiment. (PDF)

S1 Table Principal components loadings of the microarray samples reprocessed from Airoldi et. al. 2016. (CSV)

S2 Table Gene set enrichment analysis of loadings on principal components one and two. (CSV)

S3 Table Raw counts of labeled mRNA quantified by RNAseq in label-chase experiment. (CSV)

S4 Table Filtered label-chase RNAseq data for modeling, normalized directly within sample. (CSV)

S5 Table Filtered label-chase RNAseq data for modeling, normalized by modeling across samples. (CSV)

S6 Table Degradation rate modeling results, from data normalized across samples. (CSV)

S7 Table Enriched GO and KEGG terms within the set of mRNA destabilized upon a nitrogen upshift, across sample normalization. (CSV)

S8 Table Raw counts of events in each bin in the BFF experiment, and gate settings for the observations. (CSV)

S9 Table Parameters of all 3230 models fit to filtered data. (CSV)

S10 Table Gene-set enrichment analysis results using GAP1 estimates. (CSV)

S11 Table Strains used in this study. (CSV)

S12 Table Primers used in this study. All primers were synthesized by Integrated DNA Technologies (IDT). Barseq multiplexing barcode sequences and index numbers available in the file data/dme209/sampleBarcodesRobinson2014.txt within the data zip archive (Availability of data and analysis scripts).
(CSV)

S1 Fig The long-term transcriptome dynamics of a glutamine upshift. Principal components analysis (SVD) of microarray data from [25]. Colored points are from steady-state chemostats grown in limitation for various nitrogen sources, at different growth rates. Time-series experiments are show in grey points, connected by lines, and line-type is the type of upshift (in batch or in chemostat).
(TIFF)

S2 Fig The timing of changes in the component loadings of microarray samples. The changes in loadings in the first two principal components of microarray data analyzed from [25], as in Fig 1C, for chemostat upshift experiments only. In the chemostat, addition of 400μM glutamine has a more pronounced response than 40μM glutamine addition, but all have a sharp response in short timescale.
(TIFF)

S3 Fig Most of the variation is explained in the first two principal components. **A)** From the PCA analysis used in Fig 1C, the variance explained is plotted for the principal components, showing a steep decrease in explained variance after the first two components. **B)** The third and fourth components explain a small amount of the variance and are not readily interpretable with respect to the transcriptome changes in response to the changing growth rates in either steady-state or dynamic conditions.
(TIFF)

S4 Fig Comparison between rates of mRNA abundance changes and mRNA stability. Comparisons of measured mRNA degradation from this study with mRNA abundance change rates from [25]. Pre-upshift degradation rates (top) don’t explain the abundance change. The degradation rate changes (middle, difference between pre and post upshift) and the post-upshift rates (bottom) are anti-correlated with the abundance changes.
(TIFF)

S5 Fig Many of the destabilized mRNA are members of the ESR-up regulon. Comparisons of degradation rates from this study with mRNA abundance change rates from [25]. Destabilized transcripts are colored based on their membership in the ESR gene set, as described in the supplement of [86]. Many of the destabilized set are “ESR-up” genes, as they are increase in expression in response to stresses.
(TIFF)

S6 Fig Scatter plot of significantly destabilized transcripts. For each transcript the x-axis is the rate of degradation rate post-upshift and the y-axis is the mRNA abundance change rate [25] after the upshift. The dashed line is a 1:1 line of equality.
(TIFF)

S7 Fig Examples of individual mRNA with counteracting changes in mRNA synthesis and degradation. For several transcripts we found an increased rate of degradation post-upshift (red) compared to before the upshift (blue) but minimal changes in abundance (black). Each dataset is normalized to intersect at the same t = 0 intercept.
(TIFF)

S8 Fig The destabilized set of transcripts is longer and has a higher frequency of optimal codons than the rest of the transcriptome. Comparisons of destabilized mRNAs with the rest of the transcriptome. **A)** Destabilized transcripts tend to have longer CDS lengths (p-value < 2 × 10^−5^ by Wilcoxon rank sum test). **B)** On average, the destabilized transcripts have more optimal codons than the rest of the transcriptome (p-value < 2 × 10^−8^ Wilcoxon rank sum test). The fraction of optimal codons per feature was obtained from the supplement of [58] using definitions from [111].
(TIFF)

S9 Fig Enrichment of Hrp1p motif in 5’ UTRs of destabilized transcripts. Sequences were analyzed for RBP binding motif enrichment using the AME program in MEME and significant hits confirmed using a logistic model predicting destabilization based on motif content per sequence length. Hrp1p is significantly (p < 0.0001) enriched in the 5’ UTRs of destabilized transcripts. Motif matches were counted using the GRanges package for the 5’ UTRs, 3’ UTRs, and coding sequence of transcripts using the largest isoforms detected in [104].
(TIFF)

S10 Fig GAP1 delete or omission of the targeting probe results in no GAP1 FISH signal. Wild-type or *GAP1*Δ cells were grown in proline-media. As seen in the positive control there is heterogeneity in the signal.
(TIFF)

S11 Fig Principal components analysis of the abundance estimates using BFF. Each color is a type of sample, from low to high gates (with black denoting the input samples before sort). Technical replicates are connected by dashed lines, biological replicates are denoted with letters A B or C. The first two principal components show the separation of gates by signal intensity and reflects that the lower gates on the upshifted samples were very close (blue and red samples on far right panel), within the distribution of the negative population. This is consistent with their tight sampling of the “GAP1-off” population, as seen in Fig 4A.
(TIFF)

S12 Fig Rarefaction curve of UMI saturation. The solid-line curve denotes the theoretical expectation of total observations per UMI in a sample (x-axis) and the number of unique UMIs (y-axis). This curve shows how UMI-collisions are expected to depress the number of unique UMIs. Each point is from real data, with these two numbers tabulated for each combination of a sample and strain barcode. We see that these largely follow the curve of saturation of UMI-collisions, but that it falls well below the expectation of independent UMI-collision, thus we believe that there is an additional contribution of PCR-amplification noise (PCR duplicates).
(TIFF)

S13 Fig Knock-out mutants of negative regulators of gluconeogenesis are associated with higher GAP1 mRNA expression after the upshift. Knock-out mutants of negative regulators of gluconeogenesis are associated with higher estimated *GAP1* mean after the upshift, by GSEA analysis of GO-terms (p-value < 0.05).
(TIFF)

S14 Fig Knock-out mutants of genes involved in sulfate assimilation are associated with higher estimated GAP1 mRNA expression mean before the upshift. Knock-out mutants of involved in sulfate assimilation are associated with higher estimated *GAP1* mean before the upshift, by GSEA analysis of GO-terms (p-value < 0.05).
(TIFF)

S15 Fig The relationship between mean GAP1 mean expression before the shift and after the upshift. Scatter plot of the estimated means, with marginal histograms along top and right. Red vertical line on top histogram is a cut-off of *GAP1* mRNA induction for this analysis, and is the mean of the fit to wild-type minus one standard deviation of that distribution. The red linear regression line is fit to all points above this threshold, in which expression was detected before the upshift.
(TIFF)

S16 Fig tco89Δ and xrn1Δ show defects in GAP1 mRNA regulation in the BFF assay. *xrn1*Δ mutant (left) is lowly abundant in the library and is only observed in the highest bin of *GAP1* signal, consistent with the role of Xrn1p as a global exonuclease. *tco89*Δ is the only detected member that would abrogate TORC1 activity. This mutant (right) has elevated *GAP1* mRNA before and after the upshift, consistent with the role of TORC1 in repressing the NCR regulon.
(TIFF)

S17 Fig Two independent deletions of the GAP1 5’ UTR show the same phenotype. A deletion of 152bp 5’ of the start codon was also generated. We tested *GAP1* dynamics in this strain as well, and found that it shares the same phenotype as a 100bp 5’ UTR deletion. Methods are the same as in Fig 5E, both 5’ UTR deletes are slowed in clearance, ANCOVA p < 0.05.
(TIFF)

S18 Fig Processing-body dynamics are not associated with the nitrogen upshift. A strain containing a copy of Dcp2p-GFP expressed from a plasmid was grown in conditions of exponential phase in YPD or 10 minutes of starvation in water (first row) to confirm detection of processing-body foci using Dcp2-GFP. We do not see either formation or dissolution of Dcp2-GFP foci during the nitrogen upshift (bottom row).
(TIFF)
