# Supplementary material for: Systematic identification of factors mediating accelerated mRNA degradation in response to changes in environmental nitrogen
Source: PLoS Genet. 2018 May 21;14(5):e1007406. doi: 10.1371/journal.pgen.1007406 (PMC5983874; doi:10.1371/journal.pgen.1007406)
Supplement: S1 Appendix — (PDF) [file pgen.1007406.s001.pdf]

Appendix 1 - supplementary protocol and writeup

“Systematic identification of factors mediating accelerated mRNA degradation in response to changes in environmental nitrogen.”

Darach Miller, Nathan Brandt, David Gresham

2018

Contents

4tU label-chase RNAseq . . . **1**
Experimental methods . . . 1
Synthetic RNA spike-in generation . . . 2
Culturing and sampling . . . 2
RNA Extraction . . . 4
Biotinlyation and fractionation . . . 4
rRNA depletion . . . 5
Preparing sequencing libraries . . . 5
Analysis . . . 8
Quantifying sequencing reads . . . 8
Normalization of counts into signal for modeling . . . 8
Model of transcript dynamics as a function of degradation rate and labeling parameters 11
Estimating possible effects of synthesis changes on labeled abundance . . . 12
Cis element analysis . . . 14

4tU label-chase RNAseq

Experimental methods

4tU labelling methods were similar to those described for RATEseq[1], but with an experimental design similar to Munchel et. al.[2]. Below details the benchwork methods up through submission to a DNA sequencing core facility.

[1] Neymotin, Athansidou, Gresham *RNA* 2014
[2] Munchel et. al. 2011 Molecular Biology of the Cell.

Synthetic RNA spike-in generation

Poly-adenylated RNA molecules were synthesized *in vitro* using a Promega Riboprobe SP6 kit (P1420), with 4-thiouridine, to serve as spike-in calibrators for RNAseq normalization across samples.

In-vitro spike-ins were generated as previously described[3]. Four plasmids containing sequence cloned from *B. subtilus* and *C. elegans* with a SP6 promoter and poly-adenosine sequence were used. For each transcription reaction, using reagents from the Promega Riboprobe kit (P1420), approximately 625ng of linearized template was combined in 20μL reaction volume with 4μL 5x transcription-optimized reaction buffer, 2μL 100mM DTT, 0.75μL RNasin, 1μL each of 10mM rATP, rUTP, rCTP, rGTP, 2μL of 10mM 4-thio-rUTP (Jena Biosciences #NU-1156S), and 1μL SP6 RNA polymerase. These reactions were incubated 2 hours in a 37°C waterbath, then 1μL of RQ1 DNAse was added and tubes returned to incubation at 37°C for 15 minutes. To each 20μL reaction, 40μL of Ampure XP beads (APG3881) were added and mixed. These were incubated at room temperature for 5 minutes, then beads were collected on a magnetic rack. The supernatant was removed and beads washed with 80% ethanol for 30 seconds, twice. Beads were dried 10 minutes at room temperature, with open lids. The beads were resuspended in 20μl of hyclone water, then pulled down and supernatant collected and quantified using the Qubit HS RNA assay (Invitrogen Q32855). Equivalent mass amounts of spike-ins were pooled to create a 8ng/μL stock containing all four 4-thiouridine-labeled spike-ins.

We also prepared total 4-thiouracil labeled *E. coli* RNA to use as another spike-in. We grew strain MG1655 (a gift of Edo Kussell) overnight in 5mL of LB with 20μM of 4-thiouracil. We pelleted 410μL of the culture and resuspended in 5mL of LB with 20μM 4-thiouracil and let it grow at 37°C for 2.5 hours. This culture was spun to pellets, and froze at -80°C. To extract, the pellet was resuspended in 400μL of 1% SDS + 100mM NaCl + 8mM EDTA, then put on a 100°C heatblock. This was vortexed every minute for 5 minutes, then 800μL of acid-phenol:chloroform (pre-warmed to 65°C) was added. This was vortexed and incubated at 65°C for 10 minutes, then spun at max speed 1 minute. The supernatant was taken to a new tube and we added 300μL acid-phenol and 300μL chloroform. This was extracted again with acid-phenol, then aqueous fraction was extracted with chloroform in a phase-lock gel tube (5Prime #2302830), then ethanol precipitated. The final solution was quantified using qubit and diluted to a 5ng/μL solution of thiolated total *E. coli* RNA.

Culturing and sampling

FY4 was grown in nitrogen-limitation conditions overnight with a mixture of 50μM:50μM of 4-thiouracil:uracil. This culture was split, then 4mM uracil was added to chase the 4-thiouracil label with a 41-fold excess of uracil. Samples were taken by filtration and flash-freezing.

We isolated a single colony of wild-type haploid protrophic (FY4) yeast in 50mL proline-limited minimal media ("NLimPro", with 800μM L-proline, as described in the "Media and upshifts of media" section) supplemented with 50uM uracil ("NLimProUra"). This culture was back diluted from mid-exponential phase growth to a density of 1.18×10^5 cells per mL in 1L of NLimPro, at which point 125μL 400mM uracil (vendor) and 250μL 200mM 4-thiouracil (vendor), both dissolved in DMSO,

[3] Neymotin, Athansidou, Gresham *RNA* 2014

were added to reach 50μM of both 4-thiouracil and uracil. This culture was grown for 26 hours to label all RNA. The culture was split into two 450ml cultures 5 hours before the label chase began. During exponential phase ($\sim 5 \times 10^6$ cells per mL), uracil from a 400mM DMSO stock was added to a final concentration of 4mM (41-fold excess) to chase the label. 30mL samples from the culture were filtered onto 25 millimeter nylon filters, then flash-frozen in eppendorf tubes in liquid nitrogen within a minute of removal from culture. Sampling time is recorded as the time of flash-freezing. After letting the chase proceed, we added glutamine from 100mM stock (dissolved in water) to a final concentration of 400μM to one flask, or an equal volume of water to the control flask.

Action	Treatment series	Minutes after uracil chase
+4.5ml 400mM uracil	water (mock upshift)	0
Took sample 1	water	3.85
Took sample 2	water	6.02
Took sample 3	water	7.92
Took sample 4	water	9.90
Took sample 5	water	11.8
Added 1.22ml hyclone water	water	13.0
Took sample 6	water	15.1
Took sample 7	water	17.0
Took sample 8	water	18.8
Took sample 9	water	20.8
Took sample 10	water	22.9
Took sample 11	water	26.1
Took sample 12	water	50.5
+4.5ml 400mM uracil	glutamine (nitrogen-upshift)	0
Took sample 1	glutamine	3.30
Took sample 2	glutamine	5.32
Took sample 3	glutamine	7.65
Took sample 4	glutamine	9.47
Took sample 5	glutamine	11.3
+1.22ml 100mM glutamine	glutamine	12.5
Took sample 6	glutamine	14.4
Took sample 7	glutamine	16.4
Took sample 8	glutamine	18.2
Took sample 9	glutamine	20.0
Took sample 10	glutamine	23.8
Took sample 11	glutamine	28.8
Took sample 12	glutamine	49.1

Times denote addition of reagent or time of flash-freezing the tube containing the filter. All tubes were stored at -80°C.

RNA Extraction

Since equal volume (30mL) of culture was taken for each sample, an equal volume of synthetic spike-ins was added to each RNA extraction reaction (hot acid-phenol method).

Total RNA was extracted by addition of 400μL of fresh TES[4] quickly followed by 400μL acid phenol (Fisher). Each tube was vortexed vigorously and put at 65°C on a heatblock for 5 minutes. Each tube was lightly spun to pull solution down from the lid, then 5μL of 8ng/μL *in-vitro* synthetic spike-ins (above) and 5μL of 5ng/μL thiolated "ecoli" total RNA (above) were added to each sample. Samples were then vortexed very well, incubated for 20 minutes at 65°C, vortexed vigorously, incubated for 20 minutes at 65°C, vortexed vigorously, and incubated for 20 minutes at 65°C. All tubes were placed on ice 5 minutes, then spun at maximum speed in a room-temperature centrifuge 5 minutes. The top phase was aspirated to a new eppendorf, and 400μL of 50:50 acid-phenol:chloroform solution was added. Tubes were vigorously vortexed, then spun 1 minutes full speed. The aqueous phase was carefully aspirated to a prespun phase-lock gel tube, and 400μL chloroform was added and mixed by inversion. These were spun 5 minutes 15000rcf room-temperature. The aqueous phase was aspirated and added to new tubes with a pre-mixed 2μL gylcogen and 35μL 3M NaAcetate. 875μL 100% ethanol was added, and samples were put on ice 40 minutes. Tubes were spun at 15 minutes maximum speed at 4°C. The supernatant was aspirated, and pellet washed once with 500μL 70% ethanol. This was spun at max speed and aspirated twice, then dried for 10 minutes at room temperature with open lids. Pellet was re-suspended in 50μL hyclone water. The extraction yielded at least 3.3 μg of RNA per 10^7 cells.

Biotinlyation and fractionation

The total RNA (yeast and spike-ins, mixed) was reacted with MTSEA-biotin to conjugate biotin to the 4-thiouracil-containing RNA, then purified. The biotin-conjugated RNA was purified using streptavidin beads.

To each RNA sample of 48μL, we added a master mix of 149μL hyclone + 2.5μL 1M HEPES + 0.5μL 0.5M EDTA. Samples were vortexed and spun, then 50μL of MTSEA-Biotin (biotin-XX, Biotium #90066) 1mg/10ml stock prepared in DMF was added to sample, and mixed well with pipette until visibly mixed. Samples were incubated in the dark at room temperature for 2 hours, then 250μL 24:1 chloroform isoamyl alcohol was added. Samples were vigorously vortexed in multiple axes, then pipetted on top of a pre-spun phase-lock gel tube (5Prime #2302830). These were spun 5 minutes at 15000 rcf room temperature, then top layer aspirated on top onto 25μL 3M Na Acetate + 2μL glycogen (Thermo R0561), and 625μL 100% ethanol was added. These were incubated on ice for 30 minutes, then spun 15 minutes maximum speed 4°C. Pellets were washed with 70% ethanol, centrifuged maximum speed room temperature and aspirated twice, then dried 10 minutes room temperature with open lids.

Biotinylated total RNA was fractionated with streptavidin bead pulldown. 200μL of streptavidin beads (NEB S1420S) were put into new 1.5mL eppendorf tubes. Beads were pulled down with a

[4] 10mM Tris (~7.5), 10mM EDTA, 0.5% SDS

magnetic rack, and washed once with 200μL bead buffer[5] with vortexing. This was pulled down and aspirated again. 150μL of bead buffer was mixed with the thawed total RNA sample, then mixed with the beads by pipette. This mixture of RNA sample and beads was vortexed 5 minutes room temperature, then spun and lightly vortexed, then incubated 15 minutes room temperature on bench. This was pulled down, buffer was aspirated, then 100μL of bead buffer was added and vortexed to resuspend. This mixture was incubated 5 minutes, spun, pulled down, aspirated to eppendorfs. 100μL bead buffer was added, vortexed to resuspend, let sit 1 minutes, then spun, pulled down, and aspirated to waste. Beads were resuspended in 65°C bead buffer, 65°C 1 minutes, then pulled down ~1 minutes, aspirated to waste, and washed again with room temperature bead buffer. Beads were then resuspended in 5% beta-mercaptoethanol, 20μL, and incubated room temperature 10 minutes, then pulled down and supernatant aspirated to new eppendorf. Beads were resuspended in another 20μL of 5% beta-mercaptoethanol at 65°C for 10 minutes, pulled down and put in that same eppendorf for precipitation. 4μL of 3M sodium acetate and 2μL glycogen was added, then 100μL 100% etOH. This was chilled 1 hour, spun 15 minutes 4°C maximum, supernatant aspirated to waste, pellet washed with 70% etOH, then spun twice with aspiration of supernatant to waste. The pellet was dried 10 minutes, then resuspended 10μL hyclone.

rRNA depletion

Fractionated RNA was depleted of rRNA using the RiboZero kit (Illumina RZY1324) according to manufacturer instructions, except that the input we used 2μg input RNA with half-reactions (ie half of every reagent). Final RNA was ethanol precipitated, as above. Agilent Tapestation measurements of the RNA size histograms confirmed that virtually all of the rRNA was removed.

Preparing sequencing libraries

RNA samples were converted into Illumina sequencing libraries using a strand-specific (UNG) protocol.

For 1st strand cDNA synthesis, we combined 6.4μL of fractionated and ribo-depleted RNA with 7.1μL of the following master-mix in PCR tubes:

- 1.5μL 10x RT buffer (Invitrogen 53032)
- 0.8μL 50ng/ul hexamers (Invitrogen 51709)
- 1μL 10mM dNTPs (Invitrogen Y02256)
- 1.3μL 0.1M DTT (Invitrogen Y00122)
- 2.5μL 25mM MgCl2 (Invitrogen Y02222)

These reactions were incubated in a PCR machine (NYXtechnik A6) at:

- 98°C for 1 minute
- 70°C for 5 minutes
- 15°C held

[5] 1M NaCl, 10mM EDTA, 100mM Tris pH 7.4

We added to each reaction 0.9μL of a mixture composed of 8μL of RNAseOUT (Invitrogen 51535) + 8μL freshly diluted 1x actinomycin (Sigma A1410-2MG) 125 ng/μL solution in etOH, + 8μL SuperScriptIII (Invitrogen 18080-051) This was ~21μL instead of the naive expectation of 24μl, due to the mixture of ethanol and water solvents. The procedure continued:

- 25°C for 10 minutes
- 42°C for 45 minutes
- 50°C for 25 minutes
- 75°C for 15 minutes

These were brought to room temperature, then diluted and transferred to a new low-bind tube using 85μL hyclone, then 10μL sodium acetate 3M, 2μL 5mg/ml glycogen, and 225μL ethanol was added and samples put into -20°C overnight. These were precipitated and spun in a cold (4°C) centrifuge, washed with 70% ethanol, then dried and resuspended in 56μL hyclone.

To make double-stranded cDNA, 55μL of each first-strand synthesized cDNA from above were put in PCR tubes. We added a 4μL of a mixture composed of:

- 1μL 10xRT buffer (Invitrogen 53032)
- 1μL 0.1M DTT (Invitrogen Y00122)
- 2μL 25mM MgCl_2 (Invitrogen Y02222)

These reactions were held on ice, then we added 20μL of a mixture composed of:

- 15μL 5x SS buffer (Invitrogen 10812-014)
- 2μL 10mM dA/G/C/U TP mix (Promega U1335)
- 0.5μL ecoli DNA ligase (Invitrogen 18052-019)
- 2μL DNA Pol I (Invitrogen 18010-017)
- 0.5μL RNAseH (Invitrogen 18021014)

These reactions were mixed with pipette, iced, and moved to a 16°C heatblock for 2 hours. The reactions were cleaned up by purifying on MinElute columns (Qiagen 28004) and eluted twice with the same 18μL of hyclone water.

This double-stranded cDNA was end-repaired, using 16μL of the purified product of the second-strand (previous)reaction. We added to each sample 7.75μL of a mixture composed of:

- 3.5μL hyclone water
- 2.5μL 10x T4 ligase buffer with ATP (NEB B0202S)
- 0.5μL dNTPs 1.25μL T4 DNA polymerase (NEB M0203S)
- 1.25μL T4 PNK (NEB M0201S)

This was incubated at 20°C for 30 minutes, then on ice for ~15 min, then purified with MinElute columns and eluted with 17.5μL EB buffer from the Qiagen kit and stored at -20°C.

This cDNA was A-tailed by master mixing

- 0.7μL of 100mM dATP (Promega U1335)
- 69.3μL hyclone
- 31.5μL NEB Buffer 2 (NEB B7000S)
- 21μL Klenow (exo-) (NEB M0212S)

Put 9μL of master mix in tubes, then added 16μL of purified end-repaired product from above. Mixed and incubated 37°C 30 minutes on PCR machine. At stop, added 5μL sodium acetate and cleaned up with MinElute, eluting with 12μL EB and storing on ice for two hours.

TrUMIseq adapters[6] (similar to TruSeq, but with UMIs in the index barcode position) were added by ligation. These adapters were annealed into the Y-adapter configuration, then diluted to 0.1μM from stocks. 12.5μL of 2x Quick ligase buffer (NEB M2200S) was put in a PCR tube, then 10.5μL A-tailed dsDNA sample was added and mixed with pipette. 0.5μL of the 0.1uM solution of adapters was added, then 1.5μL of the quick ligase (NEB M2200S). These were incubated at 22°C in a PCR machine for 15 minutes, then put on ice and immediately diluted with 75μL hyclone water. We added 100μL Ampure XP beads to bind the product for 15 minutes at RT. The supernatant was discarded and beads washed twice with 80% ethanol. After drying, the products were eluted in 20μL of 0.25x quick ligase buffer and cleaned up with Ampure XP beads again, using a 50:50 bead:reaction mix. Once dried, the products were eluted with 20μL hyclone water.

To amplify libraries and select the strand-specificity, we prepared a master-mix:

- 10μL 5x HF buffer (NEB M0530S)
- 1μL 10uM DGO366 (see primer table)
- 1μL 10uM DGO367 (see primer table)
- 1μL 10mM dNTPs (Invitrogen 18080-051)
- 1μL UNG (Thermo EN0361)

To this, half the adapter-purified products from above and hyclone water were added to a volume of 49.5μL. These reactions were incubated in a PCR machine:

- 15 minutes at 37°C
- 10 minutes at 90°C
- hold at 60°C, while 0.5μL of Phusion polymerase (NEB M0530S) was added.
- 98°C for 2 minutes
- 18 repetitions:
  - 98°C 30 seconds
  - 60°C 30 seconds
  - 72°C 15 seconds
- 72°C 2 minutes
- hold at 4°C

These reactions were cleaned up using a MinElute column, then diluted and concentration estimated using qPCR on a Roche 480 (using KAPA Library Quant Kit Illumina REF 07960281001), and submitted as a 1nM pool to the NYU GenCore system for sequencing on a NextSeq using the 75bp format in High-Output mode.

[6] Hong and Gresham 2017 BioTechniques

Analysis

Quantifying sequencing reads

Following base-calling and demultiplexing by NYU GenCore, the sequencing reads were quantified using the following pipeline:

1. Raw reads were trimmed using `cutadapt`[7]
2. Trimmed reads were aligned using `tophat2`[8] to a reference genome that included the yeast reference genome (assembly R64), the Ecoli genome (assembly GCF_000005845.2), and the four synthetic in-vitro transcribed spike-ins (termed BES and available in the `data.zip` archive). This was done with parameters optimized against *in silico* data generated by Flux Simulator[9] from this reference genome, in replicates.
3. Reads with mapping quality above 20 and with at least 50 matched bases were processed with `umi_tools`[10] in "dir" mode to de-duplicate possible PCR duplicates.
4. The demultiplexed `.bam` file was processed with the `htseq-count`[11] script to generate counts files per gene feature (according to the GFF file in the `data/BES` directory).

Normalization of counts into signal for modeling

Feature counts for yeast mRNAs were normalized to the synthetic spike-ins. The simplest normalization is to divide each feature counts by the sum of the counts of all the spike-ins. However, several samples had poor quantification of the spike-in which required us to remove outlier measurements to prevent systematically noisy data from disrupting the quantification. We also smoothed the spike-in signal before normalization by modeling the spike-in fraction over the duration of the chase as a log-linear increase.

The log of the proportion of counts that are spike-ins increases over the course of our experiment.

[7] https://cutadapt.readthedocs.io/ , https://doi.org/10.14806/ej.17.1.200
[8] http://ccb.jhu.edu/software/tophat/manual.shtml , https://doi.org/10.1186/gb-2013-14-4-r36
[9] http://sammeth.net/confluence/display/SIM/Home , https://doi.org/10.1093/nar/gks666
[10] https://github.com/CGATOxford/UMI-tools , https://doi.org/10.1101/gr.209601.116
[11] http://htseq.readthedocs.io/ , https://doi.org/10.1093/bioinformatics/btu638

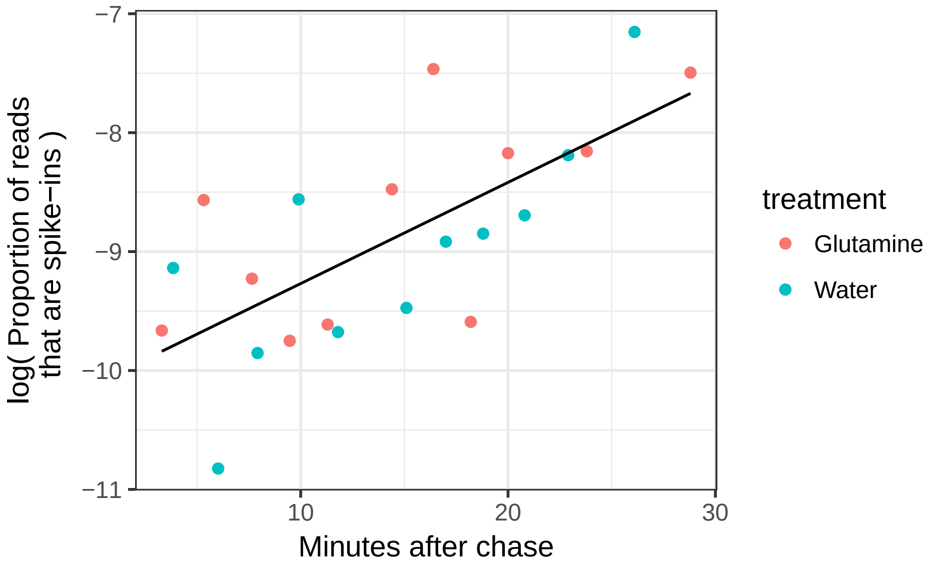

Figure 1: Proportion of counts that are spike-ins increase over time to a new eqilibrium.

We modeled this increase using the `lm` function. Here are the residuals:

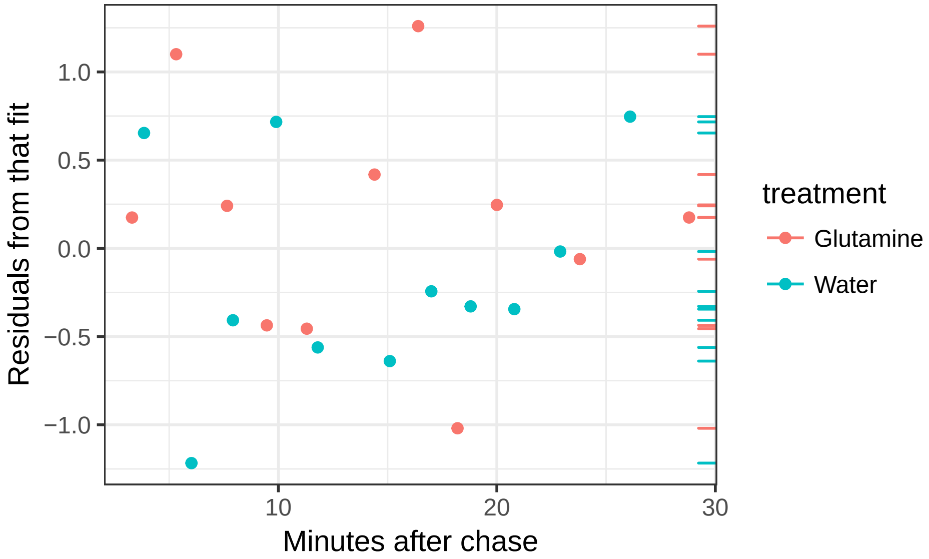

Figure 2: The residuals of the observations from the model of a linear increase of log-proportions across the experiment.

Do the residuals for each treatment change with time differently? We did an ANCOVA (`aov`/`lm`), and found the effect of treatment was associated with a p-value < 0.3013742 and the p-value associated with time estimated as “1”, so it does not appear that the residuals depend on time or treatment.

How do the normalizations compare on a per-gene basis? Figure 3 shows the normalized data for several genes, on the left is the direct, within sample normalization and on the right is this smoothing between samples using a log-linear model.

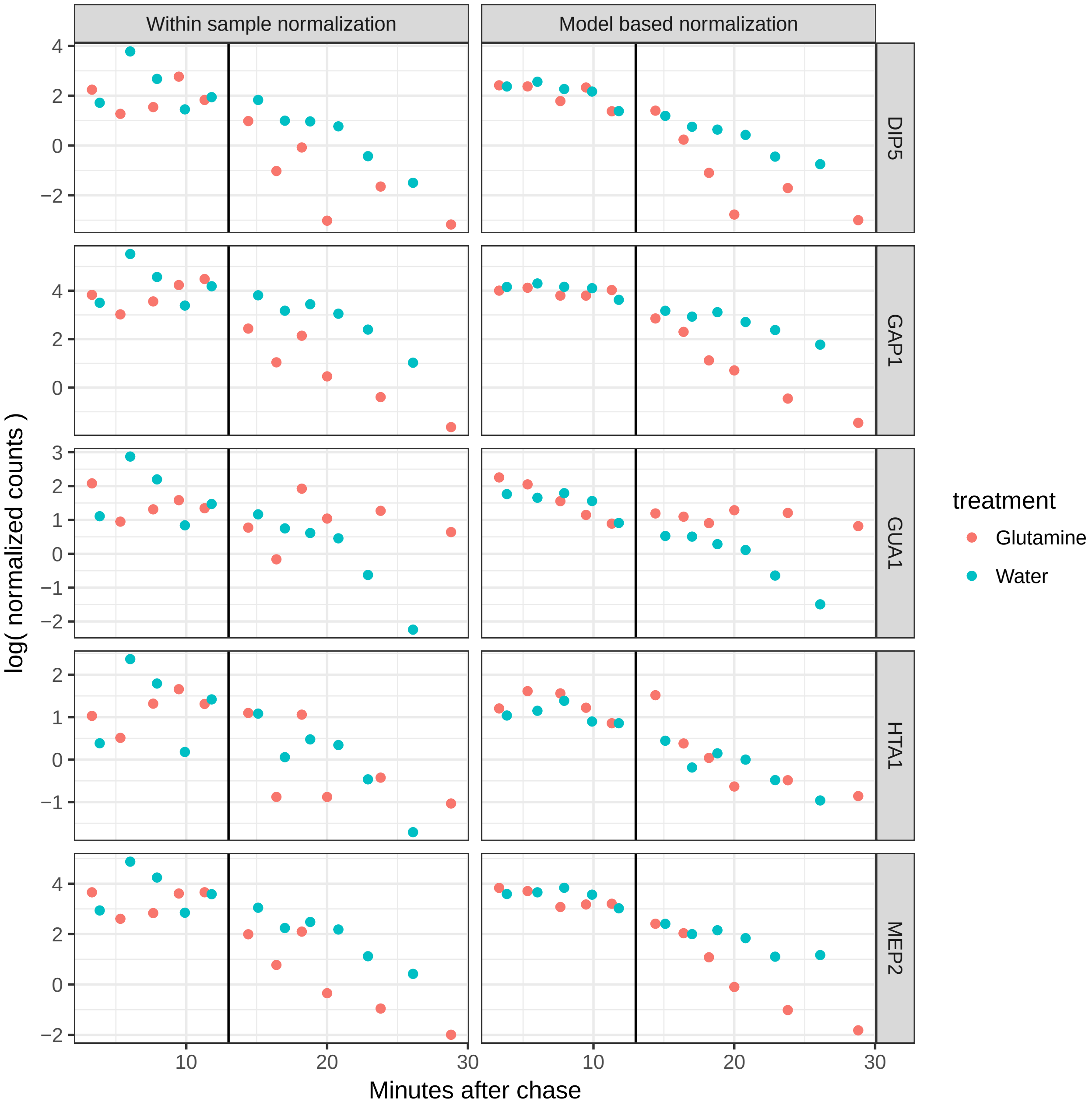

Figure 3: Examples of individual gene signals normalized with both approaches.

We also tried to spike-in labeled ecoli total RNA; however, we found that those counts were low, noisy, and did not behave as expected. We hypothesize that this was due to lower addition of ecoli total RNA than synthetic spike-ins, combined with noise associated with amplifying a random sub-sample of a more complex spike-in pool of total ecoli RNA. Thus, we normalized all yeast mRNA to the synthetic spike-ins previously demonstrated.

Model of transcript dynamics as a function of degradation rate and labeling parameters

Below is our heuristic model of the labeled transcript dynamics in this experiment. We used this to analyze the dataset for expected label-chase dynamics.

m_t is the labeled mRNA at time t. It changes according to the equation:

$$\frac{dm_t}{dt} = Lk_s - k_d m_t$$

where L is the fraction of new mRNA that is labeled and pulled down, k_s is the rate of synthesis, and k_d is the rate of degradation. IMPORTANTLY, k_d does not refer to the dissociation constant in this document, but rather the specific rate of degradation of a transcript. Our experimental design is to change L from an initial fraction of transcripts that are pulled down by a 4tU-incorporation-dependent mechanism of L^o (old) to a new fraction L^n (new). Note that we use the notation as a superscript, so that we can also share that notation with the synthesis rates as k_s^o and degradation rates as k_d^o.

We assume that the culture is at a steady state of synthesis and degradation at a fixed labeling fraction of L^o. From solving the above equation, the signal for a certain mRNA feature we model as reaching an equilibrium of $L^o \frac{k_s^o}{k_d^o}$. We then assume that changes in stability occur rapidly, which is a simplifying assumption but one supported by previous studies of transcript stability changes during shifts (Perez-Ortin et. al. 2013 review), we then expect that m_t should change as a result of changes in the labelling parameter or rates of synthesis or degradation as,

$$m_t = L^o \frac{k_s^o}{k_d^o} e^{-k_d^n t} + L^n \frac{k_s^n}{k_d^n}(1 - e^{-k_d^n t})$$

Nicely, the solution is similar to what we would expect intuitively - extant transcripts decay (left), and nascent transcripts approach the new equilibrium (right). The equilibriums are set by all parameters, but the change between them is dictated by the new degradation rate operative during the transition.

In the case were either L^o or L^n is 0, then the transcript behaves just as one side of the equation. With the label-chase, we are trying to get L^n as low as is possible without perturbing the system being measured by killing the cell.

To analyze this dataset for potential changes in transcript stability, we approximated this by fitting a linear regression model to the normalized signal. We explore the sufficiency of this model later in this document using simulations. This model was fit using the `lm` function in R, with the formula

```
log( NormalizedSignal ) ~ Minutes + Minutes:Treated + 1
```

where "`NormalizedSignal`" is the signal of the gene feature normalized as described in the previous section, "`Minutes` is minutes relative to the glutamine (or water) addition, `Minutes:Treated`" is an additional slope of the observations after glutamine addition, and "`+ 1`" denotes to fit a single intercept for the model. From this fit, we took the p-values associated with the t-statistic of the additional slope fit to the glutamine treated samples, then adjusted the p-values using the `qvalue` package from BioConductor using default settings. We chose to use a FDR cut-off of less than 0.01 for this analysis.

Importantly, this approach estimates both k_o and k_d from the data, by using the mock-treatment dataset.

Estimating possible effects of synthesis changes on labeled abundance

In our experimental design we initially grow the cells in a 50μM:50μM mix of uracil and 4-thiouracil, so we will set as a labeling ratio L^o of 1 for simplicity. We add 4,000 μM uracil to begin the chase, so this is a shift to a L^n of $\frac{50\mu M}{4100\mu M} / \frac{50\mu M}{100\mu M}$, or $\frac{1}{41}$. Since we are not reducing this number to zero, there is still residual labeling incorporated into nascent transcription. Thus, there is a potential that residual label could confound our estimate of degradation rates. This is ainherent tradeoff in a label-chase design, especially since the low RNA content of the cells and low cell density in these nitrogen limited conditions make necessary the use of a more efficient pull-down reagent (MTSEA-biotin). This could be circumvented by comparing abundance and synthesis measurements, but the uracil transporter responding to glutamine in the media makes this technically difficult with 4tU incorporation. Comparing abundance and mRNA synthesis by other means is feasible, but introduces a compounding of errors from both methods. Thus performing one direct assay is preferable for precision.

Therefore, we used simulations to investigate how varying the labelling parameter changes the expected dynamics if we also vary the synthesis parameter. Figure 4 shows a plot of the modeled labeled transcript abundance, with no change in synthesis parameter.

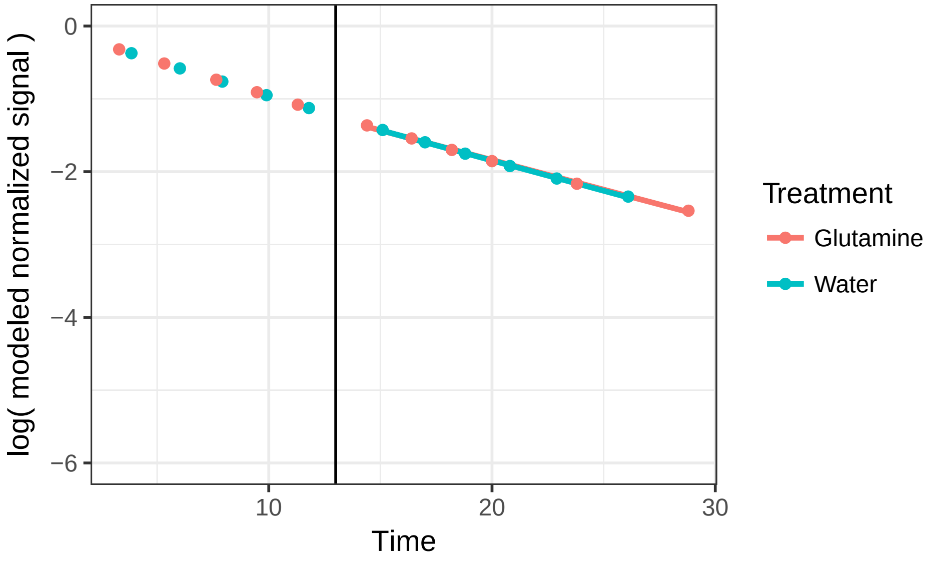

Figure 4: Modeling no changes in the transcript kinetics, simply a change in labeling fraction. K_d of 0.1 .

We are modeling this with a 0.1 k_d. Is this a reasonable rate for modeling here? What is estimated genome wide?

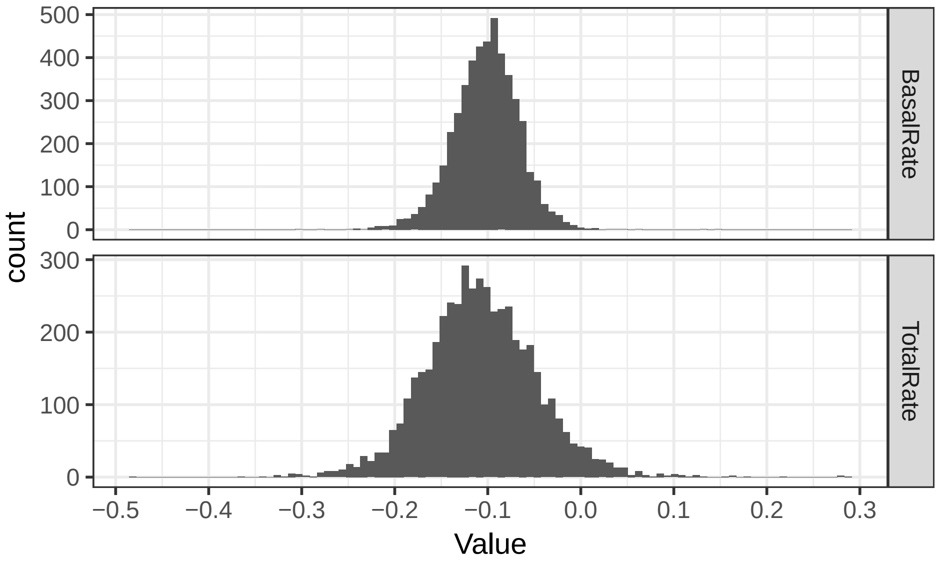

Figure 5: Distribution of parameters from the initial log-linear fits, for the basal (pre-upshift) rate (k_d^o) at top, the change in slopes upon the glutamine upshift in the middle ($k_d^n - k_d^o$), and the post-upshift final rate (addition of the top and middle per gene, k_d^n) on the bottom.

The median observed rate is nicely right around 0.1002 specific degradation per minute.

How does this estimate of change in degradation look if we decrease the k_s? For example, the NCR regulon is expected to be shut-off at the synthesis level quickly upon glutamine addition, so how would that swift repression affect the apparent change in labeled mRNA dynamics?

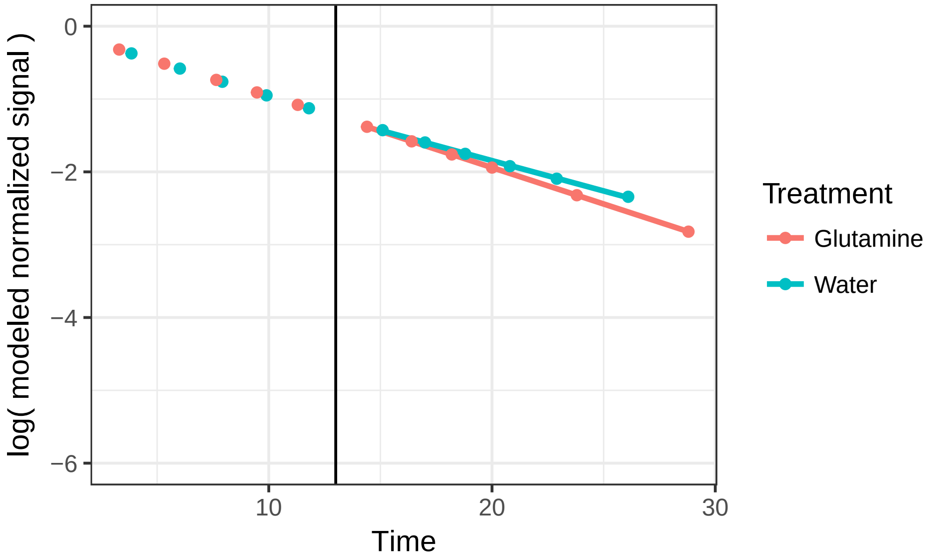

Figure 6: Theoretical data simulated assuming a change in labelling fractions, but with a complete shutoff of synthesis (goes to 0). K_d of 0.1 assumed.

As synthesis reduces to zero, we approach the case where the effect of reduced synthesis on appar-

ent slope change of the labeled RNA is going to be a 13.3% increase in the rate.

What does this mean for our estimates of destabilization? What effect sizes are estimated, and how do they compare to this inflation of 13.3%? Figure 7 shows the distribution of the fold changes in stability:

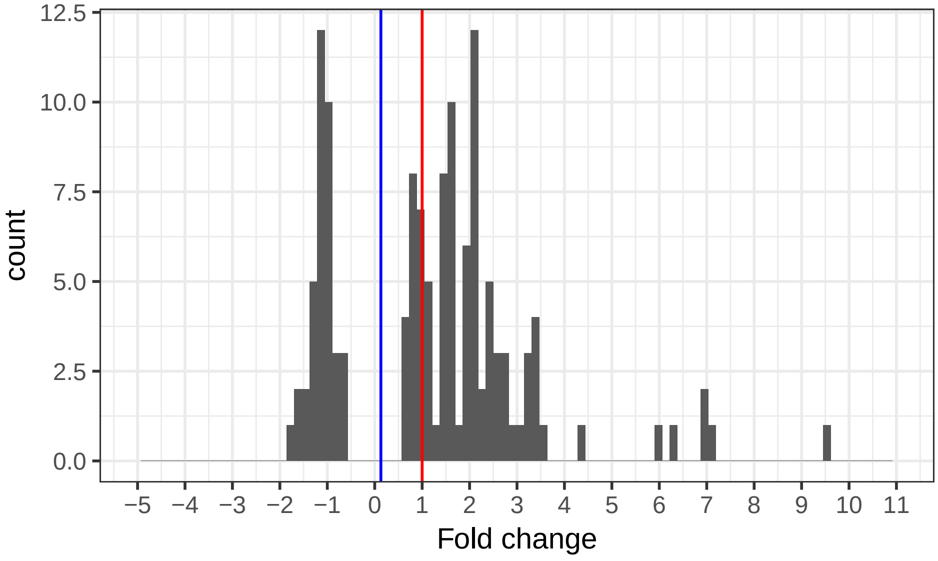

Figure 7: Distribution of fraction of rate change for linear models with signficant (FDR < 0.01) changes in slope of signal change in the experiment.

We see that all of the significant changes are in great excess to that blue line. To be careful, we choose to use a cut off of a 100% increase, a doubling, of apparent degradation rate to call a feature destabilized (right of the red line). Since we cannot place an upper bound on the synthesis rates after a glutamine upshift, we cannot definitively say that the potentially stabilized transcripts (left of 0) are stabilized without additional experiments.

Could these fits just be on the right side of the blue line by chance? Given that the t-statistics for the fits of ones over this line are a median of -5.66, then we're not going to have fits within several standard errors of crossing that threshold by a reasonably expected error.

We conclude that the RNA from 78 gene features appear to be degraded much more quickly than can be reasonably explained by labelling carry-over, and are thus accelerated in degradation upon the nitrogen upshift.

***Cis* element analysis**

We used a variety of bioinformatic methods to detect if *de novo* or known *cis* elements were associated with the phenotype of destabilization upon a glutamine upshift.

For each transcript, we used a GFF file to extract the coding sequence of each annotated mRNA and four different definitions of it's untranslated regions — 200bp upstream of the start codon or downstream of the stop codon, the largest detected isoform in TIF-seq from Pelechano et. al. 2014, or

the most distal detected gPAR-CliP sites in exponential-phase or nitrogen-limited growth in Freeberg and Han et. al. 2013.

To find putative cis-elements, we used DECOD (Huggins et al. 2011), FIRE (Elemento et al. 2007), TEISER (Goodarzi et al. 2012), and the #ATS pipeline (Li et al. 2010). We also scanned for RBP binding sites from CISBP-RNA (Ray et al. 2013) using AME from the MEME suite (McLeay and Bailey 2010). Final plots in the supplement were made using motif scans with GRanges (from BioConductor).
