# Supplementary material for: Systematic identification of factors mediating accelerated mRNA degradation in response to changes in environmental nitrogen
Source: PLoS Genet. 2018 May 21;14(5):e1007406. doi: 10.1371/journal.pgen.1007406 (PMC5983874; doi:10.1371/journal.pgen.1007406)
Supplement: S2 Appendix — (PDF) [file pgen.1007406.s002.pdf]

Appendix 2 - supplementary protocols and writeup

“Systematic identification of factors mediating accelerated mRNA degradation in response to changes in environmental nitrogen.”

Darach Miller, Nathan Brandt, David Gresham

2018

Contents

BFF - BarSeq after FACS after FISH **1**
Experimental methods 1
Culturing and sampling 1
Fixation and permeabilization 2
Hybridization 2
FACS 3
Cell collection and DNA extraction 5
Construction of amplicon sequencing libraries for barcode counting 6
Analysis 12
Designing an analysis pipeline to incorporate UMI information 12
Benchmarking this pipeline 14
Modeling *GAP1* FISH signal per strain in the pool 18

BFF - BarSeq after FACS after FISH

Experimental methods

For BFF, we used mRNA FISH using Quantigene technology to sort cells based on mRNA FISH signal, then prepares barcode sequencing libraries from the collected cells. Below details the benchwork methods up through submission of prepared libraries to a DNA sequencing core facility.

Culturing and sampling

The pool of mutants was grown under nitrogen-limitation, then samples were collected before or after the addition of glutamine.

An aliquot of the prototrophic deletion collection (Vandersluis et. al. 2014) of approximately 1.7 $\times 10^9$ cells was thawed and diluted into ~9mL of NLimPro media, then dilutions were counted on a

hemacytometer to estimate density. From this estimate, approximately 78 million cells were added to 500mL of NLimPro media in a 1L baffled flask. This was shaken at 30°C for about 22 hours, then 140mL of this was split into three baffled culture flasks (250mL size), when the culture was at 2.55 $\times 10^6$ cells per mL. 3 hours later we collected samples. For each sample, 30mL was filtered onto a 25millimeter filter and flash-frozen in an eppendorf dropped into liquid nitrogen. We took two samples at steady-state, then added 320μL 100mM L-glutamine, then took another two samples at 8 minutes and 10 minutes. For the samples reported in this paper, the post-upshift time of freeze-fixation was at 10 minutes and 38 seconds for replicate A, 10 minutes and 12 seconds for replicate B, and 10 minutes and 17 seconds for replicate C. All samples (filters in eppendorfs) were stored at -80°C until processing.

Fixation and permeabilization

Each sample was processed to formaldehyde fix the cells and digest cell walls with lyticase and permeabilize the cells with ethanol.

Samples were removed from freezer, and 1ml of 4% paraformaldehyde solution in 0.75x PBS[1] (freshly diluted from a 10mL aliquot from EMS, RT 15710) was added. The sample was immediately vortexed for 10 seconds then the filter was removed and discarded. The fixation reaction was incubated on the bench for 2 hours, then we added 200μL 2.5M glycine and inverted the tubes to quench the fixation reaction. After all tubes were quenched, they were spun at 3000g 4 minutes RT, then supernatant aspirated and pellets washed again with 1x PBS. Samples were pelleted again and resuspended with 1mL of Buffer B[2].

Fresh spheroplasting solution was made, using 898μL Buffer B + 2μL 14.3M beta-mercaptoethanol + 100μL freshly denatured 200mM VRC (vanadyl ribonucleotide complex, NEB S1402S). This was kept at room temperature before adding 200 units per mL of lyticase (Sigma L5263) dissolved as 25U/μL in 1x PBS. Each sample was pelleted at 3000g, then 1mL of the spheroplasting solution with lyticase was used to resuspend the pellet, and tubes were incubated at 37°C for one hour. Microscopy monitoring of the reaction showed the typical greying of the cells under phase contrast microscopy to a dark grey, but did not digest to ghosts and fragments. After one hour incubation, all tubes were spun 1200g room temperature for 6 minutes. Most of the supernatant was carefully aspirated without removing the “fluffy” pellet, and Buffer B was added back to gently resuspend. Twice the sample was spun 5 minutes 1200g room temperature, supernatant carefully aspirated, and resuspended in cold (iced) Buffer B. Then, the sample was spun 5 minutes 1200g room temperature, and resuspended 80% ethanol then put in 4°C.

Hybridization

The samples were processed with a Quantigene Flow RNA kit purchased in March of 2015 (product code 15710), and designed for *GAP1* mRNA in *Saccharomyces cerevisiae*. The probe sequences are

[1] Phosphate buffered saline, made as in doi:10.1101/pdb.rec8247 .

[2] 1.2M sorbitol (from 2M filter sterilized stock), 100mM potassium phosphate, made to a pH of 7.5 by mixing 30mL 2M filtered sorbitol with 15.05mL hyclone water with 4.15mL 1M K_2HPO_4 and 0.8mL 1M KH_2PO_4.

proprietary. This procedure is largely as described by the manufacturer, with some critical modifications.

The incubator used was calibrated to 40°C using a Traceable 4004 Type-K thermometer. The probe was inserted into an eppendorf tube through a hole and sealed with parafilm, and inserted into the aluminum heatblock in the air incubator as used for incubating samples. This incubator was run overnight to check maintenance of the temperature, and was measured as 40.0°C in the morning. The ethanol-permeabilized samples from 4°C overnight (16 hours) storage were pelleted by centrifuging 1200g for 5 minutes room temperature. The supernatant was aspirated, and pellet washed once in 500μL Solution D (supplied with kit). The sample was pelleted at 3 minutes 1200g then the supernatant aspirated completely without perturbing the pellet. The pellet was resuspended in 25μL Solution D, then 25μL was added of the supplied *GAP1*-targeting probes appropriately diluted 1/20 in the Target Probe Dilutent. For a "no-probe" control, one sample of the library from the induced condition was resuspended in just Target Probe Dilutent. These were all incubated in the heatblock in the 40.0°C incubator. After one hour, all samples were briefly mixed with a vortexer to lightly stir the solution. After completing a total of 2 hours incubation, tubes were removed and 300μL of Wash Buffer was added. Tubes were inverted to mix, then spun 3 minutes at 800g room temperature. The supernatant was aspirated, and the pellet washed again in this way. The pellet was resuspended in 25μL Wash Buffer, then 25μL of Pre-Amplification mix (pre-warmed to 40°C) was added and mixed with pipette. Samples were incubated for another 1.5 hours, then were washed as above (two washes of 300μL wash buffer, then resuspension in 25μL wash buffer). These were mixed with 25μL pre-warmed Amplification mix. This was incubated for 1.5 hours, then washed as above and mixed with 25μL of Alexa-647 labeling-probe diluted 1/100 in Label Probe Dilutent. Samples were incubated for 1 hour, then washed once with Wash Buffer. Then, samples were washed for a 5 minute incubation in Wash Buffer with 500ng/mL DAPI added. Samples were spun and aspirated as above, then washed once with Wash Buffer and resuspended in 100μL Storage Buffer. All steps with the label probe and DAPI were kept in dark as much as possible. Samples of these were put on poly-L washed coverslips and imaged on an epi-fluoresence scope to confirm the *GAP1* mRNA FISH had been successful.

FACS

Samples were sorted using fluoresence-activated cell sorting into four quartiles of *GAP1* mRNA FISH signal.

90μL of the hybridized samples from above were put onto 410μL of freshly filtered 1x PBS. Samples were sonicated using a standard program used for preparation of yeast samples for coulter counter, here 10 power for 5 of 1 second pulses (Misonix CL5). The samples were kept on ice, under foil, until a NYU GenCore technician ran the samples through the department FACSAria II. The sorting strategy was to run each sample through to measure 10,000 events. Then, gates were defined to isolate singlets using forward and side scatter, then gate using the DAPI for DAPI-stained events, then the sample was sorted on the area of the signal from the 660/20 filter emission from a 633nm laser excitation (FISH probe is Alexa 647).
Importantly, the sorting gates were set with a GUI interface until they approximated splitting the libraries into quartiles for the six samples. Below are the gate boundaries:

Shifted	FACSGate	LowerBound	UpperBound
Pre-upshift	p2	-1199	566
Pre-upshift	p3	572	2589
Pre-upshift	p4	2641	7196
Pre-upshift	p5	7294	100000
Post-upshift	p6	-1199	39
Post-upshift	p7	39	330
Post-upshift	p8	330	971
Post-upshift	p9	975	100000

Note that we later in analysis add a fixed number to all observations and gates in linear space in order to get into positive values and avoid using any odd scales like biexponential.

The "sort report" is included at `data/dme209/dme209facsReport.pdf`, and contains reports of sorting gates, statistics, and plots of the singlet gating (top right plot), the DAPI gating (bottom left plot), and the sort on mRNA FISH (bottom right plot). The gate designators correspond to the below table. With regards to sample identifiers, "I2" is a control sample of the input library, "+" denotes hybridization with the target probes, "-" is a negative control. A, B, and C refer to the replicate. 1 is the pre-upshift sample, and 4 is the post-upshift sample, so C4 is replicate C post-upshift.

From the sort for collection, we obtained the following counts of events per gate:

BiologicalReplicate	FACSGate	Events	Timepoint
A	p2	1093173	Before upshift
A	p3	1028599	Before upshift
A	p4	918549	Before upshift
A	p5	734058	Before upshift
A	p6	1340056	After upshift
A	p7	1011347	After upshift
A	p8	959941	After upshift
A	p9	729971	After upshift
B	p2	1002007	Before upshift
B	p3	1028599	Before upshift
B	p4	1063116	Before upshift
B	p5	1006965	Before upshift
B	p6	1296444	After upshift
B	p7	1091615	After upshift
B	p8	1168612	After upshift
B	p9	1027629	After upshift
C	p2	468578	Before upshift
C	p3	407245	Before upshift
C	p4	426347	Before upshift
C	p5	476639	Before upshift
C	p6	548282	After upshift

BiologicalReplicate	FACSGate	Events	Timepoint
C	p7	421597	After upshift
C	p8	410400	After upshift
C	p9	358944	After upshift

After sorting using PBS sheath fluid at room-temperature, into poly-propylene FACS tubes, samples were capped and frozen at -20°C.

Cell collection and DNA extraction

From samples of sorted mutant cells, genomic DNA was extracted by reverse-crosslinking and proteinase K digestion.

Each sorted sample was thawed at room temperature, then vortexed. Samples processed were from replicates A, B, and C for timepoints "pre-upshift" and "post-upshift" for 4 different bins/gates of collection, each. We also prepared samples from replicates A, B, and C from post-hybridization samples before the FACS, to serve as "input" samples. Cells were pelleted by centrifugation according to the following strategy Each sample was centrifuged in low-bind silanized 1.5mL eppendorfs at 1200g for 5 minutes room-temperature. After each spin, half the supernatant was removed, then the same volume of sample added again. Thus, the approximately 8mL of sorted cells in cold PBS were carefully collected into 500μL volume. Then, this volume was reduced by repeatedly centrifuging, aspirating half the supernatant, then vortexing lightly and repeating the procedure. When samples were brought to minimal (<5μL) volume, this was resuspended and transferred to PCR tube strips using 50μL of reverse crosslinking buffer[3]. Another 50μL of reverse crosslinking buffer was used to wash the collection eppendorf (with vortexing) into the PCR tube.

Collected cells were digested to reverse crosslinks and lyse. To the PCR tubes with cells in reverse crosslinking buffer, 5μL proteinase K (Ambion AM2546) was added, and PCR tubes incubated in a 65°C PCR machine with heated lid. After 13 hours, the machine was set to 80°C for 5 minutes, then cooled to 37°C. To this, 5μL RNAseA was added and incubated for 30 minutes. Then 5μL of proteinase K was added again, and incubated at 37°C for 1 hour. Temperature was upshifted to 80°C for 10 minutes, then to RT. These were transferred to new low bind silanized eppendorfs, and PCR tubes washed again with 85μL reverse crosslinking buffer added to the eppendorfs. Samples were kept at 4°C.

DNA was extracted from each sample by adding 200μL 25:24:1 buffered-phenol:chloroform:isoamyl-alcohol, and vortexing well. This was 1 minute 15,000g room temperature, then 195μL of the top layer was transferred a new tube. The bottom phase of the extraction was back-extracted by adding 100μL of the reverse-crosslinking buffer, vortexing, and spinning again to take aqueous to the extracted top layer from before. To this ~300μL of aqueous extraction, 300μL chloroform was added and the whole mixture put onto a pre-spun phase-lock gel tube (5Prime #2302830). After a 5 minute 15,000g spin, this was transferred to a new tube with 9μL glycogen and 750μL 100% ethanol. This

[3]Reverse crosslinking buffer: 0.5% SDS, 250mM NaCl, 10mM Tris, 1mM EDTA. Inspired by Klemm et. al. 2014's procedure.

was incubated on ice for one hour, then spun 30 minutes 4°C maximum speed. The non-visible pellet was washed with 80% ethanol twice, then dried for 10 minutes and resuspended with 35μL hyclone water with rounds of vortexing and spinning at room temperature. This extracted gDNA was stored at -20°C.

Construction of amplicon sequencing libraries for barcode counting

gDNA was amplified in a heavily modified BarSeq protocol, referred to internally as SoBaSeq (Sorted Barcode Sequencing).

Motivation

First, we motivate this protocol, as it departs from previous procedures in a few ways. This can be skipped, as the protocol details are in the next section labeled "Protocol".

The main impetus for this was the generation of primer dimers that form when the forward universal primer primes off the reverse universal primer. For example, figure 1 shows a failed experiment that shows dimer formation in the sample lanes (on right). In the right five samples, there's a balance of the expected product (top bands) and the dimer (lower bands).

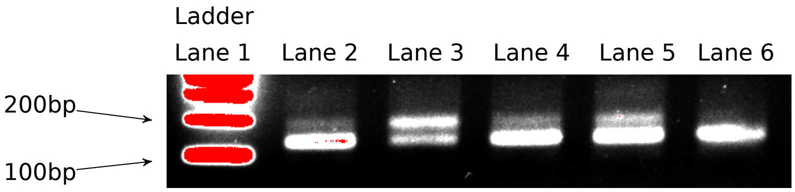

Figure 1: This is a 3% TAE agarose gel, stained with Sybr Safe dye. The left-most lane is a NEB 100bp ladder, with the bottom two bands as 100bp and 200bp. The red is due to overexposure. The right five bands are from samples prepared with an earlier version of this protocol. The band approximately 190bp is throught to be the library product, and the band approximately 160bp is thought to be the inhibitory and unwanted barcode-less dimer. Lane 3 clearly shows both bands, while lane 6 is all dimer.

Below a critical threshold, this dimer greatly out-competes the desired product and can result in a loss of amplicon before the amplicon is amplified enough to gel extract (above figure, lane 6). The dimer is also sequenced via Illumina chemistry (not desired). By Sanger sequencing we found that it appears to result from a three base trucation of the forward primer priming perfectly for about 6 bases off the reverse primer. This was not solved by switching to a polymerase without 3' exonuclease activity, or by using HPLC purified primers. Using different reverse primers lead to off-target products.

We saw these dimers before incorporating a UMI step into the protocol. We used a UMI because we wanted the protocol to be as quantitative as possible, despite the multiple amplification steps that would introduce randomly sampled noise at each cycle. The design of this was 6 bp degenerate sequence spaced with fixed bases, in the design of `NCNCNCNTNCN` because we estimated this

would best block annealing to any 3' ends of the primers used. In future work, we would strongly recommend using more degenerate bases for such a low-complexity library[4]. In order to digest the excess un-incorporated UMI primers it requires the addition of a exonuclease. ExoI is characterized to be maximally effective at 37°C, and although it can have activity at 42°C for a some time[5] it will be inactivated. This low temperature requirement likely exacerbates dimer and off-target product formation.

To address this, we optimized the reactions on a dilution series of gDNA from a different experiment with the same knockout library. By balancing $MgSO_4$ and glycerol concentrations we got better amplification, and tried to use a "booster" [6] PCR approach. This gave some improvements in how low we could detect before saturating the reaction with dimers, but we could not go lower in primer concentration and attributed this to the predicted secondary structure in the 3' end of the primer amplifying from the outside of the UPTAG barcodes. Adding DMSO helped with this, but we still had to leave the reaction with plenty of primer as intra-molecular interference from this process would out-compete inter-molecular productive annealing. We still could not get reliable amplification from $< 10^5$ templates (esimated by qubit assuming 12.5 picograms gDNA per genome).

The major solution to this problem was the addition of 3' phosphorlyated blocker oligos. These are not extended by DNA polymerases but are displaced by a strand-displacing polymerase like Vent exo-. By using this polymerase and blocker combo, we prevent new 3' ends from annealing but allow properly annealed primers to extend through this region. This, in combination with the exonuclease digestion of most of the reverse primer, prevented dimer formation. This revealed that these universal priming sequences will amplify from two loci near *CIA1* and *RDN37*. This was identified by Sanger sequencing gel-extracted bands, so we designed more 20-mers that again block off-target annealing and found they worked wonderfully. In test experiments, we believe we got amplicons of the correct size from as low as ~ 300 targets but have not sequence verified this.

To simplify the addition of the last 5' Illumina P5 adpater, we kept this as a separate reaction. To minimize chimera formation between different samples in this reaction, this is a 2-step polymerase extension reaction which partially forms the sequencing product (1/3 of results, theoretically). This is sufficient for qPCR quantification of the library and Illumina sequencing. Given our gel-extraction clean up and small product size, we do not expect formation of chimeras on the flow cell [7].

We welcome discussion, criticism of, or opportunities to support further refinement of low-input barcode sequencing. Figure 2 shows a cartoon of the amplicon library-making procedure:

---

[4] Fu et. al. 2011 *PNAS*

[5] Fei et. al. 2015 *PLoS One*, NEB R&D

[6] Ruano, Fenton, and Kidd 1989 *NAR*

[7] http://dnatech.genomecenter.ucdavis.edu/2017/04/11/update-on-barcode-mis-assignment-issue/

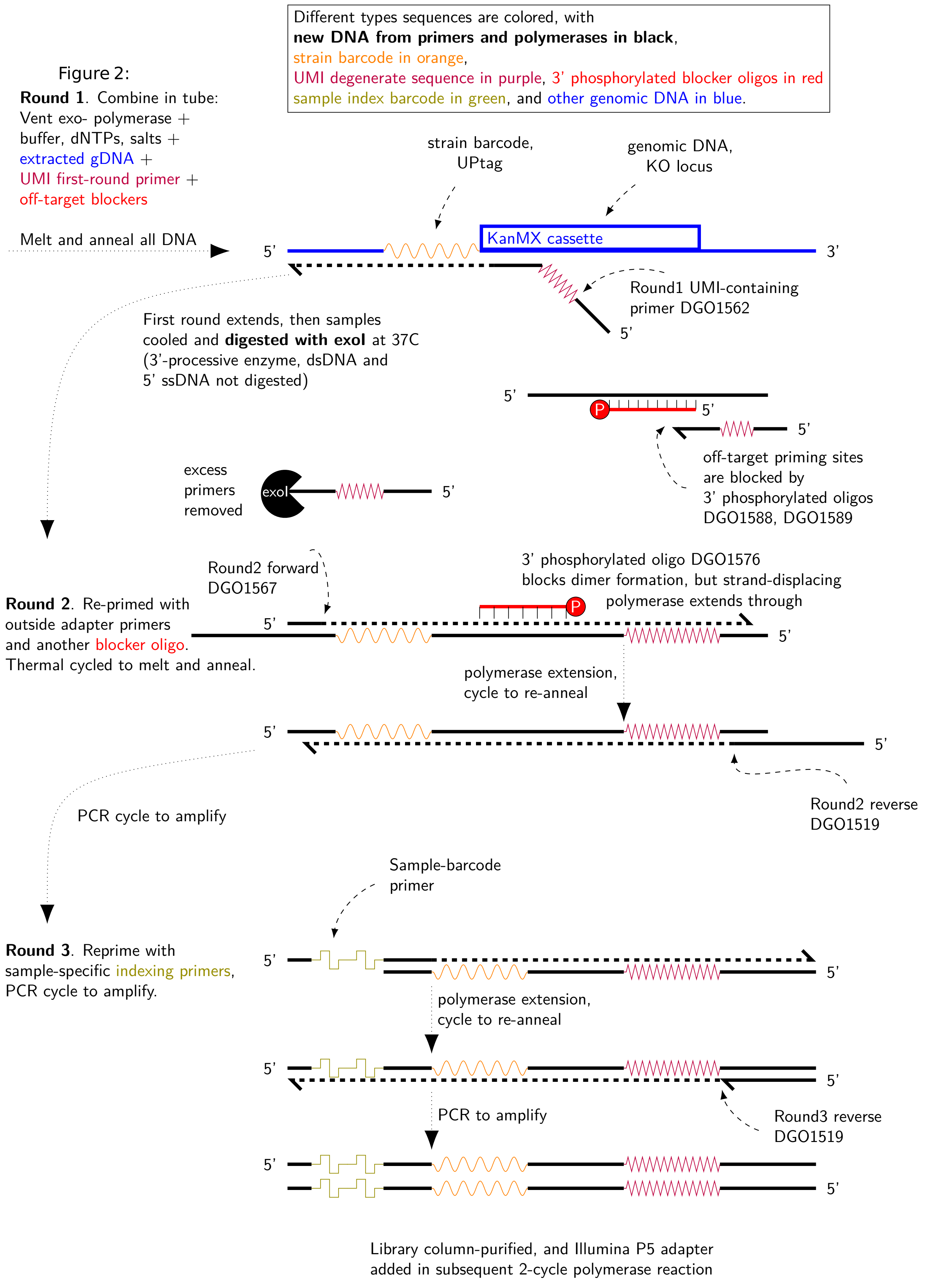
Different types sequences are colored, with
new DNA from primers and polymerases in black,
strain barcode in orange,
UMI degenerate sequence in purple, 3' phosphorylated blocker oligos in red
sample index barcode in green, and other genomic DNA in blue.
Figure 2:
Round 1. Combine in tube:
Vent exo- polymerase +
buffer, dNTPs, salts +
extracted gDNA +
UMI first-round primer +
off-target blockers
Melt and anneal all DNA
strain barcode,
UPtag
genomic DNA,
KO locus
KanMX cassette
5'
3'
Round1 UMI-containing
primer DGO1562
5'
First round extends, then samples
cooled and digested with exoI at 37C
(3'-processive enzyme, dsDNA and
5' ssDNA not digested)
5'
P
5'
5'
off-target priming sites
are blocked by
3' phosphorylated oligos
DGO1588, DGO1589
excess
primers
removed
exoI
5'
Round2 forward
DGO1567
3' phosphorylated oligo DGO1576
blocks dimer formation, but strand-displacing
polymerase extends through
P
Round 2. Re-primed with
outside adapter primers
and another blocker oligo.
Thermal cycled to melt and anneal.
5'
5'
polymerase extension,
cycle to re-anneal
5'
5'
PCR cycle to amplify
Round2 reverse
DGO1519
Sample-barcode
primer
Round 3. Reprime with
sample-specific indexing primers,
PCR cycle to amplify.
5'
5'
polymerase extension,
cycle to re-anneal
5'
5'
PCR to amplify
Round3 reverse
DGO1519
5'
5'
Library column-purified, and Illumina P5 adapter
added in subsequent 2-cycle polymerase reaction

Protocol

The first round master mix was composed of:

- 4.2μL hyclone water
- 2μL dNTPs (2.5mM each) (NEB N0447S)
- 2μL 10x NEB ThermoPol (NEB B9004S)
- 0.6μL $MgSO_4$ 100mM (NEB N0257L)
- 0.2μL BSA (20mg/mL (NEB B9000S)
- 0.2μL DGO1562 10μM
- 0.2μL DGO1588 1μM
- 0.2μL DGO1589 1μM
- 0.4μL Vent exo- (NEB M0257L)

10μL of this master-mix was put into individual 0.2mL PCR tubes (PP, domed), then 10μL of extracted genomic DNA template in hyclone water was added and mixed with pipette.

30 reactions were run in each batch of preparation. All 30 reactions were put into a BioRad T100 thermocycler, set for a 30μL reaction volume with a 95°C heated lid. These were cycled through

- 95°C 4 minutes
- 50°C 30 seconds
- 72°C 30 seconds (ramped at 1°C/s)
- 37°C hold

The lid was opened and tubes opened five at a time. To each tube, 2μL of a master mix of 0.25μL exoI (Thermo EN0581) diluted with 1.75μL water was added and mixed with five strokes of pipette. Lids were replaced, with the whole process for 30 tubes taking about 3 minutes. Tubes were incubated

- 37°C 10 minutes, with heated lid
- All tubes were removed to be lightly vortexed and quick spun to pull liquid to bottom again, then replaced in thermocycler
- 37°C 10 minutes, with heated lid
- 50°C 5 minutes
- 80°C 5 minutes
- 60°C hold

To each tube, opened five lids at a time, 5μL of the round 2 master mix was added. The round 2 master-mix:

- 0.51μL hyclone water
- 0.7μL 10x NEB Thermopol buffer
- 2.7μL 50% glycerol (diluted with hyclone water from 100% stock)
- 0.21μL $MgSO_4$ 100mM
- 0.07 BSA 20mg/mL
- 0.27μL of DGO 1576 at 10μM
- 0.27μL of DGO 1567 at 1μM
- 0.27μL of DGO 1519 at 10μM

These were mixed with five strokes of pipette, the whole process took about 3 minutes. This was incubated

- 95°C 1 minute
- 40 (forty) cycles of
  - 95°C 15 seconds
  - 54°C 15 seconds
  - 72°C 20 seconds
- 60°C hold

5μL of round 3 master mix was added and mixed with two strokes of pipette. This is composed of

- 1.6μL of hyclone water
- 0.2μL of 10x NEB Thermopol buffer
- 0.6μM of DGO 1519 10μM
- 0.6μM of forward Barseq indexing primer 10μM (indexing primer was added to which samples was as in the CSV sample sheet[8] in the data folder in the `data.zip` archive)

This was incubated

- 95°C 15 seconds
- 50°C 15 seconds
- 72°C 15 seconds
- 12 (twelve) cycles of
  - 95°C 15 seconds
  - 68°C 30 seconds.
- 72°C 30 seconds (this is a final step, separate from the above cycling procedure)

After this, all samples were immediately put on ice and then frozen at -20°C.

According to the chart, we pooled reactions into four QC pools, based on expected similarity of the library. Each of the four QC pools had 5μL of each reaction pooled together and cleaned up on one MinElute column for each QC pool, and eluted into a lowbind eppendorf with 20μL hyclone water. Each pool was quantified with qubit, with a range from 27.0 ng/μL (pool3) to 57.9 ng/μL (pool4). Thus, approximately 1.86×10^{12} molecules were produced for a pool of approximately 20-30 samples.

We expect the libraries at this stage to be these sequences:

Figure 3:

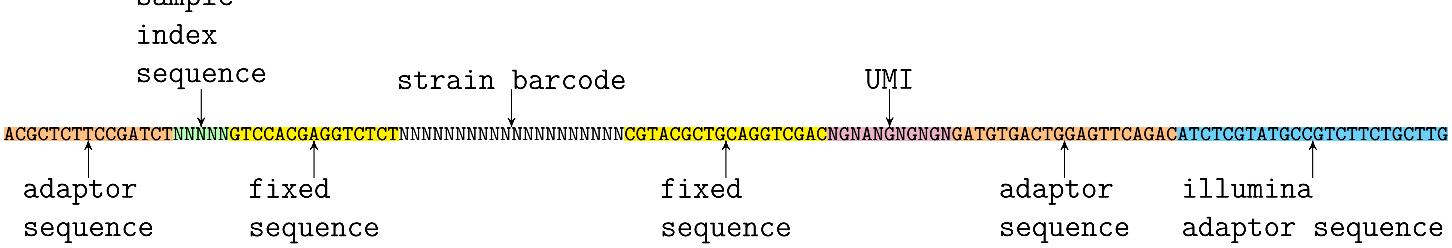

[8] `data/dme209/dme209.SampleSheet.csv`

These were checked with sanger sequencing, for pools 1 and 3, using primers DGO 276 or DGO 1519, with Genewiz sequencing. Representative Sanger sequencing image of library pool 1, sequenced forward (with DGO 216) is shown in figure 4.

Trace colors: red is T, green is A, blue is C, black is G.

Figure 4:

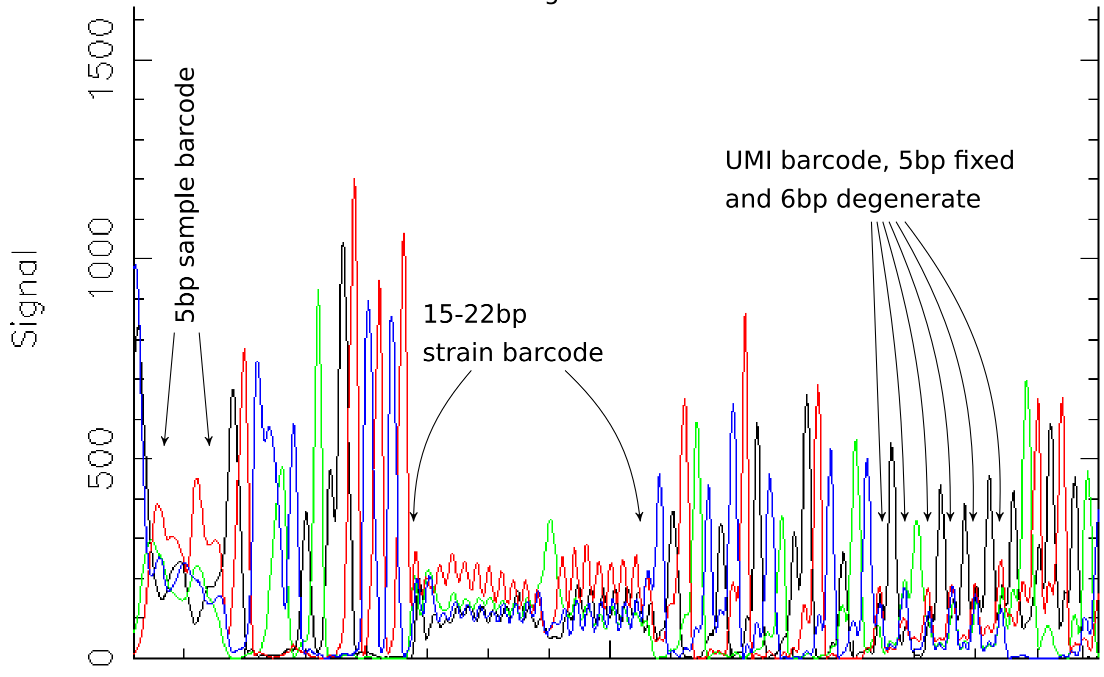

To add on the Illumina P5 adapter (P7 is already incorporated, cyan in previous diagram), this requires one more polymerase reaction. Using 3μL of the first three pools and 2μL of Pool 4, We set up reactions using a master mix, with each reaction composed of:

- 1.2μL NEB dNTPs 2.5mM each
- 1.5μL 10x Thermopol NEB
- 0.45μL $MgSO_4$
- 0.75μL DGO 1519 10μM
- 0.75μL DGO 276 10μM
- 0.4μL Vent (exo-) polymerase NEB
- template (see above) and water to bring the reaction to 15μL total

These reactions were run on a thermocycler (AB GeneAmp PCR System 9700) in the following procedure:

- 95°C 1 minute
- 52°C 30 seconds
- 72°C 1 minute
- 95°C 30 seconds
- 68°C 30 seconds

- 72°C 1 minute
- 4°C, then onto ice

The entire reaction was run on a 3% agarose TAE gel, where it was clear that a second larger product had formed above the band from before, although the bands were very near overlapping. Both bands were cut out and purified using what is essentially a Qiagen kit (QC dissolving the gel, then through a column), but using the MinElute columns and eluting with 20μL hyclone water. By qubit, these were estimated to be about 37.7 to 55.7 nM, assuming a product size of 171bp (expected with a 20bp barcode). This is a mixture of products with the P5 adapter or not, and so was quantified using qPCR with KAPA Illumina Quantification Kit standards and master mix (REF 07960298001), then diluted to 4nM and submitted to the NYU GenCore sequencing core for sequencing on a 1x75bp run on a Illumina NextSeq with 5% PhiX spiked in.

This run only yielded approximately 127 million reads (out of 400 million listed yield), likely due to the NextSeq base calling software being confused by fixed sequences. 5% PhiX is too low for this, but considering that most failures occurred in the fixed sequence upstream of the barcode, we could not imagine a way in which this failure would specifically affect certain strains or not. Thus, we continued with the data that survived the base-calling failure, and recommend a higher PhiX spike-in fraction (~25%) for future experiments.

Analysis

Designing an analysis pipeline to incorporate UMI information

We previously used the one-program solution of BarNone [9] to rapidly and easily quantify barcode counts from yeast barcode sequencing experiments. However, our new amplicon design makes use of UMIs to help account for the amplicon noise inherent in the BarSeq method, and BarNone does not account for these. We also wanted to devise a pipeline that would be modular, consisting of multiple well-designed tools that could be modified independently and would maintain read information along the pipeline to assist in debugging and benchmarking.

We designed such a system. Here is the work-flow:

1. FASTQ files of the reads are fed into a custom python script called `SLAPCHOP.py` [10]. This parallelized script takes each read, aligns the expected fixed sequences that bracket the informative barcodes (using BioPython[11]), decides if the read matches the expected structure based on a specified criteria (see the README [12]), then extracts out the sample index, strain barcode, and UMI degenerate sequence into appropriate positions in a FASTQ format. This keeps the filtering and strain barcode identification separate from the fixed sequences.
2. A simple perl script (`pickyDemuxer.pl`) demultiplexes the processed FASTQ file on perfect matches of the 5bp index sequence and generates a demultiplexing report.

[9] http://varianceexplained.org/BarNone/
[10] Named after the cooking utesil of infomercial fame. https://github.com/darachm/slapchop
[11] Cock et al. 2009
[12] Named after the cooking utesil of infomercial fame. https://github.com/darachm/slapchop

3. The strain barcode regions, padded with flanking sequence to a uniform length of 26bp, from the demultiplexed FASTQ files are aligned using `bwa mem`[13] to the expected barcodes as re-annotated by Smith et. al. 2009 (Genome Research).
4. From the resulting `bam` alignment files, we extract the strain identification and UMI. Using the UMI-collision / label-saturation concept and equation of Fu et. al. 2011 (PNAS), we adjust the saturated pool to estimate the input of strain genomes into the library preparation.

This allowed us to recognize and extract barcodes from indeterminate positions in the amplicon and filter the reads for real, intact amplicons in one step. This extraction improved our accuracy, for example eliminating spurious alignments of the forward priming sequence against the barcode of *ymr258c*Δ, a barcode re-annotated with striking similarity to the fixed priming sequence (Smith et al. 2009).

There are several different ways to use UMI information to estimate unique input molecules from a sequencing assay. The naive approach is to assume that every combination of strain barcode with a certain UMI sequence in a sample is a unique event, and any repetitions of this are duplicates. Error-correcting algorithms [14] exist that use graph information to improve accuracy of this method, but these require that the space of all usable UMIs is sparse. Here, we only have $4^6 = 4096$ possible UMIs for ~ 4500 possible strain barcodes, with approximately a million reads per sample. Thus, due to a short length of UMI we cannot make this assumption, and instead are confronted with a space of UMIs with a fairly high chance of two UMI-strain combinations being generated by random chance alone. We refer to this as a UMI-collision (similar concept to a hash collision) or the phenomenon of label saturation. The best solution for this appears to be the label saturation correction of Fu et. al. 2011 (PNAS). This depends on treating the chance that any UMI-collisions are a random and rare event, thus modeled as a Poisson distribution. If we have 4096 possible UMIs, and for one strain in one sample we observe x different UMIs associated, then we estimate that there were z different original molecules in the sample, where

$$z = 4096(1 - e^{-\frac{x}{4096}})$$

Figure 5 is another figure that explores this idea, and comes from the actual sequencing data from the experiment.

[13]Obtained from http://bio-bwa.sourceforge.net/ , reported in Li 2013.
[14]https://github.com/CGATOxford/UMI-tools

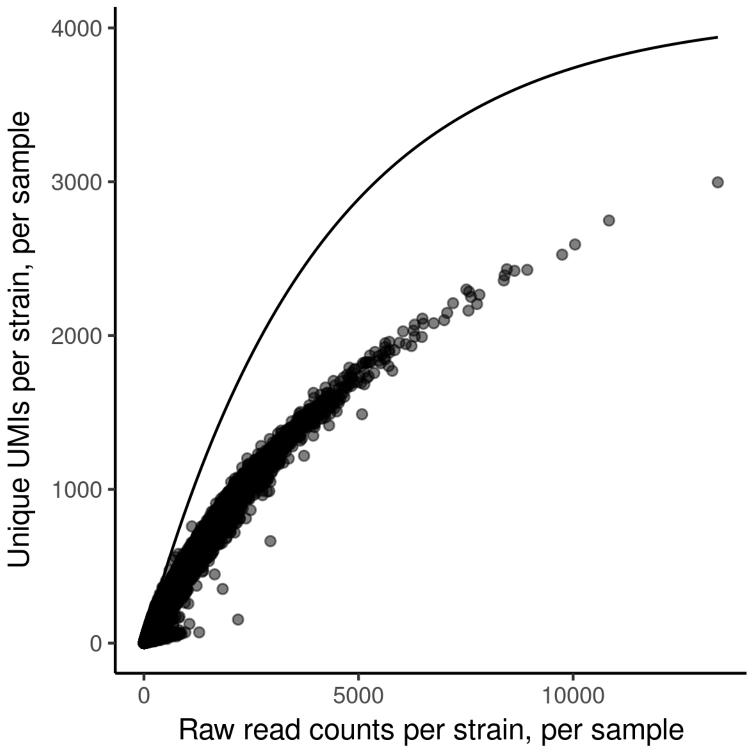

Figure 5: For each sample, each combination of UMIs and strain barcodes was collected. For each point, the y-axis denotes the unique UMIs observed for that combination of strain and sample. On the x-axis is the raw counts of observing that strain barcode in that sample. The line denotes the curve expected just from label saturation with increased re-sampling of a limited pool as it approaches 4096.

We see that the actual observations (the points) follow a similar pattern as the expectation, saturation towards the higher end of counts. However, they are greatly depressed below this line. Therefore, we believe that this shows that both label saturation and PCR duplication are at play to distort the mapping between either raw counts or unique labels and the actual underlying estimate of input genome targets per strain in each sample.

Benchmarking this pipeline

We benchmarked this pipeline against an *in silico* dataset to determine performance across a range of mutation rates and read depths similar to what we would expect for this experiment. We also compared the two UMI correction approaches described in the previous section, the "UMI-collision correction" and the "number unique" method. We used a python script `makingFakeReads.py` to generate several datasets with the following parameters:

- 16 million reads per FASTQ, split amongst 32 samples
- each strain barcode is sampled from an emipirically observed distribution averaged from the first timepoints of an unpublished dataset, quantified by BarNone

- each amplicon has a poisson number of random single nucleotide mutations to a different base, based on a given parameter of 0, 1, 2, or 3 lambda of mutations per amplicon.
- each generated amplicon is added to the file x number of times, where x is an exponential distribution with mean 5
- 3 "biological" replicate datasets are generated per set of parameters

After quantification, we calculate pearson correlation, spearman correlation, and the number of mistaken strain-identifications. Tolerated mismatches is a parameter set in BarNone or by the score requirements for alignment in `bwa`.

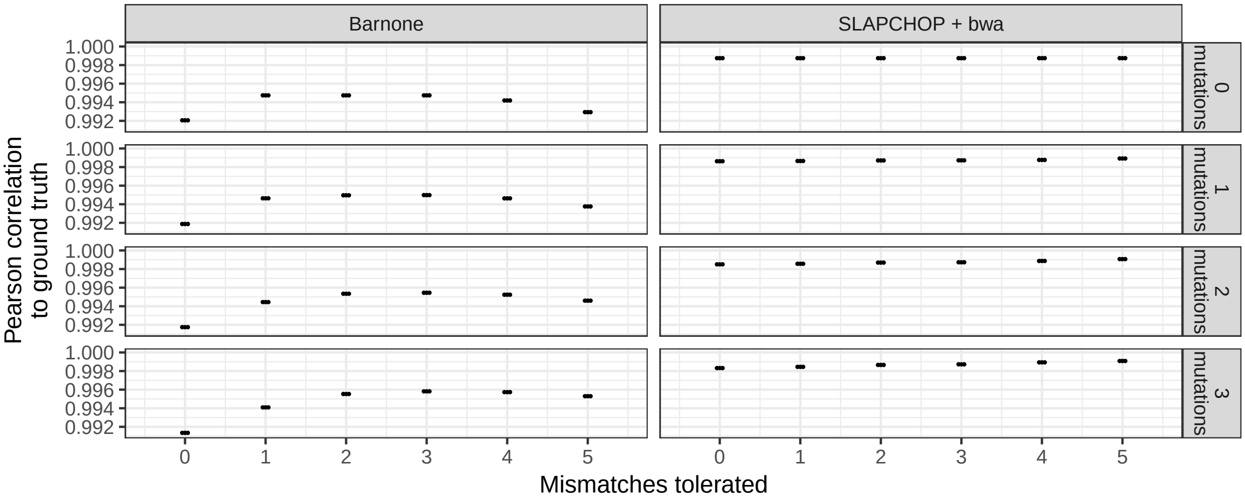

Figure 6: In-silico test data, comparing whole-sample quantification of un-demultiplexed samples, with two different methods of strain barcode identification. Pearson correlation to ground truth.

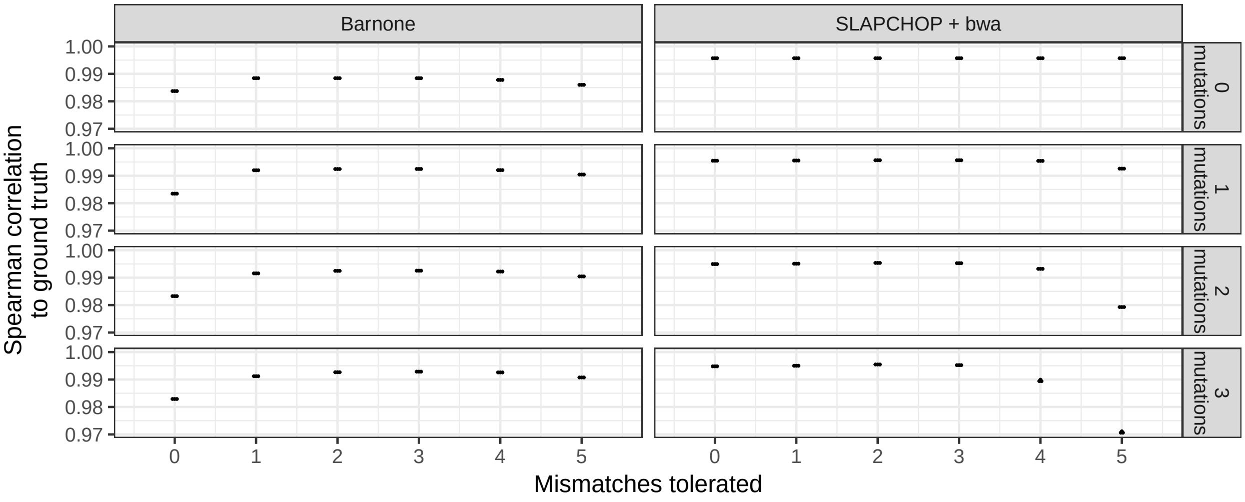

Figure 7: In-silico test data, comparing whole-sample quantification of un-demultiplexed samples, with two different methods of strain barcode identification. Spearman correlation to ground truth.

It would appear that by pearson correlation, the filtration step of the `bwa` alignment allows us to make more robust assignment of strain barcodes. The spearman correlation tells us that as mutation rate increases, high mismatch tolerance on the `bwa` tool is very dangerous for misaligning and can cause large rank changes.

How about UMI de-duplication methods?

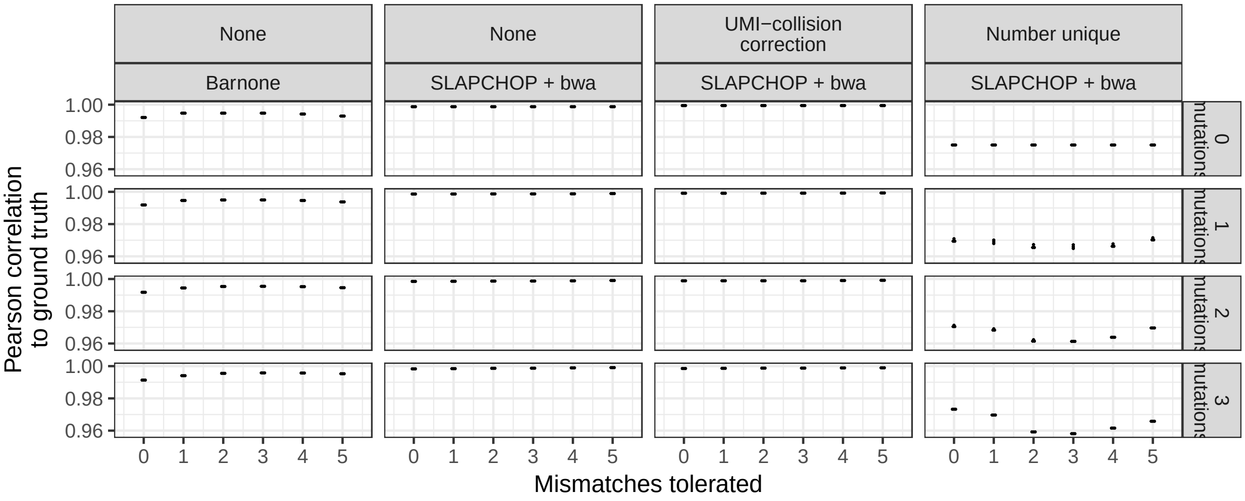

Figure 8: In-silico test data, comparing un-demulitplexed samples between different de-duplication methods and quantification tools. Pearson correlation. Top row denotes UMI de-duplication method.

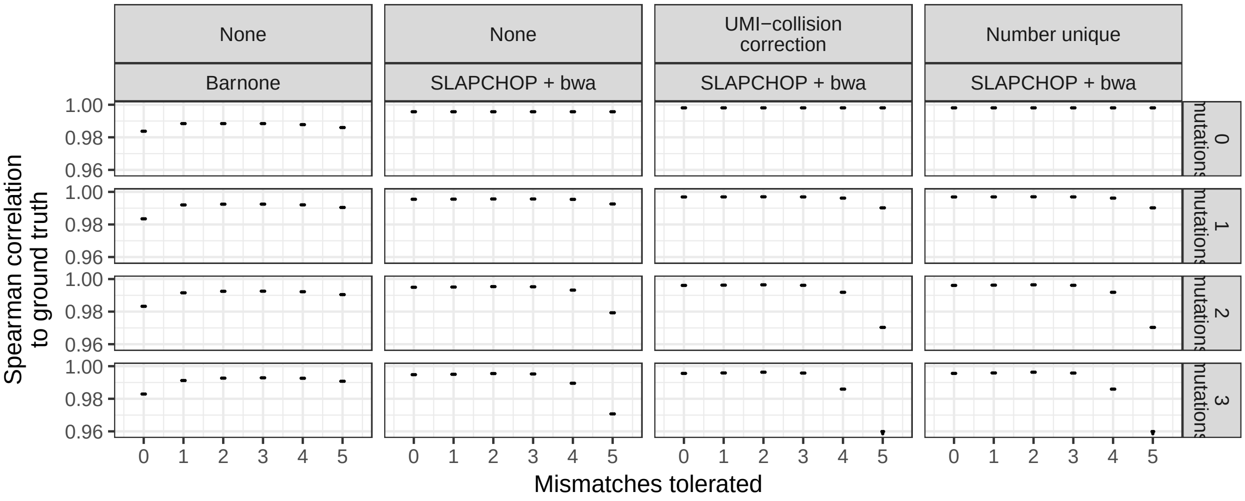

Figure 9: In-silico test data, comparing un-demulitplexed samples between different de-duplication methods and quantification tools. Spearman correlation. Top row denotes UMI de-duplication method.

We see that on the whole, un-demultiplexed datasets (16 million reads across ~4000 strains and

4096 possible UMIs) that the performance is best with the UMI-collision correction. We see that just using unique UMI counts in this regime leads to a good reconstruction of the rank order (Spearman), but inaccurate of the magnitude (Pearson) against ground truth.

Does this change with lower read density? How does our demultiplexing do? Each point is one of 32 demultiplexed samples of three whole-library replicates.

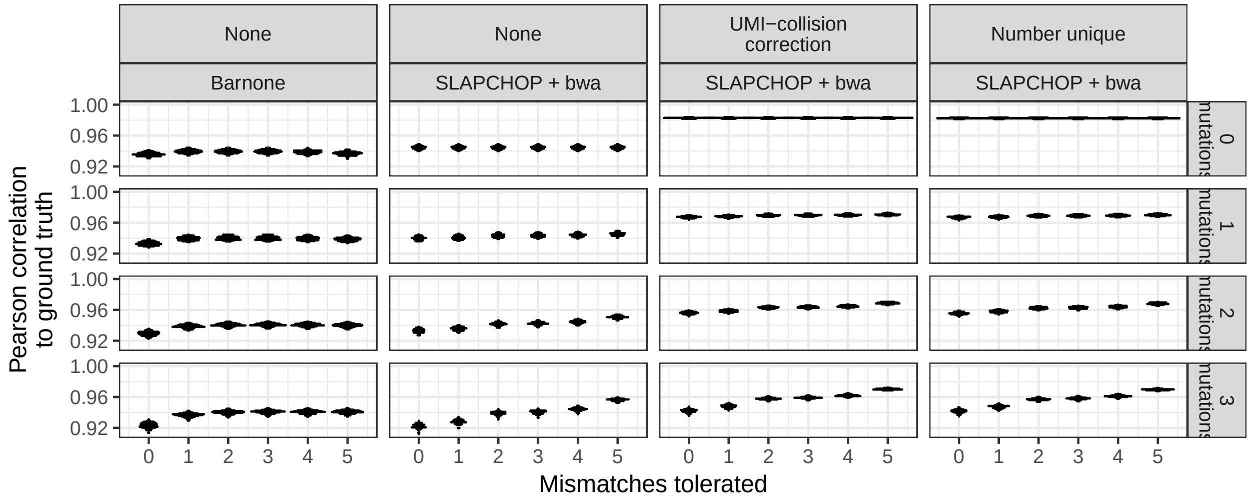

Figure 10: In-silico test data, comparing demulitplexed samples between different de-duplication methods and quantification tools. Pearson correlation.Top row denotes UMI de-duplication method.

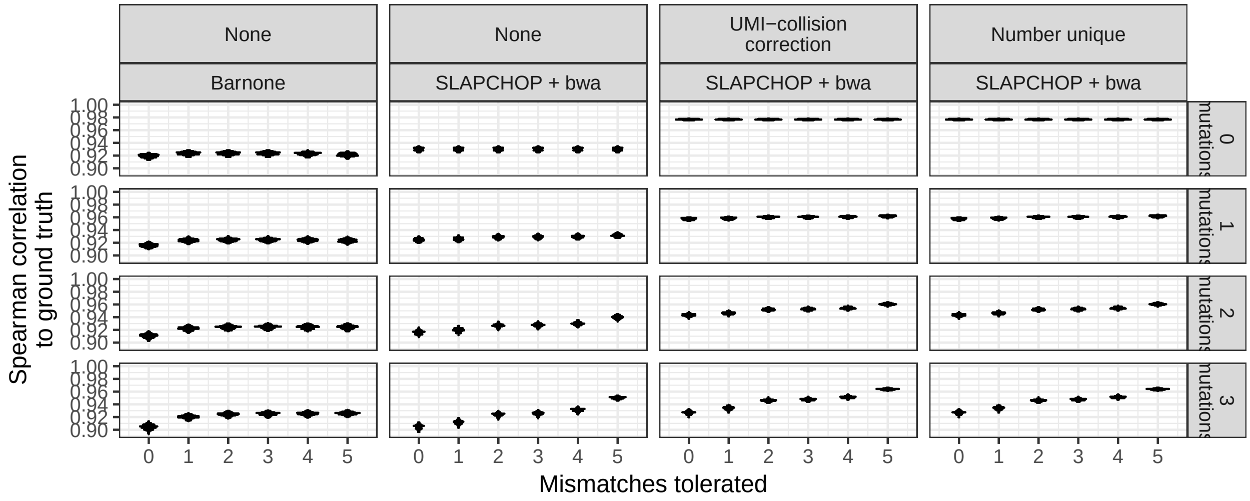

Figure 11: In-silico test data, comparing demulitplexed samples between different de-duplication methods and quantification tools. Spearman correlation. Top row denotes UMI de-duplication method.

BarNone appears to be more robust to mutations, in that it maintains a flatter profile in the higher

regimes of mutations. However, `bwa` starts out higher. Accounting for duplication, as generated by the exponential distribution described above, greatly improves performance. At an average coverage here of a half-million reads, the difference between the UMI-collision and unique counts is slighter.

In conclusion, our pipline for analysis is able to use an early filtration step[15] to improve strain barcode identification and to extract UMIs that are useful for de-duplicating PCR duplicates back to better estimates of the ground truth.

Modeling *GAP1* FISH signal per strain in the pool

In order to use the counts of each mutant in each sample to estimate *GAP1* mRNA abundance per strain, we used a maximum-likelihood modeling approach.

We are interested in the number of cells of a certain strain that went into each bin. We estimate this as a metric we define as "pseudocounts", or u_{ik} where i is the strain index, and k is the FACS bin. We call the sequencing counts c_{ijk}, where j is the particular PCR replicate out of J PCR replicates that were successfully sequenced. We assume the sequencing counts are linearly amplified from the "events" of actual cells being sorted into each collection tube, and we assume that all of these "events" have equal chance to be amplified and detected by this sequencing assay. Then we scale this estimate by the total number of "events" we observed during the FACS procedure going into each bin e_k. We assume that all "events" had equal chances in all bins to get sequenced. Then we have

$$u_{ik} = \frac{\sum_j \frac{c_{ijk}}{\sum_i c_{ijk}}}{J} \quad \frac{e_k}{\sum_k e_k}$$

This is intuitively more simple than the notation used here to describe it precisely. Since we split the library into quartiles for the sequencing, $\frac{e_k}{\sum_k e_k}$ is about one quarter for each bin. $\frac{c_{ijk}}{\sum_i c_{ijk}}$ is just the proportion of counts in that sample that are that mutant i. $\frac{\sum_j \frac{c_{ijk}}{\sum_i c_{ijk}}}{J}$ is simply the average of the proportion of counts, across the PCR replicates.

$\sum_k u_{ik}$ is going to be pretty much the proportion of the original library that is the mutant. So then if we divide u_{ik} by this, then we'll have our estimate of the proportion of that mutant that went into each bin, out of all the mutant that was in the experiment.

Once we have this normalized pseudocounts metric within each biological replicate, then we fit a log-normal model. We explored a logistic model and several mixture models (similar to DNA content flow cytometry with two log-normals and a middle quasi-uniform distribution), and found that the log-normal robustly fit well. The log-normal and logistic largely agreed on ranking of estimated means, but the likelihood was slightly higher for the log-normal fits on the whole library, so we used that model.

[15] Named after the cooking utesil of infomercial fame. https://github.com/darachm/slapchop

From this model (fit using `mle()` in R), we used the estimated mean as the estimate for the GAP1 abundance for that strain in that sample.
