# Supplementary figures and images for: Systematic identification of factors mediating accelerated mRNA degradation in response to changes in environmental nitrogen
Source: PLoS Genet. 2018 May 21;14(5):e1007406. doi: 10.1371/journal.pgen.1007406 (PMC5983874; doi:10.1371/journal.pgen.1007406)

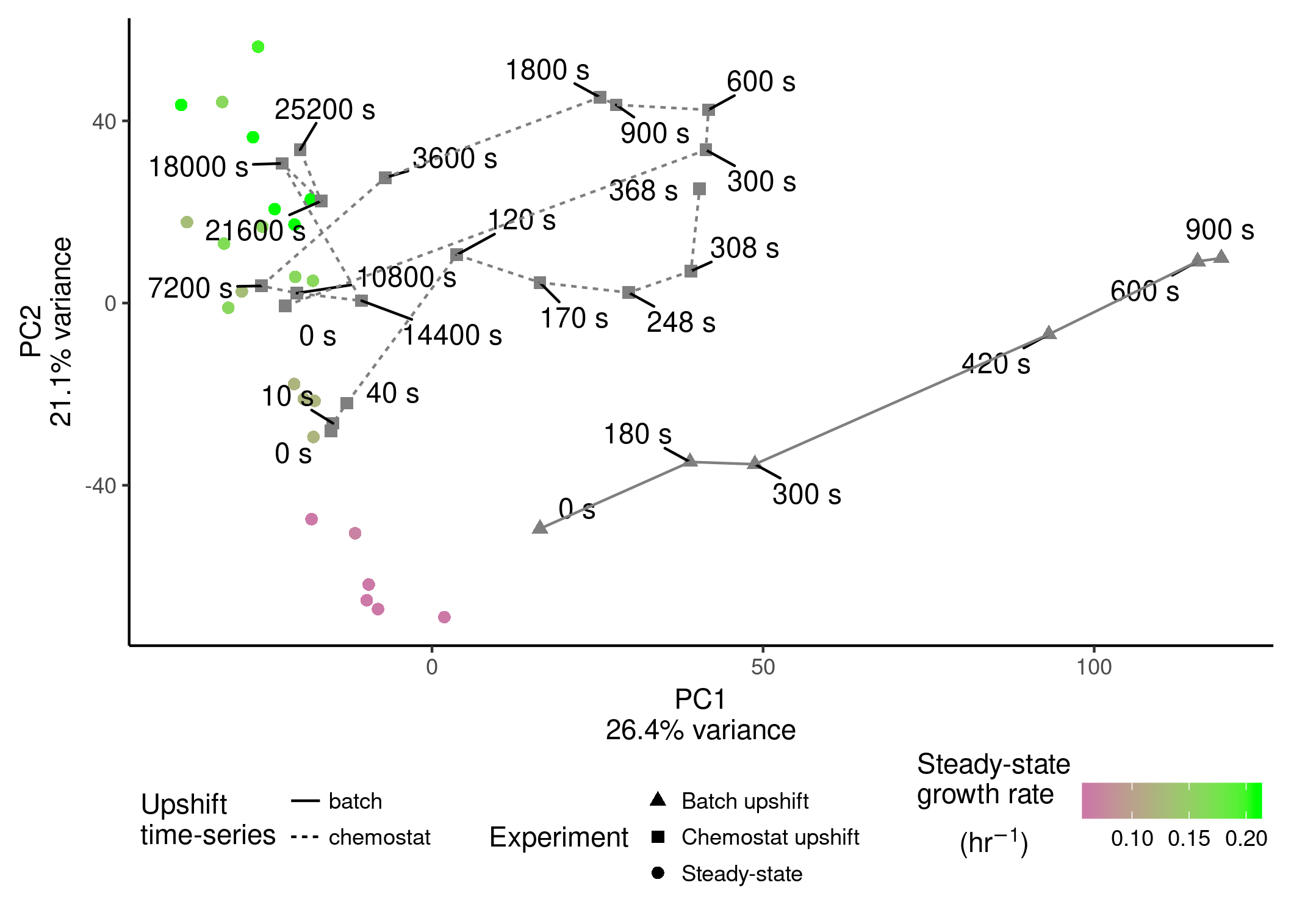

Supplement: S1 Fig — Principal components analysis (SVD) of microarray data from [25]. Colored points are from steady-state chemostats grown in limitation for various nitrogen sources, at different growth rates. Time-series experiments are show in grey points, connected by lines, and line-type is the type of upshift (in batch or in chemostat). (TIFF) [file pgen.1007406.s015.tiff]

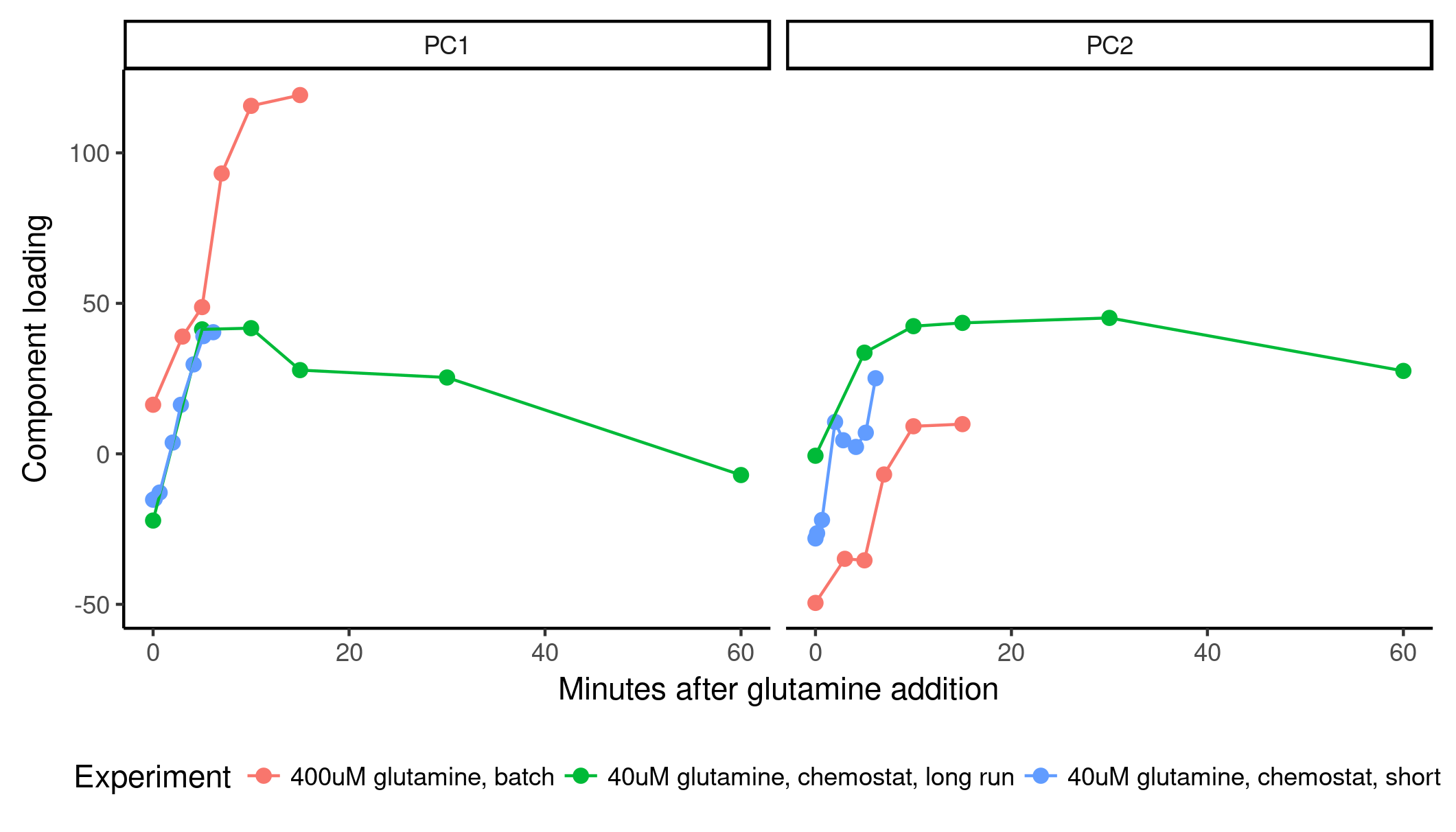

Supplement: S2 Fig — The changes in loadings in the first two principal components of microarray data analyzed from [25], as in Fig 1C, for chemostat upshift experiments only. In the chemostat, addition of 400μM glutamine has a more pronounced response than 40μM glutamine addition, but all have a sharp response in short timescale. (TIFF) [file pgen.1007406.s016.tiff]

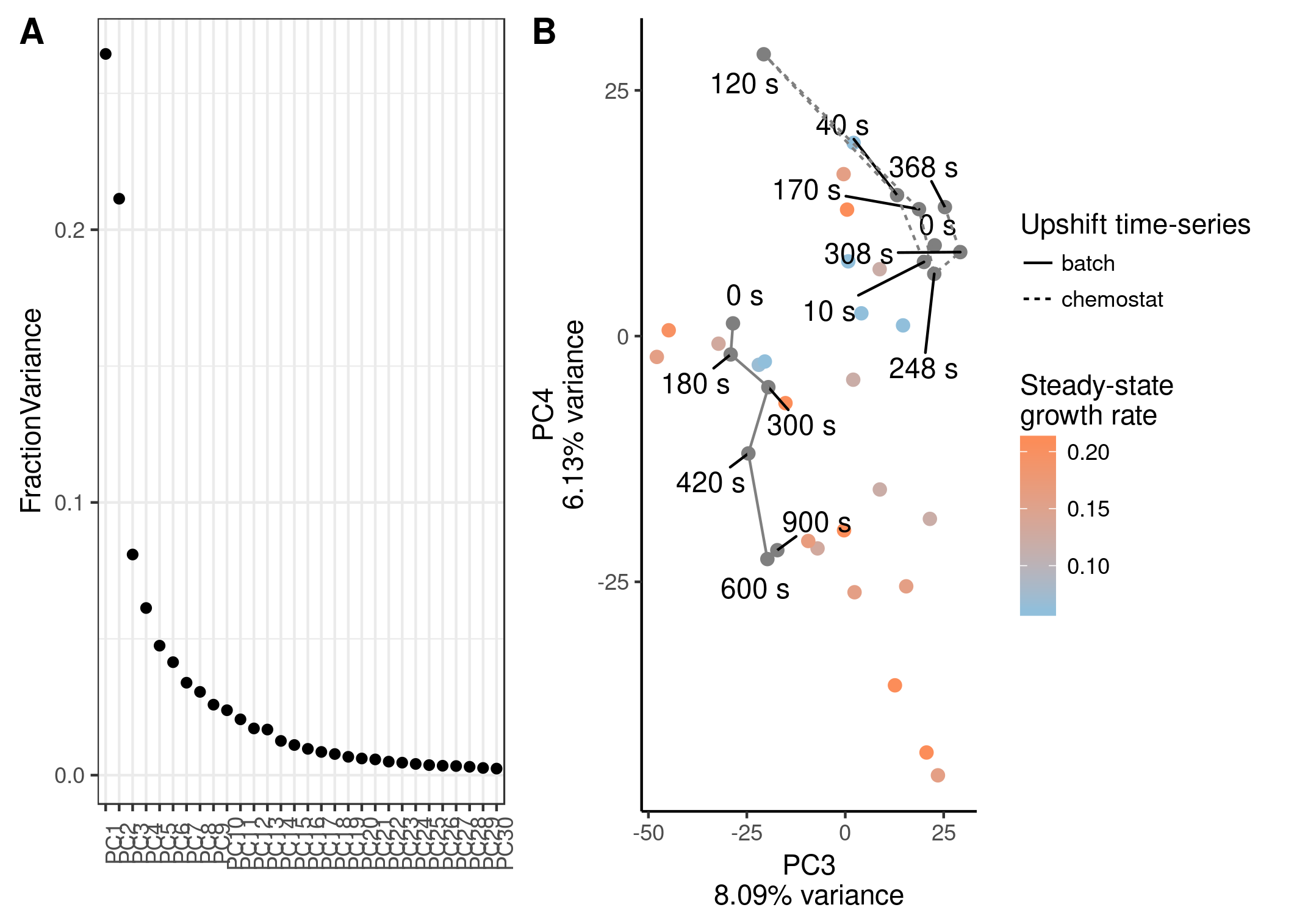

Supplement: S3 Fig — A) From the PCA analysis used in Fig 1C, the variance explained is plotted for the principal components, showing a steep decrease in explained variance after the first two components. B) The third and fourth components explain a small amount of the variance and are not readily interpretable with respect to the transcriptome changes in response to the changing growth rates in either steady-state or dynamic conditions. (TIFF) [file pgen.1007406.s017.tiff]

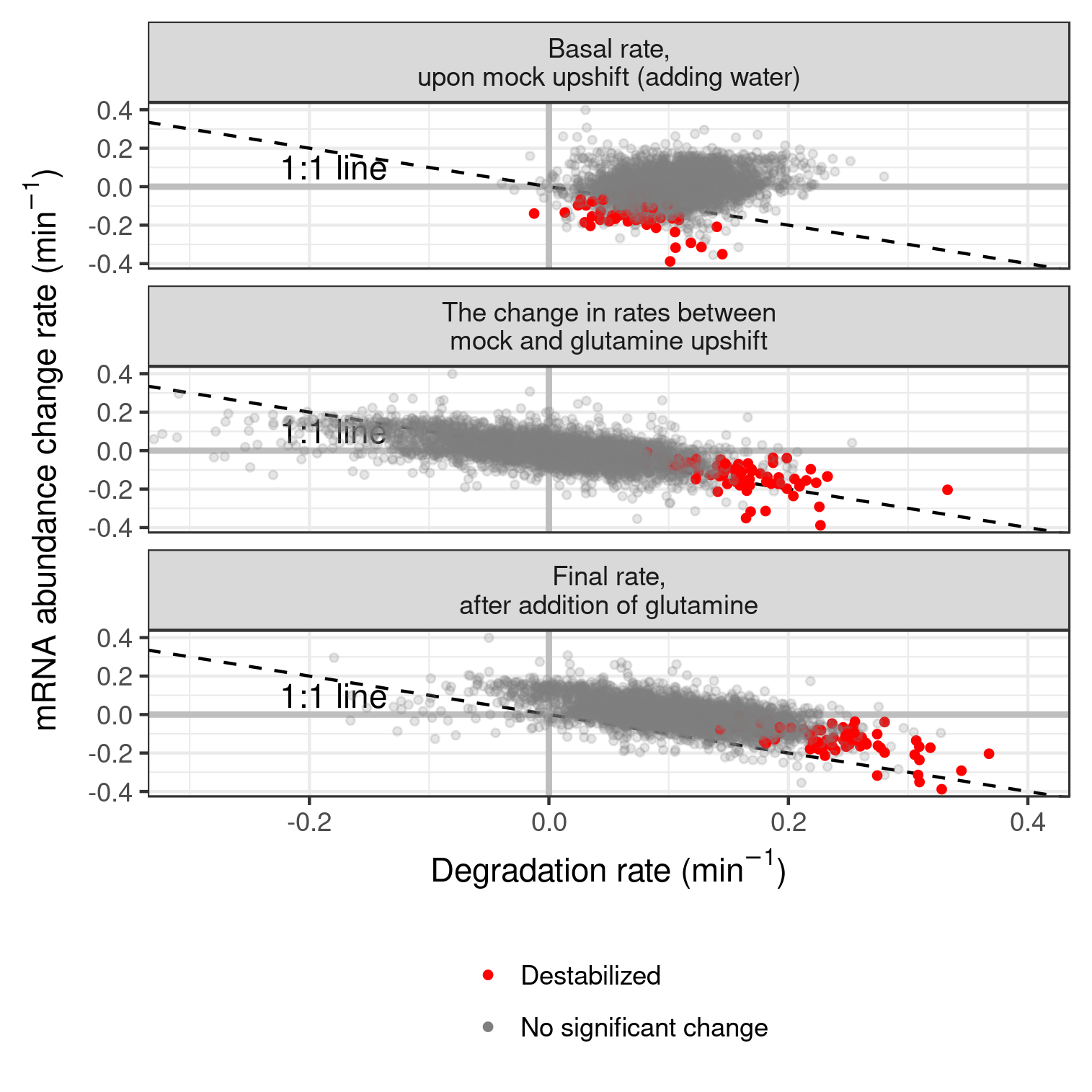

Supplement: S4 Fig — Comparisons of measured mRNA degradation from this study with mRNA abundance change rates from [25]. Pre-upshift degradation rates (top) don’t explain the abundance change. The degradation rate changes (middle, difference between pre and post upshift) and the post-upshift rates (bottom) are anti-correlated with the abundance changes. (TIFF) [file pgen.1007406.s018.tiff]

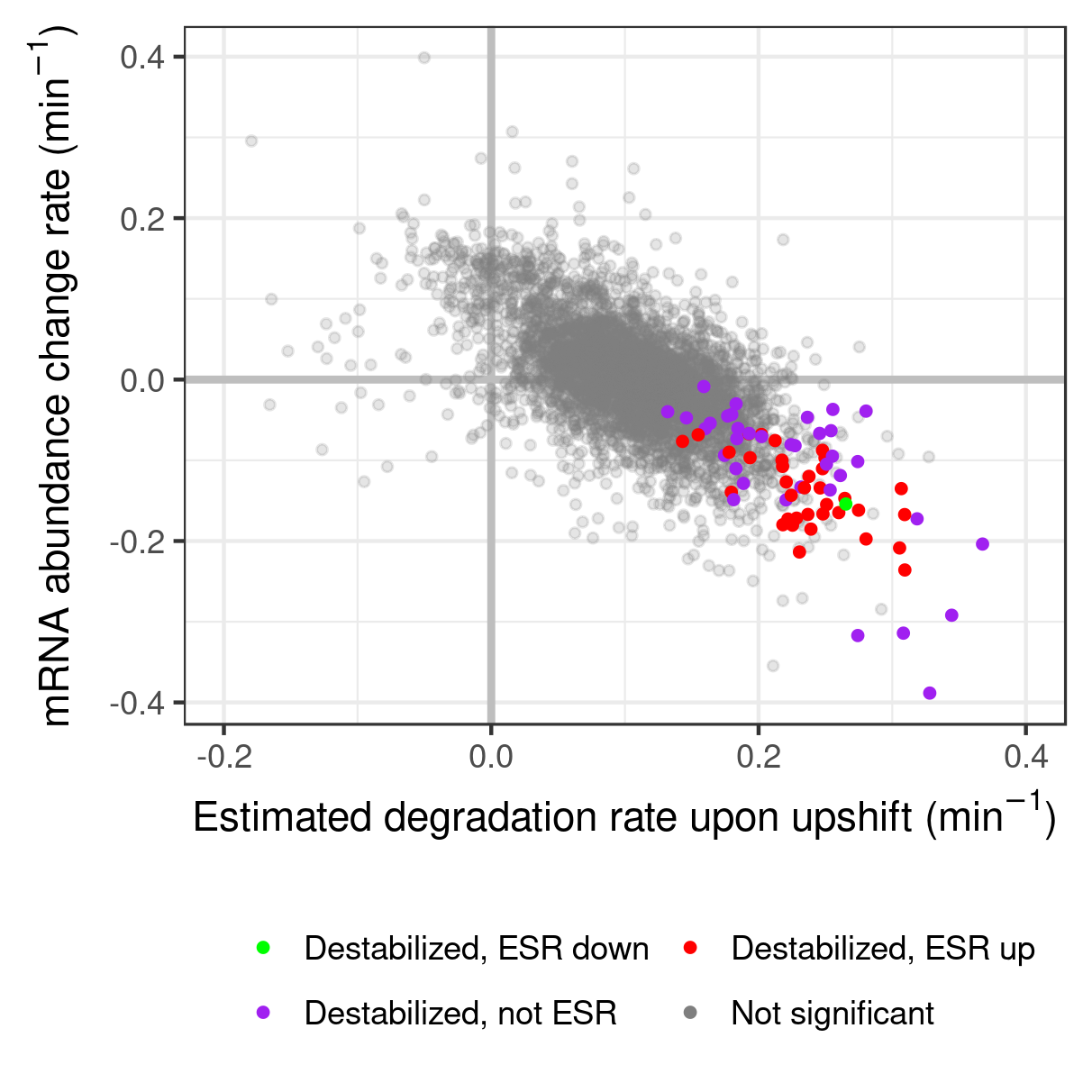

Supplement: S5 Fig — Comparisons of degradation rates from this study with mRNA abundance change rates from [25]. Destabilized transcripts are colored based on their membership in the ESR gene set, as described in the supplement of [86]. Many of the destabilized set are “ESR-up” genes, as they are increase in expression in response to stresses. (TIFF) [file pgen.1007406.s019.tiff]

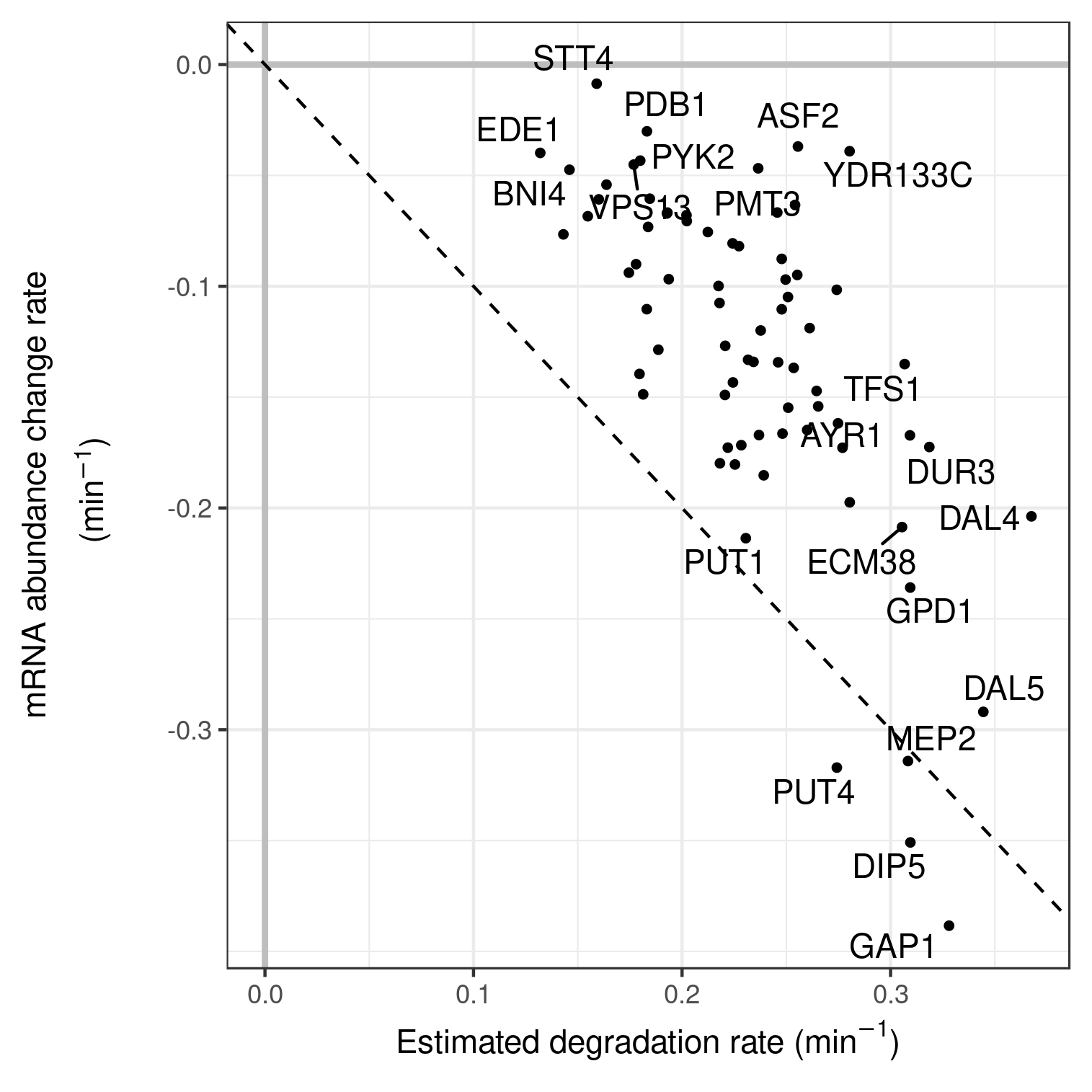

Supplement: S6 Fig — For each transcript the x-axis is the rate of degradation rate post-upshift and the y-axis is the mRNA abundance change rate [25] after the upshift. The dashed line is a 1:1 line of equality. (TIFF) [file pgen.1007406.s020.tiff]

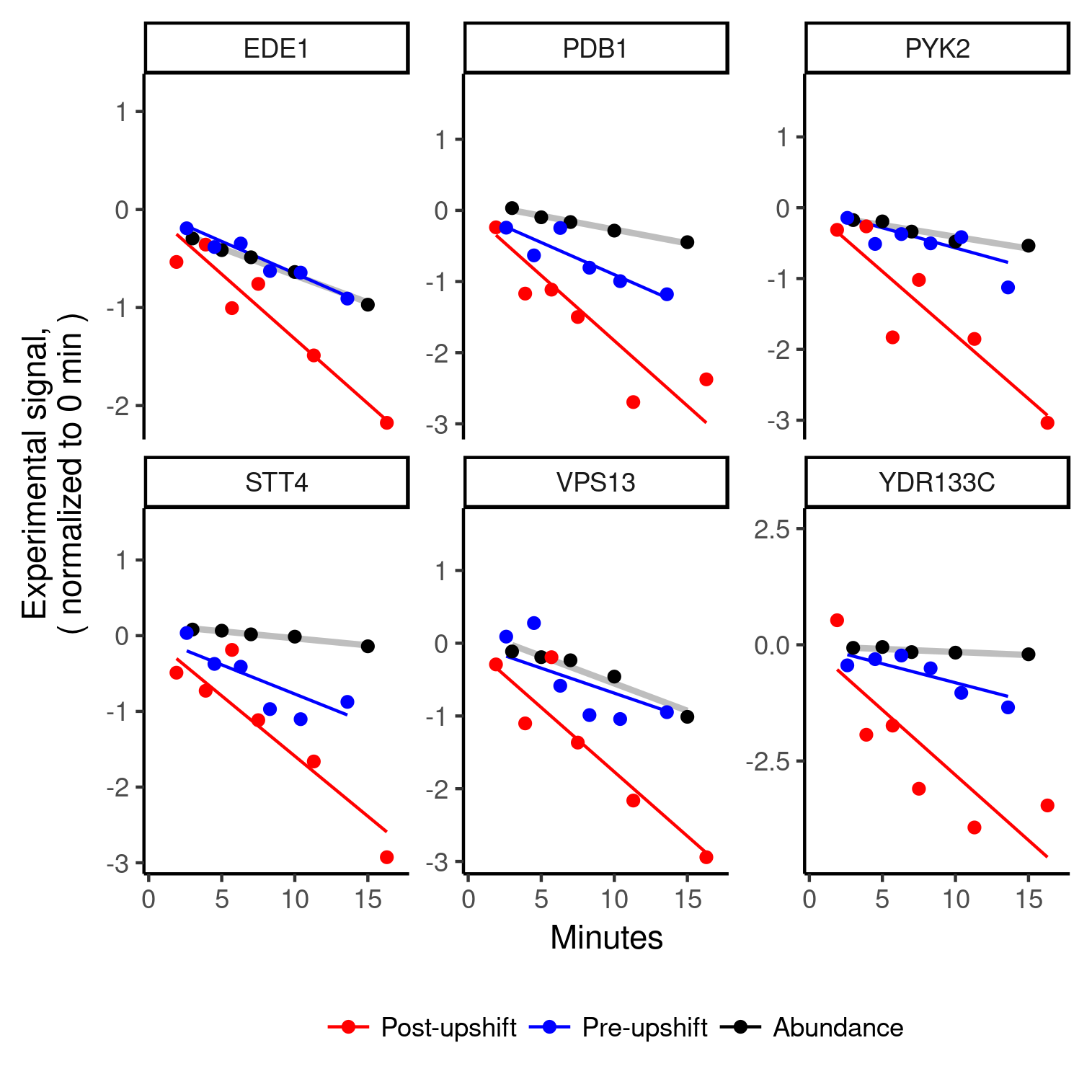

Supplement: S7 Fig — For several transcripts we found an increased rate of degradation post-upshift (red) compared to before the upshift (blue) but minimal changes in abundance (black). Each dataset is normalized to intersect at the same t = 0 intercept. (TIFF) [file pgen.1007406.s021.tiff]

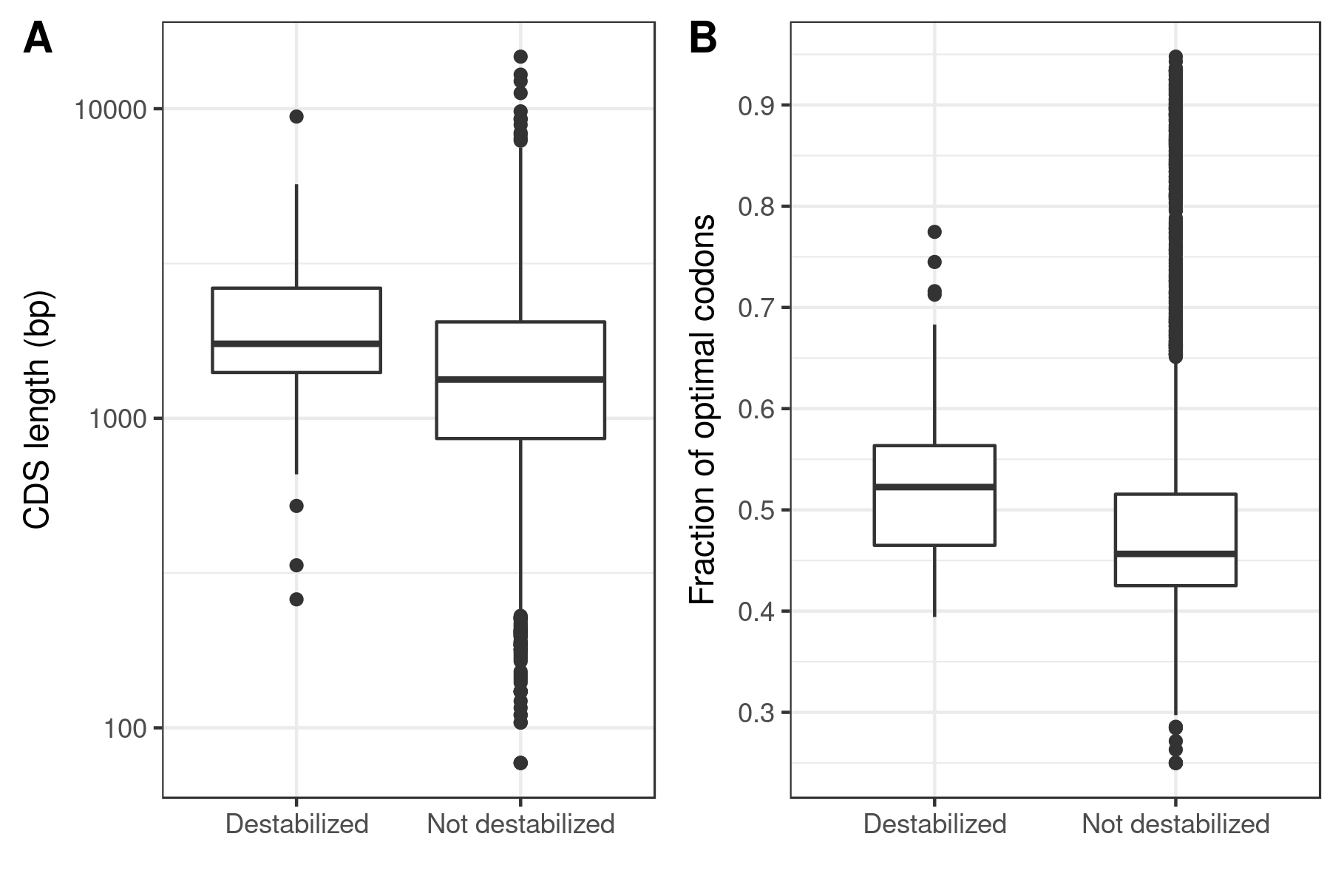

Supplement: S8 Fig — Comparisons of destabilized mRNAs with the rest of the transcriptome. A) Destabilized transcripts tend to have longer CDS lengths (p-value < 2 × 10^−5^ by Wilcoxon rank sum test). B) On average, the destabilized transcripts have more optimal codons than the rest of the transcriptome (p-value < 2 × 10^−8^ Wilcoxon rank sum test). The fraction of optimal codons per feature was obtained from the supplement of [58] using definitions from [111]. (TIFF) [file pgen.1007406.s022.tiff]

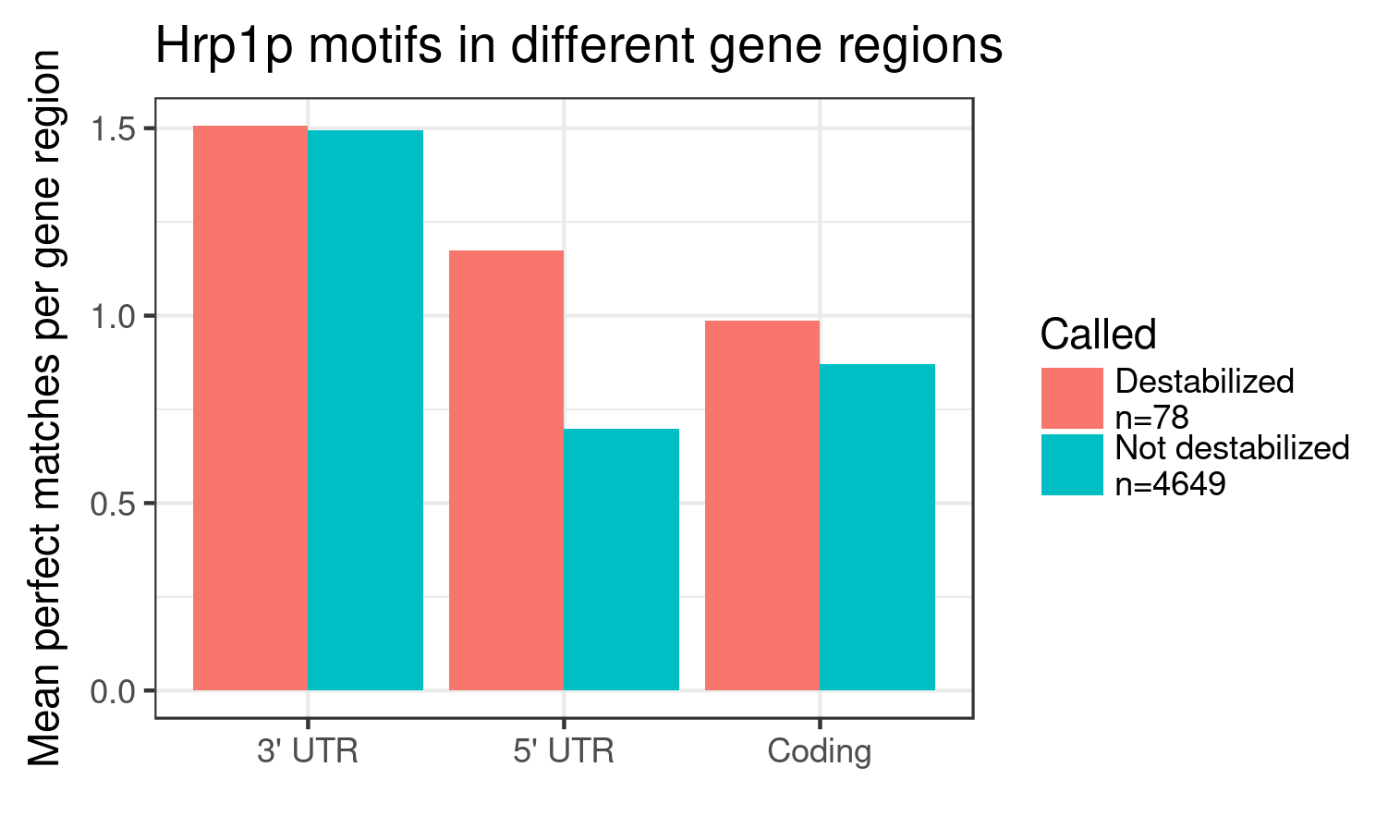

Supplement: S9 Fig — Sequences were analyzed for RBP binding motif enrichment using the AME program in MEME and significant hits confirmed using a logistic model predicting destabilization based on motif content per sequence length. Hrp1p is significantly (p < 0.0001) enriched in the 5’ UTRs of destabilized transcripts. Motif matches were counted using the GRanges package for the 5’ UTRs, 3’ UTRs, and coding sequence of transcripts using the largest isoforms detected in [104]. (TIFF) [file pgen.1007406.s023.tiff]

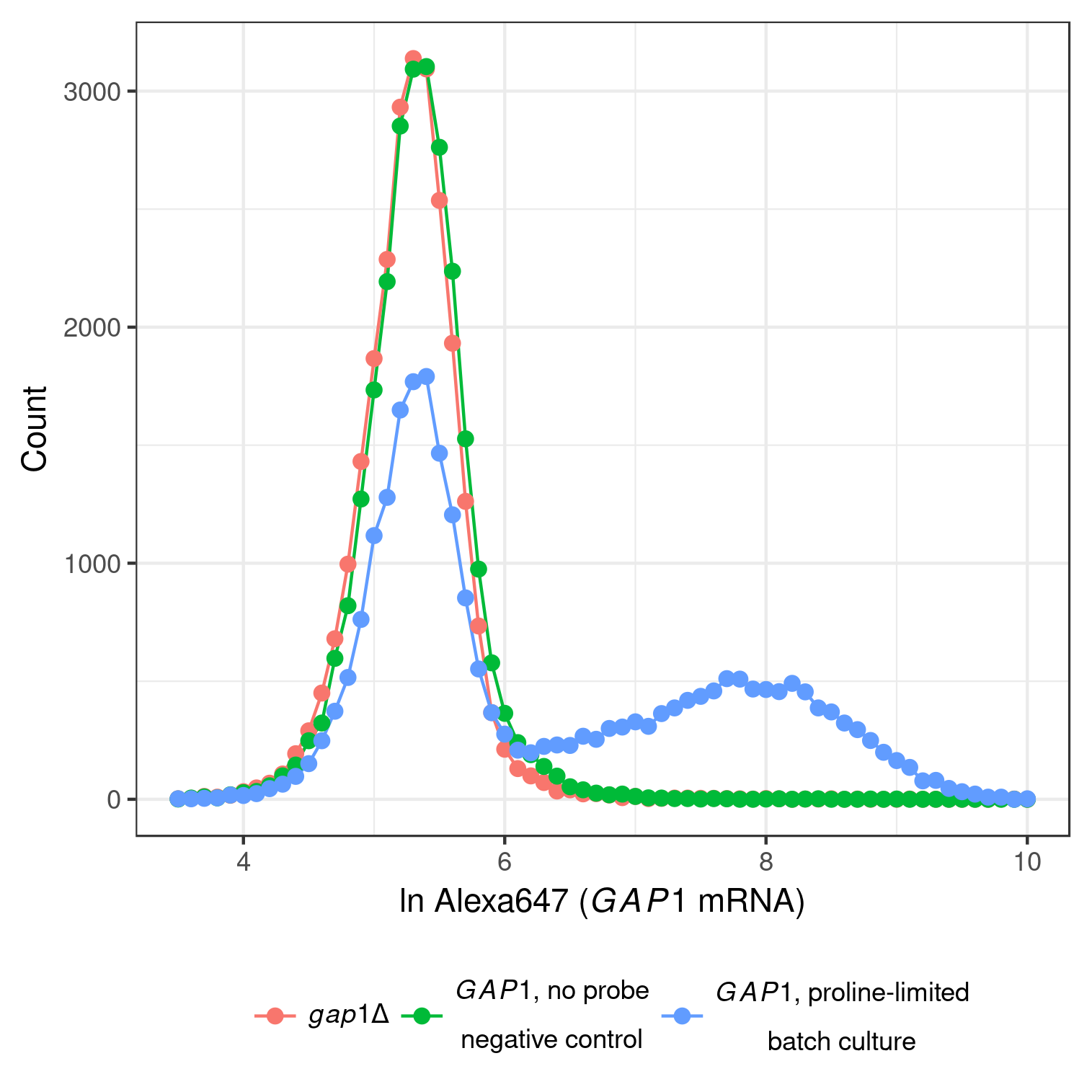

Supplement: S10 Fig — Wild-type or GAP1Δ cells were grown in proline-media. As seen in the positive control there is heterogeneity in the signal. (TIFF) [file pgen.1007406.s024.tiff]

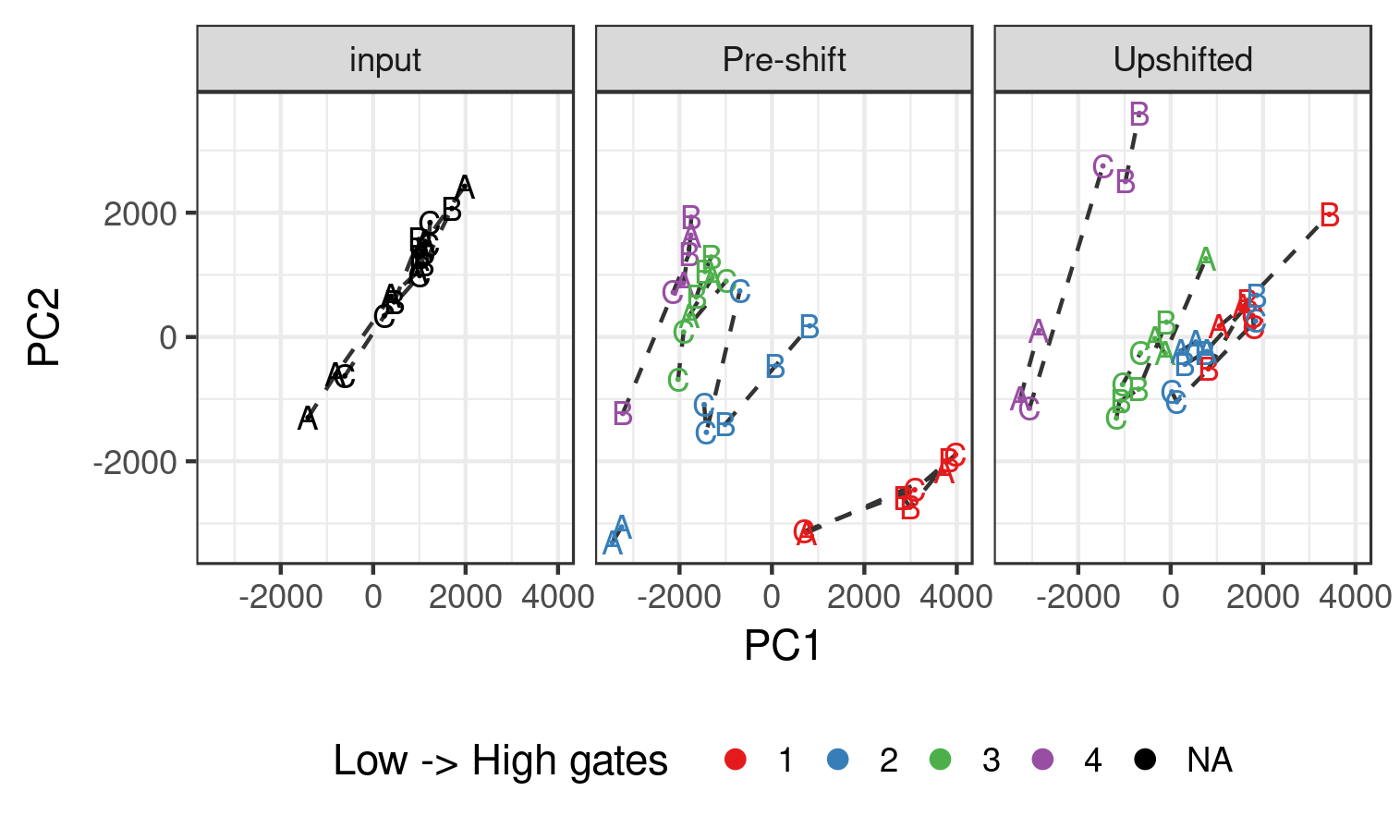

Supplement: S11 Fig — Each color is a type of sample, from low to high gates (with black denoting the input samples before sort). Technical replicates are connected by dashed lines, biological replicates are denoted with letters A B or C. The first two principal components show the separation of gates by signal intensity and reflects that the lower gates on the upshifted samples were very close (blue and red samples on far right panel), within the distribution of the negative population. This is consistent with their tight sampling of the “GAP1-off” population, as seen in Fig 4A. (TIFF) [file pgen.1007406.s025.tiff]

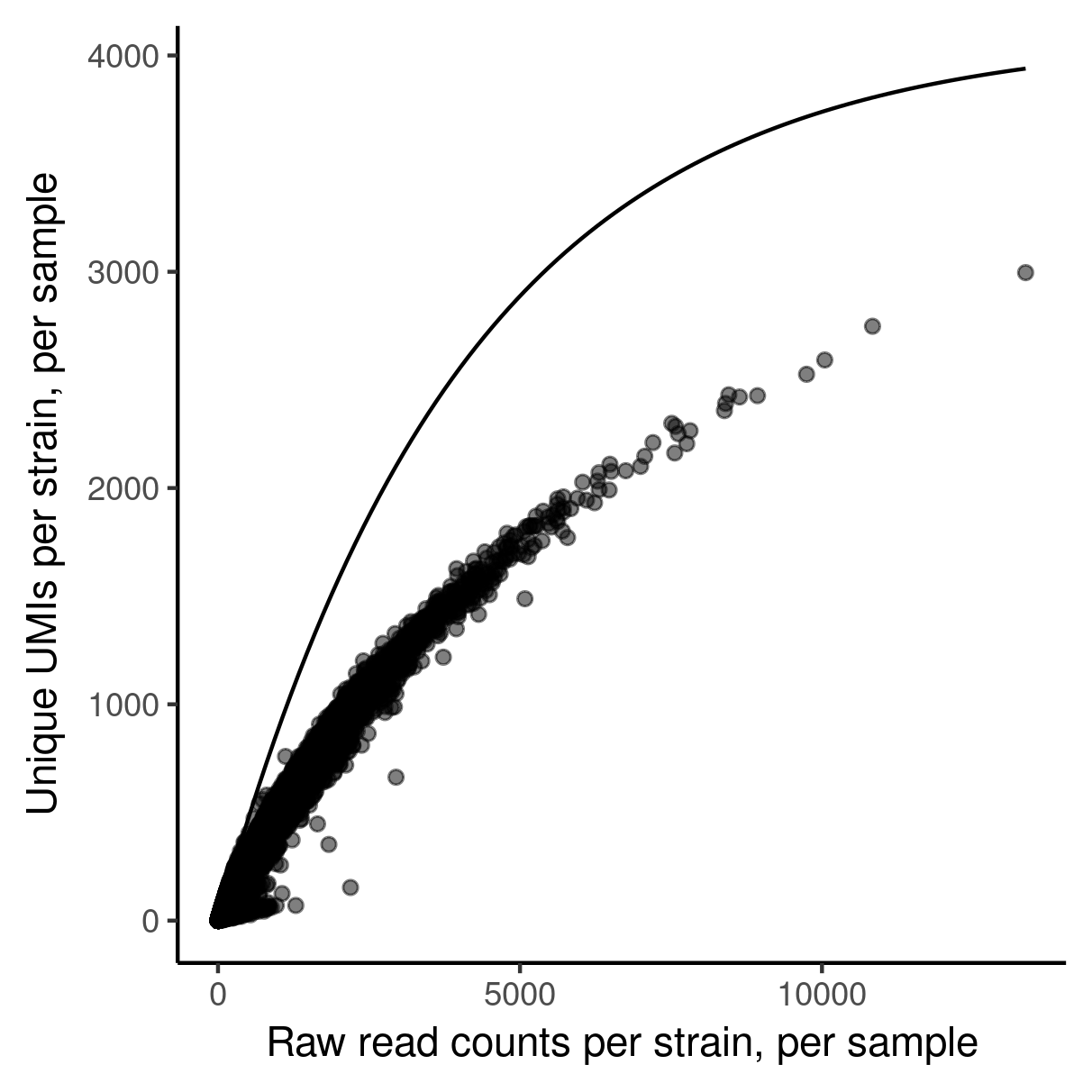

Supplement: S12 Fig — The solid-line curve denotes the theoretical expectation of total observations per UMI in a sample (x-axis) and the number of unique UMIs (y-axis). This curve shows how UMI-collisions are expected to depress the number of unique UMIs. Each point is from real data, with these two numbers tabulated for each combination of a sample and strain barcode. We see that these largely follow the curve of saturation of UMI-collisions, but that it falls well below the expectation of independent UMI-collision, thus we believe that there is an additional contribution of PCR-amplification noise (PCR duplicates). (TIFF) [file pgen.1007406.s026.tiff]

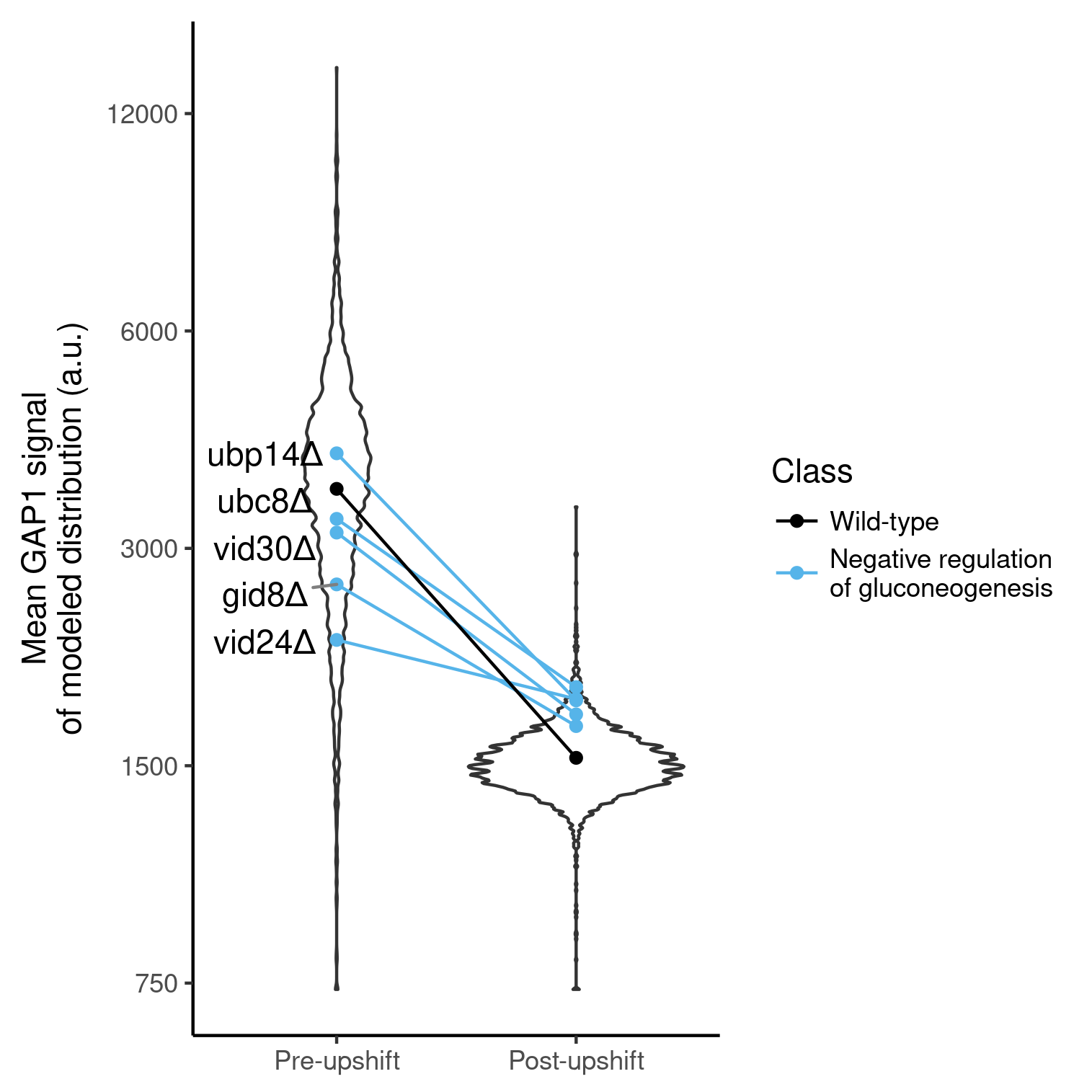

Supplement: S13 Fig — Knock-out mutants of negative regulators of gluconeogenesis are associated with higher estimated GAP1 mean after the upshift, by GSEA analysis of GO-terms (p-value < 0.05). (TIFF) [file pgen.1007406.s027.tiff]

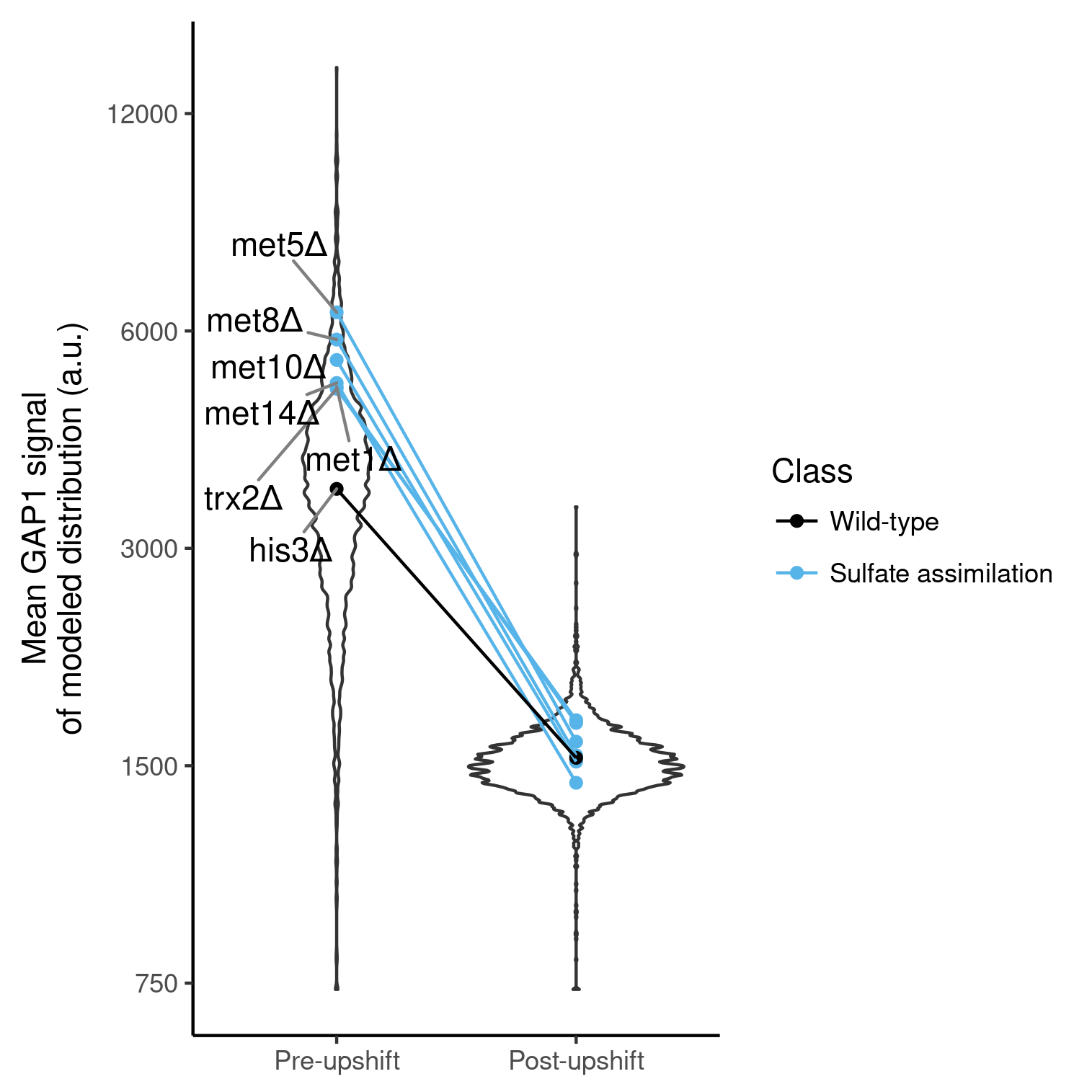

Supplement: S14 Fig — Knock-out mutants of involved in sulfate assimilation are associated with higher estimated GAP1 mean before the upshift, by GSEA analysis of GO-terms (p-value < 0.05). (TIFF) [file pgen.1007406.s028.tiff]

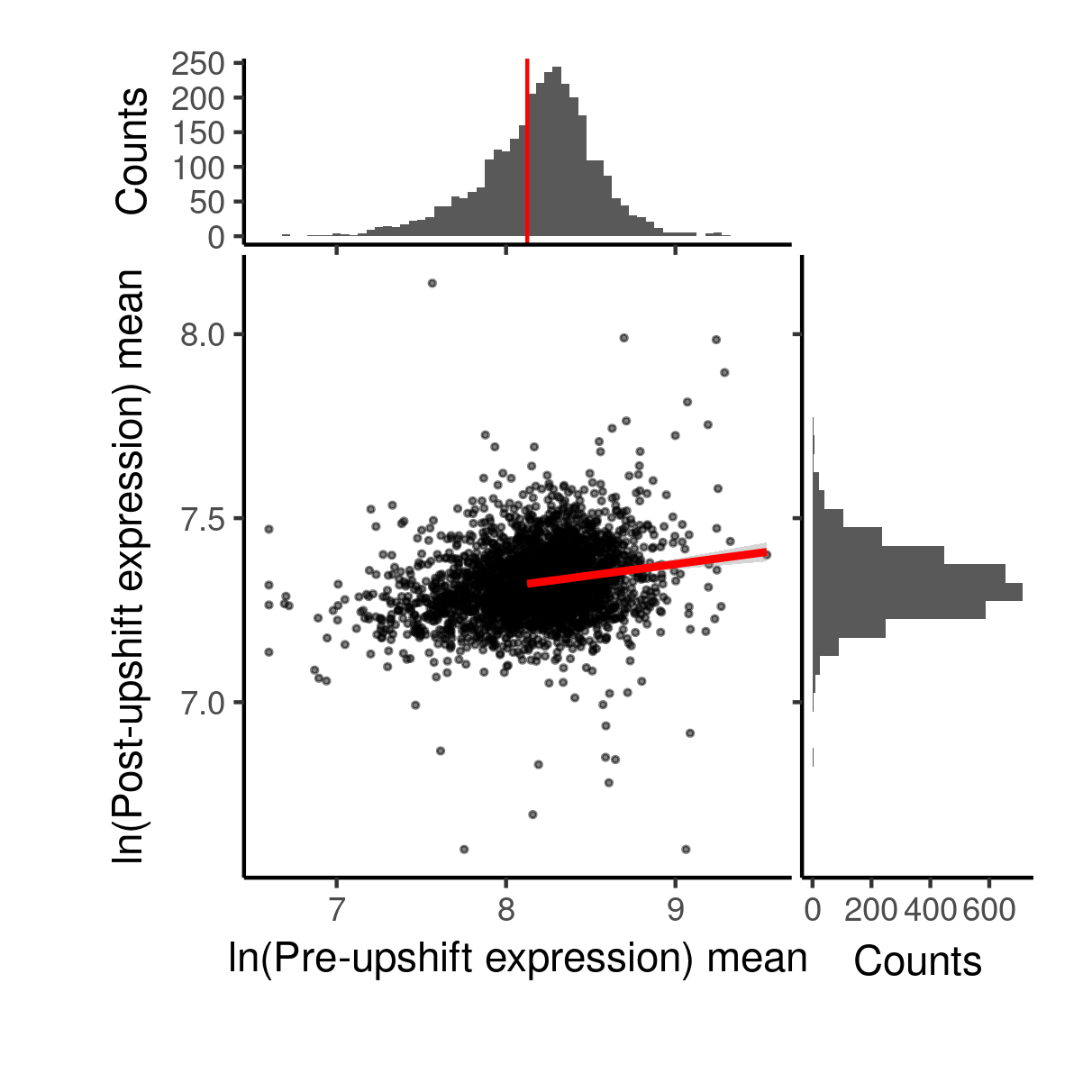

Supplement: S15 Fig — Scatter plot of the estimated means, with marginal histograms along top and right. Red vertical line on top histogram is a cut-off of GAP1 mRNA induction for this analysis, and is the mean of the fit to wild-type minus one standard deviation of that distribution. The red linear regression line is fit to all points above this threshold, in which expression was detected before the upshift. (TIFF) [file pgen.1007406.s029.tiff]

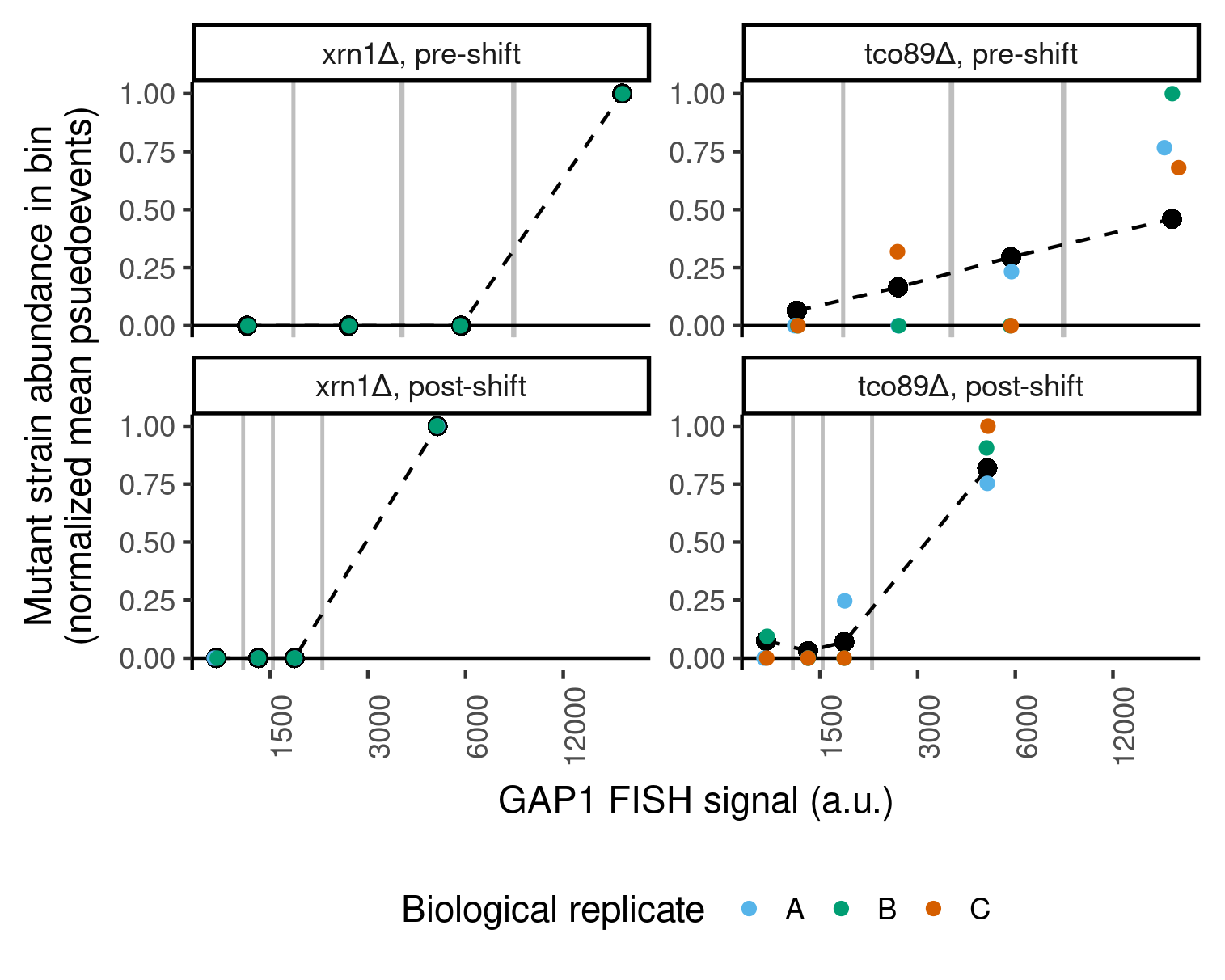

Supplement: S16 Fig — xrn1Δ mutant (left) is lowly abundant in the library and is only observed in the highest bin of GAP1 signal, consistent with the role of Xrn1p as a global exonuclease. tco89Δ is the only detected member that would abrogate TORC1 activity. This mutant (right) has elevated GAP1 mRNA before and after the upshift, consistent with the role of TORC1 in repressing the NCR regulon. (TIFF) [file pgen.1007406.s030.tiff]

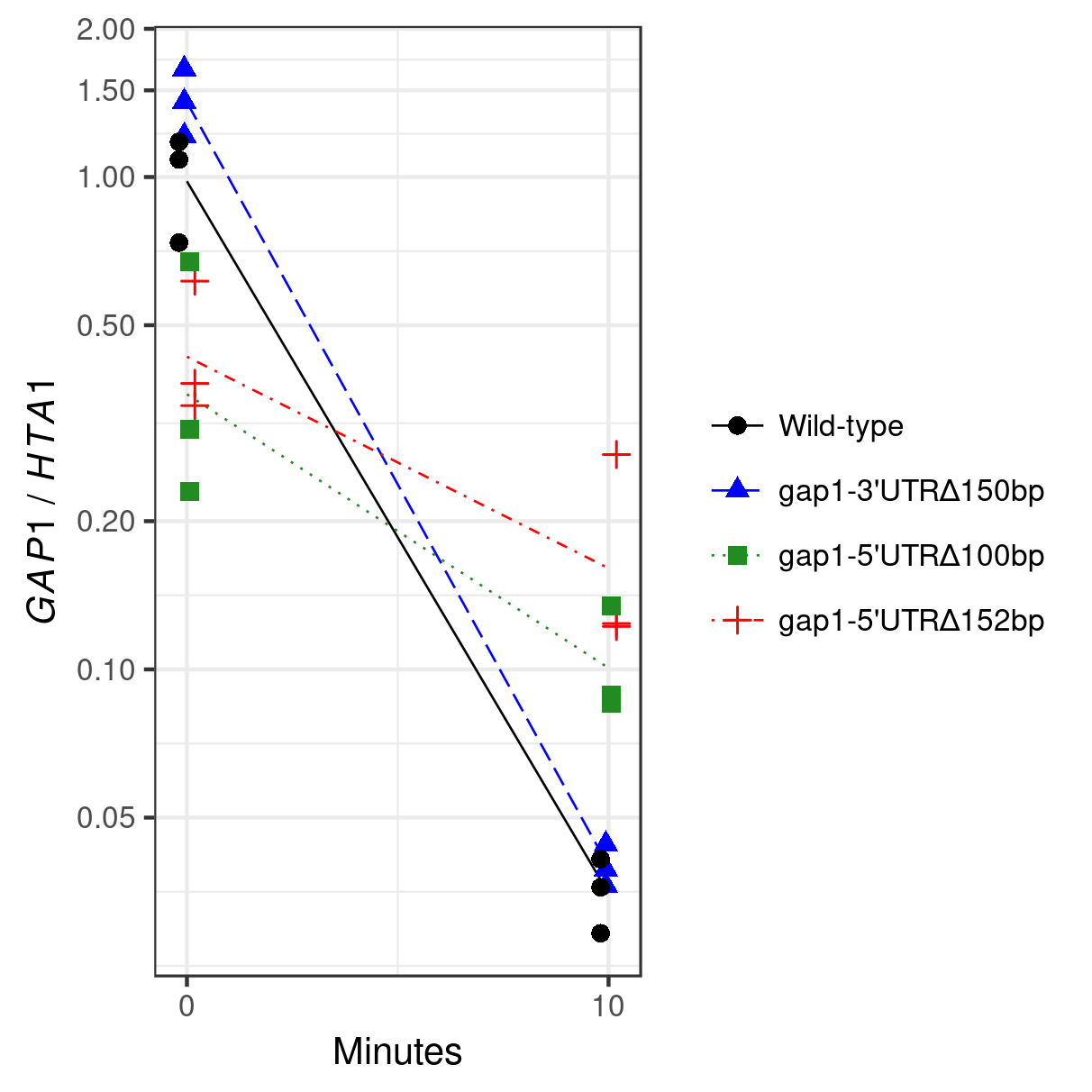

Supplement: S17 Fig — A deletion of 152bp 5’ of the start codon was also generated. We tested GAP1 dynamics in this strain as well, and found that it shares the same phenotype as a 100bp 5’ UTR deletion. Methods are the same as in Fig 5E, both 5’ UTR deletes are slowed in clearance, ANCOVA p < 0.05. (TIFF) [file pgen.1007406.s031.tiff]

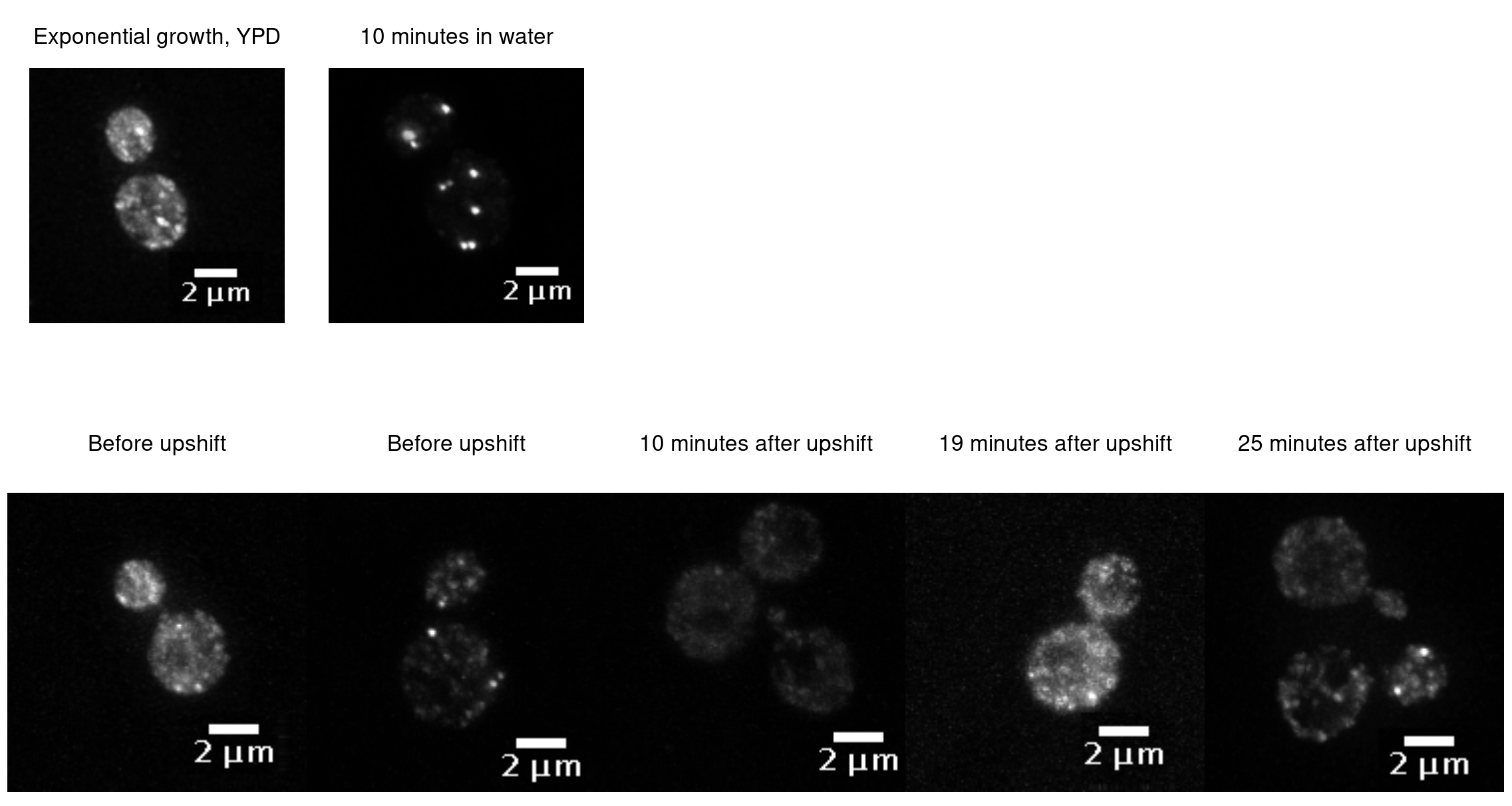

Supplement: S18 Fig — A strain containing a copy of Dcp2p-GFP expressed from a plasmid was grown in conditions of exponential phase in YPD or 10 minutes of starvation in water (first row) to confirm detection of processing-body foci using Dcp2-GFP. We do not see either formation or dissolution of Dcp2-GFP foci during the nitrogen upshift (bottom row). (TIFF) [file pgen.1007406.s032.tiff]
